# Straightforward Access to Polyfunctionalized δ-Lactams via Domino Aza–Michael/Thia–Michael/Aldol Sequence

**DOI:** 10.3390/molecules30102154

**Published:** 2025-05-14

**Authors:** Axelle Genty, Ismail Alahyen, Marie-José Tranchant, Jérôme Lhoste, Vincent Dalla, Catherine Taillier, Sébastien Comesse

**Affiliations:** 1 Université Le Havre Normandie, Normandie Université, URCOM UR 3221, F-76600 Le Havre, Francevincent.dalla-libera@univ-lehavre.fr (V.D.); 2 IMMM, UMR 6283 CNRS, Le Mans Université, F-72085 Le Mans, France; jerome.lhoste@univ-lemans.fr

**Keywords:** domino reaction, δ-lactams, *N*-alkoxyacrylamides, stereoselective synthesis, aza–Michael, thia–Michael, aldol

## Abstract

Domino reactions are powerful tools for the straightforward synthesis of complex molecules with a particular emphasis on functionalized azacycles. We report a contribution in this field, implemented via a new thia–Michael/aldol sequence between readily accessible *N*-alkoxyacrylamides and α,β-unsaturated carbonyls, for access to polysubstituted δ-lactams with acceptable-to-good yields and good selectivity. This method, initially developed in a two-component approach and characterized by the mildness of its reaction conditions, was shown to be compatible with various thiophenol derivatives and to employ a simple pre-thiasilylation step in a one-pot process. This further extension to the monotype aza–Michael/thia–Michael/aldol sequence establishes a proof-of-concept that acrylamides can react as both 1,3-bis-nucleophiles and 1,4-electrophiles in a single flask operation.

## 1. Introduction

The formation of C-S bonds still represents a key objective of organic synthesis due to the importance of the C-S linkage in chemical and biological compounds [1]. Thia–Michael additions, also known as sulfa–Michael additions, are a subset of the well-established conjugate 1,4-addition method that has proven to be one of the most reliable approaches to organosulfur compounds [2,3,4]. Thia–Michael reactions have also been used as effective devices for triggering cascade reactions [5]. In this context, domino thia–Michael/aldol reactions, which are analogous to the well-established oxa–Michael/aldol (DOMA) reactions, serve as valuable tools for organic chemists, giving access to sulfur-containing adducts with high efficiency [6].

**Scheme 1:**
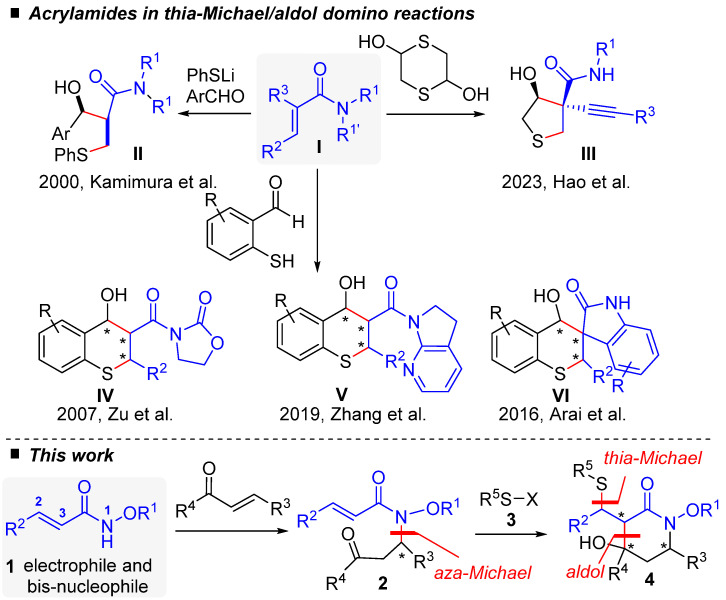
Acrylamide **I** in thia–Michael/aldol domino reactions: as reported in the literature [8,9,10,11,12] (upper) and in this work (lower). * for asymmetric carbon.

Over time, type **I** acrylamides have emerged as useful domino partners for the synthesis of many N-heterocyclic frameworks [7]. However, surprisingly, there is only a limited number of reports of domino thia–Michael/aldol reactions involving acrylamides, with no examples in which the nitrogen atom from the acrylamide moiety is incorporated into the resulting cyclic structure (Scheme 1). In 2000, Kamimura and coworkers pioneered the field and showed that the reaction between acrylamide **I** and aromatic aldehydes in the presence of lithium thiophenolate gave thia–Michael/aldol acyclic product **II** in good yields, whereas aliphatic aldehydes gave poor results [8].

Subsequent investigations have concentrated on substrates that embed the thiol and aldehyde functions in the same molecule, leading to a variety of thiacyclic frameworks in which the amide unit resides as substituent. Most of the relevant examples dealt with diverse enantio- and diastereoselective thia–Michael/aldol reaction sequences from 2-mercaptobenzaldehyde derivatives catalyzed by bifunctional hydrogen-bonding organocatalysts, which provided efficient and straightforward entry to a variety of 6-membered thiochromenes, of types **IV** and **V [9,10]**. An alternative organometallic approach, using a chiral PyBidine−Ni(OAc)_2_ catalyst system, provided access to analogous spirooxindole derivative **VI** in both high yields and with good stereoselectivity [11]. More recently, the organocatalytic, stereoselective access to substituted tetrahydrothiophene **III** was also achieved through another type of thia–Michael/aldol reaction with 1,4-dithiane-2,5-diol [12]. As previously mentioned, alternative and complementary examples offering access to azacycles through the incorporation of the N-amidic atom within the cyclization process have not yet been realized. This would necessitate changing the connectivity of the reaction partners by, specifically, tethering the nitrogen-containing group to the aldehyde electrophilic functionality instead of the sulfur moiety, a requirement that our recently developed methodologies can, in principle, easily fulfill. Indeed, in the past few years we have been engaged in a research program aimed at revealing the full potential of *N*-alkoxyacrylamide synthons as a highly modulable multipolar platform for domino chemistry [7]. In a seminal report taking advantage of the enhanced nucleophilic reactivity of the *N*-alkoxy group, we documented a domino aza–Michael/Michael sequence leading to functionalized piperidinones that exploited the formal 1,4-dipolar character of the readily accessible alkoxyacrylamide synthon **I [13]**. More recently, we reported an original PPh_3_ organocatalyzed domino aza–Michael/Morita–Baylis–Hillman reaction between **I** and α,β-unsaturated carbonyls that revealed the 1,3-bis nucleophilic behavior of component **I** [14]. In the general context outlined above, we here disclose an extension of these works in the form of a new thia–Michael/aldol domino reaction, initially employing alkoxyacrylamide Michael adduct **2** and thiophenol derivative **3**, to provide access to polysubstituted piperidin-2-one **4** in good yields and with good selectivity (see the lower part of Scheme 1). An adaptation of this two-component methodology to a monotype aza–Michael/thia–Michael/aldol sequence establishes a proof-of-concept that acrylamide **1** can serve both as a 1,3-bis-nucleophile and a 1,4-electrophile in a one-pot process when sequentially reacted with an α,β-unsaturated carbonyl compound and a suitable nucleophile.

## 2. Results and Discussion

### 2.1. The Optimization of the Thia–Michael/Aldol Domino Sequence

We first assessed the thia–Michael/aldol domino sequence with alkoxyacrylamide **2a** as the model substrate. The latter was prepared in one step by the aza–Michael addition of the parent, alkoxyacrylamide **1a,** on crotonaldehyde using pyrrolidine as an aminocatalyst (70% yield). The first set of experiments were directly conducted with thiophenol and only gave disappointing results. In the absence of any additive, a complete lack of reactivity was observed, while the use of basic additives showed poor reactivity, with only the formation of small amounts of the linear product **5a**, likely resulting from the early protonation of the enolate intermediate generated during the initial thia–Michael addition step by unreacted thiophenol (the results are not shown herein, see Supplementary Materials for more data). The need to avoid this undesirable acid–base reactivity urged us to consider the adoption of a silyl transfer strategy, which directed our attention toward the commercially available PhS-TMS **3a** as a pre-nucleophile. Surprisingly, some conversion was observed in the absence of an additive, again leading to the formation of small amounts of uncyclized compound **5a** (Table 1, Entry 1). Conversely, and as hoped, in the presence of 20 mol% TBAF (tetrabutylammonium fluoride) as the Lewis base, the desired reactivity took place to give acceptable yields of the expected product, **4a**, as an almost equal mixture of two stereoisomers, **4a_1_** and **4a_3_** (d.r. 44:56, Entry 2). The use of other solvents (not shown herein—see Supplementary Materials) was found to be detrimental to the reaction yield. Suspecting that post-reaction epimerization promoted by a strong basic medium associated with the fluoride anion source might be, at least in part, responsible for this non-stereocontrolled outcome, we next considered the evaluation of less basic additives. We commenced with the anhydrous and weakly basic TBAT (tetrabutylammonium difluorotriphenylsilicate), which was found to convey very poor reactivity, with no formation of product **4a** (Entry 3). We then turned to alternative oxyanion Lewis bases of intermediate and modulable basicity, with respect to these two extreme fluorinated reagents. The subsequent screening rapidly validated our above hypothesis. Testing *t*-BuOK first, the desired cyclic product, **4a,** was isolated, with a promising yield of 45% and substantially enhanced and encouraging diastereoselectivity. We also observed a change in the identity of the second diastereoisomer, with **4a_2_** being obtained in lieu of **4a_3_** (ratio **4a_1_**/**4a_2_** = 84:16—Entry 4). These apparent trends towards “reactivity–stereoselectivity conflicts”, as well as subtle variations in the formation of the minor diastereoisomer **4a_2_** versus **4a_3,_** were further confirmed via the examination of softer carbonate bases (Entries 5–7), of which, K_2_CO_3_ provided the most promising result (37% yield, **4a_1_**/**4a_3_** = 95:05—Entry 7) (The superior effect of the potassium cation has been observed several times and seems to be common feature in domino reactions of dipolar *N*-alkoxycarboxamide synthons [13,15], assumably as a result of the well-adjusted templating properties of K^+^ for this function class). At this juncture, it clearly appears that the whole reaction profile is subject to strict control, imparted by the basic properties of the additive, which must be well balanced to promote the effective activation of the sulfur nucleophile (i.e., with TBAF) while mitigating, as much as possible, reaction product epimerization (i.e., with K_2_CO_3_). Regarding these considerations, we were pleased that increasing the temperature and the quantity of K_2_CO_3_ to one equivalent (Entries 7–9) boosted nucleophilic activation to raise the product yields to synthetically useful levels, with only a slight alteration of the stereocontrol (62% yield, **4a_1_**/**4a_2_** = 87:13—Entry 9). After screening different solvents and temperatures (compare Entries 9–16), the best conditions for providing the highest possible yield and the d.r. (diastereomeric ratio) were found, i.e., C(**2a**) = 0.2 M in THF, PhS-TMS (1.2 equiv.), K_2_CO_3_ (1 equiv.) at 40 °C (68% yield, **4a_1_**/**4a_2_** = 88:12—Entry 16).

**Scheme 2:**
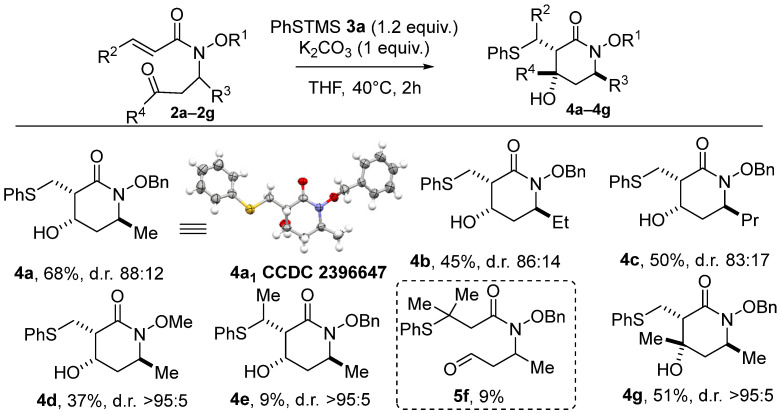
The scope of the domino thia–Michael/aldol process with various acrylamides.

Given that three stereogenic centers are present on cyclic product **4a**, four diastereomers could theoretically be formed, but only three of them were observed in the conditions tested. Gratifyingly, using our set of optimized conditions produced a mixture of only two diastereoisomers, of which **4a_1_** was formed predominantly in all cases. In the rest of the study, the term “d.r.” will refer to the mixture of these two diastereomers. The X-ray structure of the major diastereoisomer, **4a_1,_** reveals a *syn* relationship between the PhSCH_2_ moiety and the OH, as well as a *trans* relationship with the methyl group (see the X-ray structure of **4a** in Scheme 2). The relative stereochemistry of the minor diastereoisomers, **4a_2_** and **4a_3,_** was also elucidated, using NMR NOESY experiments and comparisons with previous experiments (see Supplementary Materials for details).

### 2.2. The Exemplification of the Thia–Michael/Aldol Domino Sequence

With the optimized conditions in hand, we turned our attention to the scope of this domino reaction. First, a reaction between PhS-TMS as the model pre-nucleophile and various acrylamides **2** was considered (Scheme 2). As previously, only two diastereoisomers were systematically produced. The modulation of R^3^ (Et and Pr) led to products **4b** and **4c**, with a stereoselectivity similar to that found previously but with lower yields compared to **4a** (45% and 50%, respectively). Replacing the benzyloxy moiety by a methoxy group onto the nitrogen atom reduced the yield to 37%, but the cyclic product **4d** was isolated with a high >95:5 d.r. The significant decrease in the reaction efficiency when going from a benzyloxy to a methoxy amide lines up with our recent MBH approach [14] but stands in opposition with annulations based on the ambident Nu then E reactivity of dipolar alkoxycarboxamide synthons, where a *N*-methoxy motif was found much more effective [13,15]. As expected, the thia–Michael addition step proved sensitive to steric hindrance, with no product being formed when the acrylamide moiety was mono- or dimethylated in the β position (Yield estimated from ^1^H analysis of the crude, isolation of the product **4e** in completely pure forms having failed). In contrast, the use of the *(E)*-pent-3-en-2-one as a reaction partner was well tolerated, and the desired piperidin-2-one **4e** with a quaternary stereocenter was obtained in a satisfactory yield of 51% and a high d.r. (>95:5). The stereochemistry of product **4e** has been proposed by analogy with products formed from analogous carboxamido-aldehyde substrates.

**Scheme 3:**
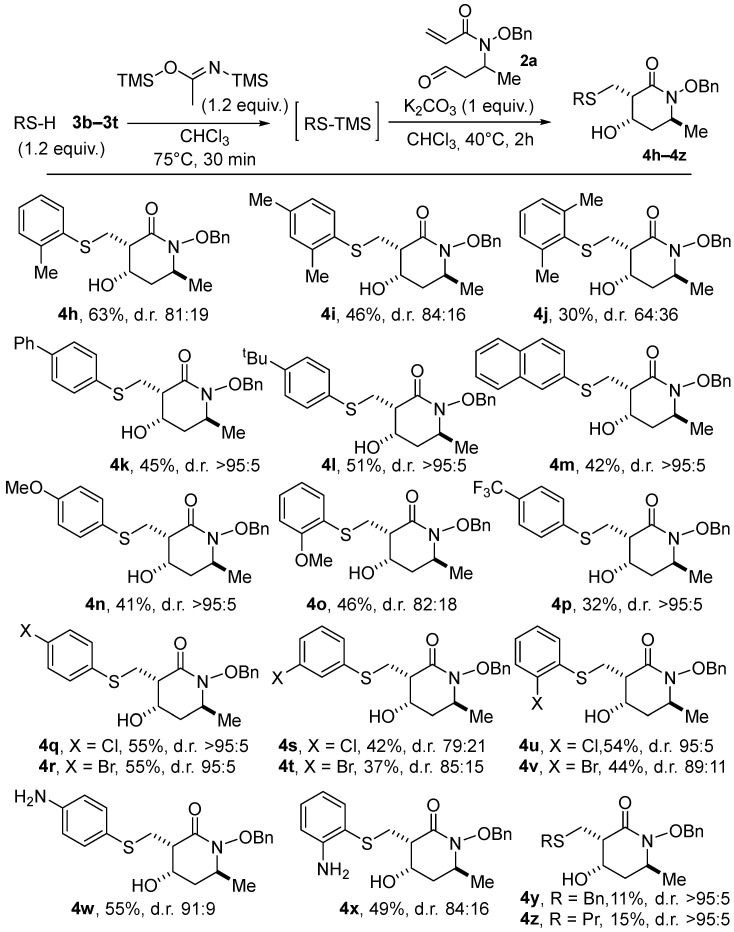
Scope of the one-pot silylation/thia–Michael/aldol process with various thiols.

We then focused on probing the scope of thiols as nucleophiles (see Scheme 3). Given that most silylated thiols are not available commercially, we first modified the sequence to incorporate a pre-silylation step in a one-pot process. Starting from the parent thiophenols, we executed the silylation using a revised protocol developed by Khodair and co-workers [16]. The proof-of-concept was first demonstrated using PhSH (1.2 equiv.) and bis(trimethylsilyl)acetamide (BSA, 1.2 equiv.) as the silylating agent in CHCl_3_ at 75 °C. Once silylation was complete (ca. 30 min.), the temperature was lowered to 40 °C, and the alkoxyacrylamide **2a** and K_2_CO_3_ (1 equiv. of each) were added. With this new one-pot silylation/thia–Michael/aldol sequence, lactam **4a** was obtained with the yield and the d.r. (66%, 90:10) being very similar to that of our initial protocol using PhS-TMS in THF (68%, d.r. 88:12, Table 1, Entry 16) (In comparison, when conducted in previously optimized THF solvent starting from thiophenol in the presence of BSA, lactam **4a** was isolated in 51% yield, instead of 68% with PhS-TMS; see Supplementary Materials). With these conditions established, various thiols were tested, and the results are summarized in Scheme 3.

Furthermore, 2-Methylthiophenol provided the desired cyclic product, **4f,** in both a slightly diminished yield (63%) and diastereomeric ratio (81:19). The presence of an additional methyl substituent in either the para or ortho positions led to lactams **4g** and **4h** with lower yields and selectivity (46%, d.r. 84:16 for **4g** and 30%, d.r. 64:36 for **4h**), probably due, for the latter, to a greater steric penalty in the thia–Michael addition. Other aryl or alkyl groups in the para position of the aromatic ring were well tolerated, and adducts **4i**, **4j**, and **4k** were isolated in decent yields with high diastereomeric ratios (42–51%, d.r. >95:5). Reactions with thiophenols substituted by electron-donating or -withdrawing groups proved also effective, albeit in a lower yield in the case of the most deactivating -CF_3_ group (compare 41% for **4l**, 46% for **4m**, and 32% for **4n**). It should be noted that this trend was increased to an extreme extent in the case of the thiophenol p-NO_2_, so that no trace of the lactam product was observed. To summarize, the presence of substituents either on the electrophilic β-carbon of the Michael acceptor or in the ortho position of the thiol derivatives, as well as electron-withdrawing substituents on the latter, are limiting factors of the process. In an additional subset of examples, various substituted chloro- and bromo-thiophenols were also successfully employed as nucleophiles, with the meta-substituted products **4q** and **4r** surprisingly obtained in lower yields and selectivity than their ortho **4o**–**4p** or para counterparts **4s**–**4t**. Satisfyingly, piperidin-2-ones **4u** and **4v,** bearing an additional free amino group on the aromatic ring, were formed in good yields (55% and 49%, respectively), exemplifying the remarkably functional group compatibility and chemoselectivity of the process (Monitoring by ^1^H NMR of the initial silylation step proved to proceed selectively on the thiol moiety). In contrast, the use of alkyl thiols only afforded poor yields in the corresponding cyclized products **4w** (11%) and **4x** (15%), supposedly due to the less favorable desilylation of the substrates. It is important to note that, in the majority of the cases examined above, a high degree of diastereoselectivity was obtained.

### 2.3. Sequential One-Pot Aza–Michael/Thia–Michael/Aldolization Sequence

**Scheme 4:**
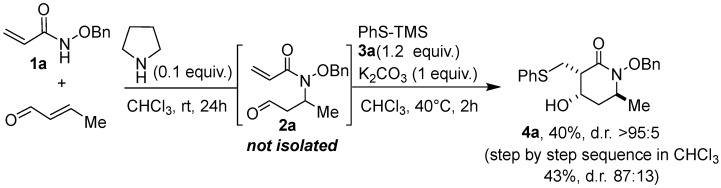
One-pot aza–Michael/thia–Michael/aldol sequence.

In a final attempt, we sought a one-pot aza–Michael/thia–Michael/aldolization sequence that would allow the direct transformation of benzyloxyacrylamide **1a** into the polysubstituted lactam **4a**, taking advantage of the fact that the aza–Michael addition leading to substrate **2** is better performed in chloroform, which is also a routine solvent for the present thia–Michael/aldol sequence (Scheme 4). First, the aza–Michael reaction was carried out between **1a** and crotonaldehyde in the presence of pyrrolidine (0.1 equiv.) in CHCl_3_ at room temperature for 24 h. Then, PhS-TMS **3a** (1.2 equiv.) and K_2_CO_3_ (1 equiv.) were added, and the temperature was increased to 40 °C for 2 h. Very satisfactorily, product **4a** could be obtained with a yield of 40% as a single isolated diastereoisomer (**4a_1_**). This result was compared with the step-by-step sequence in CHCl_3_, i.e., including the preparation and purification of the aza–Michael intermediate, **2a,** followed by the thia–Michael/aldol domino reaction described above, leading to lactam **4a** with a 43% overall yield and a 87:13 d.r. (first step 70% and second step 63%). Consequently, carrying out this reaction sequence as a one-pot process is of significant interest as it enables compound **4a** to be produced with the same efficiency as the “step-by-step” process, while preventing the isolation of the aza–Michael intermediate and ensuring the formation of a single diastereoisomer. In addition, this refined approach sheds light on the flexibility and modularity perspectives of the method.

### 2.4. Proposed Mechanism

**Scheme 5:**
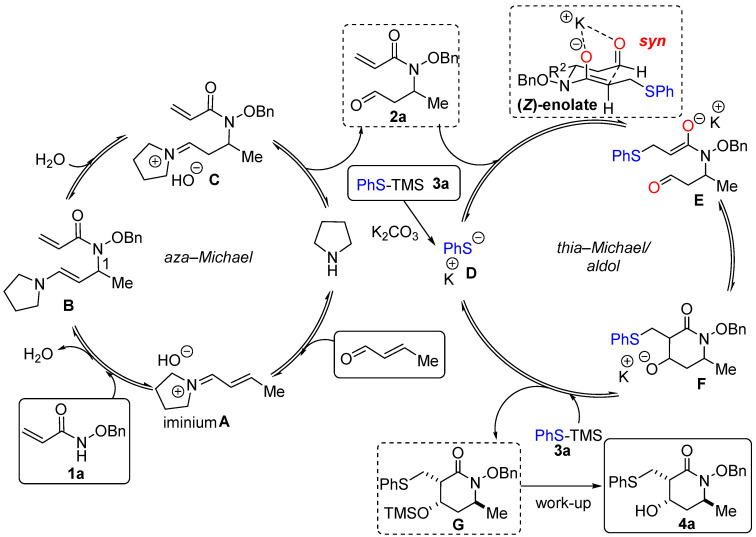
The proposed mechanism for the sequential one-pot aza–Michael/thia–Michael/aldol sequence.

The proposed mechanism for the sequential one-pot aza–Michael/thia–Michael/aldol sequence is presented in Scheme 5. The process begins with the condensation of pyrrolidine and crotonaldehyde, followed by the 1,4-addition of alkoxyacrylamide **1a** onto iminium ion **A**. After hydrolysis, aza–Michael adduct **2a** reacts with thiolate **D**, generated in situ by a reaction between K_2_CO_3_ and PhS-TMS. An intramolecular aldol reaction furnishes cyclic intermediate **F** which can then react with PhS-TMS to form silylated adduct **G**. After work-up and flash chromatography, alcohol **4a** was isolated. As previously mentioned, the major diastereoisomer **4a_1_** presents a *syn* relationship between the PhSCH_2_ moiety and the OH, as well as a *trans* relationship with the methyl group (see Scheme 2). This outcome could be explained by formation of (*Z*)-enolate **E,** with PhSCH_2_ and Me occupying pseudo-equatorial positions, while the oxygen atoms are chelated to the potassium atom in axial positions [15]. The highest selectivity observed with a ketone derivative (compound **4e**, d.r. > 95:5, Scheme 2) could be explained by the presence of the additional methyl group of the ketone in an equatorial position.

## 3. Materials and Methods

### 3.1. General Considerations

Unless otherwise specified, reagents and deuterated solvents were purchased from commercial sources and used without further purification (Sigma-Aldrich^®^ (Saint-Quentin-Fallavier, France), Fisher scientific^®^ (Asnières-sur-Seine, France), TCI^®^ (Zwijndrecht, Belgium)). All the solvents were dried and freshly distilled prior to use, taking precaution to exclude moisture by refluxing over CaH_2_ or Na/benzophenone All the reactions were performed under an argon-inert atmosphere. All the glass apparatuses were oven dried and cooled under a vacuum before use. Thin layer chromatography (TLC) was performed on pre-coated sheets of silica gel 60 with a fluorescent indicator UV254 (Merck, Saint-Quentin-Fallavier, France). Detection was accomplished by irradiation with a UV lamp and by an ethanolic solution of p-anisaldehyde. Chromatographic separations were achieved under an air atmosphere using either conventional silica gel columns (Kieselgel 60, 40–63 μm, Merck) or a Puriflash Interchim^®^ 430 automatic system (Puriflash^®^ columns 50µ, Interchim, Montluçon, France), typically using cyclohexane/ethyl acetate or dichloromethane/acetone eluent systems. In all cases, distilled solvents were used as eluents for column chromatography. NMR spectra were recorded on a Bruker AvanceTM 400 spectrometer (Bruker, Wissembourg, France). The ^1^H NMR spectra were recorded at 300 MHz or 400 MHz, and the data are reported as follows: chemical shift (δ) in ppm, multiplicity (s = singlet, d = doublet, t = triplet, q = quadruplet, p = pentuplet, hept = heptuplet, b = broad, m = multiplet), coupling constants (*J*) in Hz, and their integration. The ^13^C{1H} NMR spectra were recorded at 75 MHz or 100 MHz and the data are reported as follows: chemical shift (δ) in ppm, coupling constants (*J*) in Hz, and the multiplicity of carbon atom substitution with hydrogens determined from DEPT NMR experiments. Chemical shifts are referenced relative to the signals of the deuterated solvents. High resolution mass spectra (HRMS) were measured on an Agilent (Les Ulis, France) 6530 Q-Tof MS system. The Q-TOF MS instrument was operated under the following condition: ion source ESI+ Agilent Jet Stream or APCI, both in positive ionization mode. FT-IR spectra were recorded with a Perkin Elmer Frontier, and wave numbers (ν) are quoted in cm^−1^. Melting points were determined in open capillaries on a Stuart Scientific (Staffordshire, UK) SMP 10 analyzer and are uncorrected. Infrared spectra (IR) were recorded on a Frontier Fourier transform infrared spectrophotometer instrument (Villebon-sur-Yvette, France), and are reported in wavenumbers (neat, cm^−1^). Melting points were measured on a Stuart scientific analyzer SMP10 capillary melting point apparatus and are uncorrected.

### 3.2. General Procedures

#### 3.2.1. General Procedure A for the Thia–Michael/Aldolization Route

An oven-dried glass vial equipped with a magnetic stirring bar was charged with aza–Michael product **2** (1 equiv, 0.2 mmol), PhSTMS (1.2 equiv, 0.24 mmol), and K_2_CO_3_ (1 equiv, 0.2 mmol) in THF (0.2M, 1 mL). After 2 h or 72 h at 40 °C, the solution was concentrated under a vacuum. Purification by flash chromatography on silica gel (cyclohexane/ethyl acetate, 90:10 to 70:30) was next performed to provide the title compound as separable diastereoisomers.

#### 3.2.2. General Procedure B for the Silylation/Thia–Michael/Aldol Sequence

An oven-dried glass vial equipped with a magnetic stir bar was charged with the thiophenol derivative (1.2 equiv, 0.24 mmol) and BSA (1.2 equiv, 0.04 mmol) in THF. After 30 min at 75 °C, the temperature was cooled to 40 °C. *N*-alkoxyacrylamide **2** (1 equiv, 0.2 mmol) and K_2_CO_3_ (1 equiv, 0.2 mmol) were then added. After 2 h at 6 h, the solution was concentrated in a vacuum. Purification by flash chromatography on silica gel (cyclohexane/ethyl acetate, 90:10 to 30:70) was next performed to provide the title compound as separable diastereoisomers.

#### 3.2.3. General Procedure C for the One-Pot Aza–Michael/Thia–Michael/Aldol Process

*N*-Alkoxyacrylamide **1** (1 equiv., 0.23 mmol) was added to a solution of enal (2.5 equiv, 0.56 mmol) and pyrrolidine (0.1 equiv, 0.023 mmol) in CHCl_3_ (0.2M). The solution was stirred for 24 h at room temperature. PhSTMS (1.2 equiv., 0.28 mmol) and K_2_CO_3_ (1 equiv, 0.23 mmol) were subsequently added to the aza–Michael intermediate formed. After 2 h at 40 °C, the solution was concentrated in a vacuum. Purification by flash chromatography on silica gel (cyclohexane/ethyl acetate, 90:10 to 70:30) was next performed to provide the title compound.

### 3.3. Experimental Data

*(±)-(3S,4S,6S)-1-(Benzyloxy)-4-hydroxy-6-methyl-3-((phenylthio)methyl)piperidin-2-one* (**4a**). Prepared using general procedure A from *N*-(benzyloxy)-N-(4-oxobutan-2-yl)acrylamide **2a** (0.2 mmol, 50 mg, 1 equiv). Reaction time: 2 h. Overall isolated yield: 68% (49 mg; d.r. = 88:12). Major diastereoisomer **4a_1_**—White solid. Rf = 0.52 (EtOAc/Cyclohexane 4:6). **mp**: 112 °C. IR (cm^−1^): 3425, 1960, 1640. ^1^H-NMR (400 MHz, CDCl_3_, 20 °C) δ 7.48–7.17 (m, 10H), 5.04 (d syst AB, *J* = 9.5 Hz, 1H), 4.81 (d syst AB, *J* = 9.5 Hz, 1H), 4.43 (bs, 1H), 4.06–3.95 (m, 1H), 3.85 (dd, *J* = 14.0, 4.0 Hz, 1H), 3.11 (dd, *J* = 14.0, 11.3 Hz, 1H), 2.60 (dt, *J* = 11.3, 3.5 Hz, 1H), 2.28 (d, *J* = 4.1 Hz, 1H), 2.16 (dt, *J* = 14.0, 4.3 Hz, 1H), 1.65 (t, *J* = 12.7 Hz, 1H), 1.30 (d, *J* = 6.2 Hz, 3H). ^13^C NMR (100 MHz, CDCl_3_, 20 °C) δ (ppm) 166.1, 135.7, 135.4, 129.5 (2C), 129.3 (2C), 129.1 (2C),128.7, 128.6 (2C), 126.3, 76.5, 63.8, 52.4, 47.0, 38.5, 30.1, 20.0. HRMS (ESI^+^) *m*/*z*: [M + H]^+^ calculated for C_20_H_23_NO_3_S 358.1471, found 358.1467. Minor diastereoisomer **4a_2_**—White solid. Rf = 0.36 (EtOAc/Cyclohexane 4:6). mp: 80–85 °C. ^1^H-NMR (300 MHz, CDCl_3_, 20 °C) δ 7.51–7.27 (m,), 7.23–7.18 (m, 1H_17_), 5.00 (d syst AB, *J* = 10 Hz, 1H), 4.89 (d syst AB, *J* = 10.0 Hz, 1H), 4.41 (m, 1H), 3.80 (dd, *J* = 13.8, 4.2 Hz, 1H), 3.75–3.65 (m, 1H), 3.18 (dd, *J* = 13.8, 10.7 Hz, 1H), 2.70 (dq, *J* = 10.6, 4.1 Hz, 1H), 2.01 (m, 3H), 1.45 (d, *J* = 6.6 Hz, 3H). ^13^C NMR (75 MHz, CDCl_3_, 20 °C) δ (ppm) 167.1, 135.5, 129.5 (2C), 129.3 (5C), 128.8, 128.6 (2C), 126.5, 76.4, 65.1, 54.5, 47.2, 35.7, 30.7, 20.8.
*(±)-(3S,4S,6S)-1-(Benzyloxy)-6-ethyl-4-hydroxy-3-((phenylthio)methyl)piperidin-2-one* (**4b**). Prepared using general procedure A from (*N*-(benzyloxy)-N-(1-oxopentan-3-yl)acrylamide **2b** (0.2 mmol, 53 mg, 1 equiv). Reaction time: 2 h. White solid. Overall isolated yield: 45% (33 mg; d.r. = 86:14). Rf = 0.46 (EtOAc/Cyclohexane 4:6). mp: 94 °C. IR (cm^−1^): 3397, 2932, 1724, 1656. ^1^H-NMR (400 MHz, CDCl_3_, 20 °C) 7.54–7.10 (m, 10H), 5.01 (d, *J* = 9.5 Hz, 1H), 4.84 (d, *J* = 9.6 Hz, 1H), 4.47 (m, 1H), 3.88–3.82 (m, 2H), 3.12 (dd, *J* = 14.0, 11.2 Hz, 1H), 2.59 (dt, *J* = 11.1, 3.5 Hz, 1H), 2.25–2.07 (m, 2H), 1.93 (d, *J* = 24.3 Hz, 1H), 1.63 (d, *J* = 38.3 Hz, 2H), 0.87 (t, *J* = 7.5 Hz, 3H). ^13^C NMR (100 MHz, CDCl_3_, 20 °C) δ (ppm) 168.8, 135.7, 135.4, 129.5 (2C), 129.3 (2C), 129.1 (2C), 128.7, 128.6 (2C), 126.3, 76.0, 63.8, 57.1, 47.0, 34.8, 30.2, 25.5, 8.8. HRMS (ESI+) *m*/*z*: [M + H]^+^ calculated for C_21_H_26_NO_3_S 372.1627, found 372.1628.
*(±)-(3S,4S,6S)-1-(Benzyloxy)-6-ethyl-4-hydroxy-3-((phenylthio)methyl)piperidin-2-one* (**4c**). Prepared using general procedure A from *N*-(benzyloxy)-N-(1-oxopentan-3-yl)acrylamide **2c** (0.2 mmol, 56 mg, 1 equiv). Reaction time: 2 h. White solid. Overall isolated yield: 50% (39 mg; d.r. = 83:17). Rf = 0.46 (EtOAc/Cyclohexane 4:6). mp: 90 °C. IR (cm^−1^): 3325, 2943, 2867, 1629, 1582. ^1^H-NMR (400 MHz, CDCl_3_, 20 °C) 7.55–7.14 (m, 10H), 5.02 (d, *J* = 9.6 Hz, 1H), 4.83 (d, *J* = 9.6 Hz, 1H), 4.46 (m, 1H), 3.93–3.81 (m, 2H), 3.11 (dd, *J* = 14.0, 11.2 Hz, 1H), 2.59 (dt, *J* = 11.2, 3.5 Hz, 1H), 2.20 (dt, *J* = 14.1, 4.7 Hz, 1H), 1.91 (ddt, *J* = 13.8, 5.7, 3.3 Hz, 1H), 1.83 (d, *J* = 4.5 Hz, 1H), 1.71–1.44 (m, 2H), 1.43–1.17 (m, 2H), 0.92 (t, *J* = 7.3 Hz, 3H). ^13^C NMR (100 MHz, CDCl_3_, 20 °C) δ (ppm) 168.7, 135.7, 135.4, 129.6 (2C), 129.3 (2C), 129.2 (2C), 128.8, 128.6 (2C), 126.4, 76.2, 63.9, 56.2, 47.0, 35.5, 35.1, 30.3, 17.9, 14.3. HRMS (ESI+) *m*/*z*: [M + H]^+^ calculated for C_22_H_28_NO_3_S: 373.1660, found 373.1661.
*(±)-(3S,4S,6S)-4-Hydroxy-1-methoxy-6-methyl-3-((phenylthio)methyl)piperidin-2-one* (**4d**). Prepared using general procedure A from *N*-(benzyloxy)-N-(4-oxobutan-2-yl)acrylamide **2d** (0.2 mmol, 50 mg, 1 equiv). Reaction time: 2 h. White solid. Yield: 37% (21 mg; d.r. > 95:5). Rf = 0.50 (EtOAc/Cyclohexane 4:6). mp.: 96 °C. IR (cm^−1^): 3396, 2932, 2245, 1639. ^1^H-NMR (400 MHz, CDCl_3_, 20 °C) δ 7.41–7.14 (m, 5H), 4.46–4.40 (m, 1H), 4.09–4.01 (m, 1H), 3.81 (dd, *J* = 14.1, 4.0 Hz, 1H), 3.74 (d, *J* = 1.3 Hz, 3H), 3.08 (dd, *J* = 14.0, 11.2 Hz, 1H), 2.56 (dt, *J* = 11.3, 3.3 Hz, 1H), 2.25–2.14 (m, 2H), 1.75–1.60 (m, 1H), 1.31 (d, *J* = 6.2 Hz, 3H). ^13^C NMR (100 MHz, CDCl_3_, 20 °C) δ (ppm) 169.1, 135.6, 129.3 (3C), 126.4 (2C), 63.8, 62.3, 51.9, 46.8, 38.5, 30.1, 19.9. HRMS (ESI+) *m*/*z*: [M + H]^+^ calculated for C_14_H_20_NO_3_S 282.1158, found 282.1158.
*(±)-(3S,4S,6S)-1-(Benzyloxy)-4-hydroxy-4,6-dimethyl-3-((phenylthio)methyl) piperidin-2-one* (**4e**). Prepared using general procedure A from *N*-(benzyloxy)-N-(4-oxobutan-2-yl)acrylamide **2e** (0.2 mmol, 52 mg, 1 equiv). Reaction time: 24 h. White solid. Yield: 51% (38 mg; d.r. > 95:5). Rf = 0.52 (EtOAc/Cyclohexane 4:6). mp: 109 °C. IR (cm^−1^): 3399, 2931, 2860, 1652. ^1^H NMR(400 MHz, CDCl_3_, 20 °C) δ 7.50–7.21 (m, 10H), 5.03 (d, *J* = 9.7 Hz, 1H), 4.78 (d, *J* = 9.7 Hz, 1H), 3.57 (m, 1H), 3.48–3.29 (m, 2H), 2.76 (m, 1H), 2.23 (m, 1H), 2.04 (dd, *J* = 13.5, 10.1 Hz, 1H), 1.89 (dd, *J* = 13.6, 5.4 Hz, 1H), 1.34 (d, *J* = 6.2 Hz, 3H), 1.30 (s, 3H). ^13^C NMR (100 MHz, CDCl_3_, 20 °C) δ (ppm) 168.6, 135.9, 135.3, 129.7 (2C), 129.5 (2C), 129.3 (2C), 128.9, 128.6 (2C), 126.5, 76.7, 70.0, 54.4, 53.7, 41.5, 33.3, 28.9, 20.3. HRMS (ESI+) *m*/*z*: [M + H]^+^ calculated for C_21_H_26_NO_3_S 372.1620, found 372.1628.
*(±)-(3S,4S,6S)-1-(Benzyloxy)-4-hydroxy-6-methyl-3-((o-tolylthio)methyl)piperidin-2-one* (**4f**). Prepared using general procedure B from *N*-(benzyloxy)-N-(4-oxobutan-2-yl)acrylamide **2a** (0.2 mmol, 50 mg, 1 equiv). Reaction time: 30 min + 2 h. Overall isolated yield: 63% (47 mg; d.r. = 81:19). White solid. Rf = 0.54 (EtOAc/Cyclohexane 4:6). mp: 79 °C. IR (cm^−1^): 3406, 2925, 2244, 1639. ^1^HNMR(400 MHz, CDCl_3_, 20 °C) δ 7.50–7.45 (m, 2H), 7.41–7.32 (m, 4H), 7.22–7.07 (m, 3H), 5.06 (d, *J* = 9.5 Hz, 1H), 4.82 (d, *J* = 9.5 Hz, 1H), 4.45 (m, 1H), 4.04–4.01 (m, 1H), 3.82 (dd, *J* = 13.8, 3.9 Hz, 1H), 3.11 (dd, *J* = 13.8, 11.2 Hz, 1H), 2.65 (dt, *J* = 11.2, 3.4 Hz, 1H), 2.38 (s, 3H), 2.19 (dt, *J* = 14.2, 4.5 Hz, 1H), 2.04 (d, *J* = 4.3 Hz, 1H), 1.69 (ddd, *J* = 13.7, 11.4, 1.9 Hz, 1H), 1.32 (d, *J* = 6.3 Hz, 3H). ^13^C NMR (100 MHz, CDCl_3_, 20 °C) δ (ppm) 169.0, 137.6, 135.4, 134.9, 130.5, 129.6 (2C), 128.8, 128.6 (2C), 127.7, 126.8, 126.0, 76.58, 64.05, 52.4, 46.9, 38.6, 29.3, 20.5, 20.0. HRMS (ESI+) *m*/*z*: [M + H]^+^ calculated for C_21_H_25_NO_3_S 373.1660, found 373.1661.
*(±)-(3S,4S,6S)-1-(Benzyloxy)-3-(((2,4-dimethylphenyl)thio)methyl)-4-hydroxy-6-methylpiperidin-2-one* (**4g**). Prepared using general procedure B from *N*-(benzyloxy)-N-(4-oxobutan-2-yl)acrylamide **2a** (0.2 mmol, 50 mg, 1 equiv). Reaction time: 30 min + 3 h. Overall isolated yield: 51% (46 mg; d.r. = 84:16). White solid. Rf = 0.59 (EtOAc/Cyclohexane 4:6). mp: 90 °C. IR (cm^−1^): 3391, 2988, 2923, 2288, 1980, 1886, 1722, 1638. ^1^H NMR(400 MHz, CDCl_3_, 20 °C) 7.51–6.95 (m, 8H), 5.04 (d, *J* = 9.5 Hz, 1H), 4.81 (d, *J* = 9.5 Hz, 1H), 4.45 (m, 1H), 4.06–3.97 (m, 1H), 3.76 (dd, *J* = 13.9, 4.0 Hz, 1H), 3.06 (dd, *J* = 13.9, 11.3 Hz, 1H), 2.61 (dt, *J* = 11.2, 3.5 Hz, 1H), 2.37 (s, 3H), 2.29 (s, 3H), 2.18 (m, 1H), 2.08 (d, *J* = 4.2 Hz, 1H), 1.68 (ddd, *J* = 13.7, 11.3, 1.9 Hz, 1H), 1.31 (d, *J* = 6.2 Hz, 3H). ^13^C NMR (100 MHz, CDCl_3_, 20 °C) δ (ppm) 169.1, 138.2, 136.3, 135.4, 131.5, 131.0, 129.5 (2C), 129.1, 128.7, 128.6 (2C), 127.5, 76.5, 64.0, 52.4, 47.0, 38.5, 30.0, 21.0, 20.5, 20.0. HRMS (ESI+) *m*/*z*: [M + H]^+^ calculated for C_22_H_27_NO_3_S 386.1740, found *m*/*z* 386.1780.
*(±)-(3S,4S,6S)-1-(Benzyloxy)-3-(((2,6-dimethylphenyl)thio)methyl)-4-hydroxy-6-methylpiperidin-2-one* (**4h**). Prepared using general procedure B from *N*-(benzyloxy)-N-(4-oxobutan-2-yl)acrylamide **2a** (0.2 mmol, 50 mg, 1 equiv). Reaction time: 30 min + 6 h. Overall isolated yield: 30% (46 mg; d.r. = 64:36). White solid. Rf = 0.56 (EtOAc/Cyclohexane 4:6). mp: 92 °C. IR (cm^−1^): 3403, 2985, 2922, 1978, 1734, 1638. ^1^HNMR(400 MHz, CDCl_3_, 20 °C) δ 7.49–7.04 (m, 8H), 5.02 (d, *J* = 9.5 Hz, 1H), 4.78 (d, *J* = 9.5 Hz, 1H), 4.49 (m, 1H), 4.04–3.96 (m, 1H), 3.43 (dd, *J* = 13.2, 4.1 Hz, 1H), 3.00–2.85 (m, 1H), 2.56 (s, 6H), 2.53 (m, 1H), 2.21 (dt, *J* = 14.1, 4.5 Hz, 1H), 1.97 (d, *J* = 4.2 Hz, 1H), 1.71 (ddd, *J* = 13.7, 11.3, 1.9 Hz, 1H), 1.32 (d, *J* = 6.3 Hz, 3H). ^13^C NMR (100 MHz, CDCl_3_, 20 °C) δ (ppm) 169.0, 142.9, 135.4, 133.2 (2C), 129.5 (2C), 128.7, 128.6 (2C), 128.5, 128.4 (2C), 76.6, 64.6, 52.5, 48.0, 38.7, 32.2, 22.2 (2C), 20.1. HRMS (ESI+) *m*/*z*: [M + H]^+^ calculated for C_22_H_27_NO_3_S 386.1784, found *m*/*z* 386.1784.
*(±)-(3S,4S,6S)-3-(([1,1′-Biphenyl]-4-ylthio)methyl)-1-(benzyloxy)-4-hydroxy-6-methylpiperidin-2-one* (**4i**). Prepared using general procedure B from *N*-(benzyloxy)-N-(4-oxobutan-2-yl)acrylamide **2a** (0.2 mmol, 50 mg, 1 equiv). Reaction time: 30 min + 3 h. Yield: 45% yield (87 mg; d.r. > 95:5). White solid. Rf = 0.73 (EtOAc/Cyclohexane 4:6). mp: 146 °C. IR (cm^−1^): 3378, 2960, 2935, 2162, 1963, 1632. ^1^H NMR(400 MHz, CDCl_3_, 20 °C) δ 7.61–7.29 (m, 14H), 5.06 (d, *J* = 9.5 Hz, 1H), 4.82 (d, *J* = 9.5 Hz, 1H), 4.47 (m, 1H), 4.07–3.98 (m, 1H), 3.91 (dd, *J* = 14.0, 4.0 Hz, 1H), 3.15 (dd, *J* = 14.0, 11.2 Hz, 1H), 2.66 (dt, *J* = 11.1, 3.5 Hz, 1H), 2.19 (dt, *J* = 14.1, 4.5 Hz, 1H), 2.02 (d, *J* = 4.1 Hz, 1H), 1.70 (ddd, *J* = 13.8, 11.4, 1.9 Hz, 1H), 1.32 (d, *J* = 6.3 Hz, 3H). ^13^C NMR (100 MHz, CDCl_3_, 20 °C) δ (ppm) 169.0, 140.4, 139.4, 135.4, 134.7, 129.6 (2C), 129.5 (2C), 129.0 (2C), 128.8, 128.6 (2C), 127.9 (2C), 127.6, 127.0 (2C), 76.6, 63.9, 52.4, 47.0, 38.6, 30.2, 20.0. HRMS (ESI+) *m*/*z*: [M + H]^+^ calculated for C_26_H_27_NO_3_S 434.1742, found 434.1782.
*(±)-(3S,4S,6S)-1-(Benzyloxy)-3-(((4-(tert-butyl)phenyl)thio)methyl)-4-hydroxy-6-methylpiperidin-2-one* (**4j**). Prepared using general procedure B from *N*-(benzyloxy)-N-(4-oxobutan-2-yl)acrylamide **2a** (0.2 mmol, 50 mg, 1 equiv). Reaction time: 30 min + 3 h. Yield: 51% (43 mg; d.r. > 95:5). White solid. Rf = 0.55 (EtOAc/Cyclohexane 4:6). mp: 98 °C. IR (cm^−1^): 3395, 2961, 2869, 2287, 1642, 1498. ^1^H NMR(400 MHz, CDCl_3_, 20 °C) δ 7.49–7.31 (m, 9H), 5.05 (d, *J* = 9.5 Hz, 1H), 4.81 (d, *J* = 9.5 Hz, 1H), 4.47 (m, 1H), 4.05–3.97 (m, 1H), 3.82 (dd, *J* = 14.0, 4.1 Hz, 1H), 3.08 (dd, *J* = 14.0, 11.3 Hz, 1H), 2.62 (dt, *J* = 11.3, 3.6 Hz, 1H), 2.18 (m, 1H), 1.85 (d, *J* = 4.3 Hz, 1H), 1.69 (ddd, *J* = 13.8, 11.4, 1.9 Hz, 1H), 1.34–1.27 (m, 12H). ^13^C NMR (100 MHz, CDCl_3_, 20 °C) δ (ppm) 169.1, 149.9, 135.4, 131.9, 129.6 (2C), 129.5 (2C), 128.8, 128.6 (2C), 126.4 (2C), 76.6, 63.9, 52.4, 47.0, 38.5, 34.6, 31.4, 30.7 (3C), 20.0. HRMS (ESI+) *m*/*z*: [M + H]^+^ calculated for C_24_H_31_NO_3_S 414.2097, found 414.2091.
*(±)-(3S,4S,6S)-1-(Benzyloxy)-4-hydroxy-6-methyl-3-((naphthalen-2-ylthio)methyl) piperidin-2-one* (**4k**). Prepared using general procedure B from *N*-(benzyloxy)-N-(4-oxobutan-2-yl)acrylamide **2a** (0.2 mmol, 50 mg, 1 equiv). Reaction time: 30 min + 4 h. Yield: 42% (35 mg; d.r. > 95:5). White solid. Rf = 0.68 (EtOAc/Cyclohexane 4:6). mp: 140 °C. IR (cm^−1^): 3376, 3054, 2965, 2853, 1745, 1642, 1593. ^1^H NMR(400 MHz, CDCl_3_, 20 °C) δ 7.86–7.72 (m, 4H), 7.53–7.30 (m, 8H), 5.06 (d, *J* = 9.5 Hz, 1H), 4.81 (d, *J* = 9.5 Hz, 1H), 4.43 (m, 1H), 4.06–3.89 (m, 2H), 3.20 (dd, *J* = 14.0, 11.3 Hz, 1H), 2.67 (dt, *J* = 11.3, 3.5 Hz, 1H), 2.17 ((dt, *J* = 14.2, 4.5 Hz, 1H), 1.91 (d, *J* = 4.3 Hz, 1H), 1.67 (ddd, *J* = 13.7, 11.5, 1.7 Hz, 1H), 1.31 (d, *J* = 6.3 Hz, 3H). ^13^C NMR (100 MHz, CDCl_3_, 20 °C) δ (ppm) 169.1, 135.4, 133.9, 133.0, 132.0, 129.6, 128.9, 128.8, 128.6, 127.9, 127.3, 127.1 (2C), 126.9 (3C), 126.0, 76.6, 63.9, 52.4, 46.9, 38.6, 30.0, 20.0. HRMS (ESI+) *m*/*z*: [M + H]^+^ calculated for C_24_H_25_NO_3_S 408.1628, found *m*/*z* 408.1624.
*(±)-(3S,4S,6S)-1-(Benzyloxy)-4-hydroxy-3-(((4-methoxyphenyl)thio)methyl)-6-methylpiperidin-2-one* (**4l**). Prepared using general procedure B from *N*-(benzyloxy)-N-(4-oxobutan-2-yl)acrylamide **2a** (0.2 mmol, 50 mg, 1 equiv). Reaction time: 30 min + 2 h. Yield: 41% (32 mg; d.r. > 95:5). White solid. Rf = 0.35 (EtOAc/Cyclohexane 4:6). mp: 85 °C. IR (cm^−1^): 3377, 1638, 1493, 1242, 823. ^1^H NMR(400 MHz, CDCl_3_, 20 °C) δ 7.87–7.84 (m, 2H), 7.47–7.45 (m, 2H), 7.39–7.33 (m, 5H), 5.03 (d, *J* = 9.5 Hz, 1H), 4.79 (d, *J* = 9.5 Hz, 1H), 4.47 (m, 1H), 4.04–3.96 (m, 1H), 3.80 (s, 3H), 3.72 (dd, *J* = 14.0, 4.1 Hz, 1H), 3.03 (dd, *J* = 14.1, 11.2 Hz, 1H), 2.54 (dt, *J* = 11.2, 3.6 Hz, 1H), 2.18 (dt, *J* = 14.1, 4.5 Hz, 1H), 1.90 (d, *J* = 4.3 Hz, 1H), 1.73–1.63 (m, 1H), 1.31 (d, *J* = 6.2 Hz, 3H). ^13^C NMR (100 MHz, CDCl_3_, 20 °C) δ (ppm) 169.2, 159.2, 135.4, 132.9 (2C), 129.55 (2C), 128.7, 128.6 (2C), 125.6, 115.0 (2C), 76.6, 63.8, 55.5, 52.4, 47.0, 38.5, 32.4, 20.0. HRMS (ESI+) *m*/*z*: [M + H]^+^ calculated for C_21_H_25_NO_4_S 388.1577, found 388.1575.
*(±)-(3S,4S,6S)-1-(Benzyloxy)-4-hydroxy-3-(((2-methoxyphenyl)thio)methyl)-6-methylpiperidin-2-one* (**4m**). Prepared using general procedure B from *N*-(benzyloxy)-N-(4-oxobutan-2-yl)acrylamide **2a** (0.2 mmol, 50 mg, 1 equiv). Reaction time: 30 min + 2 h. Overall isolated yield: 46% (36 mg; d.r. = 82:18). White solid. Rf = 0.37 (EtOAc/Cyclohexane 4:6). mp: 108 °C. IR (cm^−1^): 3380, 2968, 2901, 2833, 1637. ^1^HNMR(400 MHz, CDCl_3_, 20 °C) δ 7.50–7.19 (m, 7H), 6.94 (td, *J* = 7.5, 1.2 Hz, 1H), 6.87 (dd, *J* = 8.2, 1.1 Hz, 1H), 5.04 (d, *J* = 9.5 Hz, 1H), 4.82 (d, *J* = 9.5 Hz, 1H), 4.50 (m, 1H), 4.07–3.99 (m, 1H), 3.90 (s, 3H), 3.77 (dd, *J* = 13.5, 4.0 Hz, 1H), 3.07 (dd, *J* = 13.5, 11.4 Hz, 1H), 2.63 (dt, *J* = 11.4, 3.5 Hz, 1H), 2.44 (d, *J* = 3.7 Hz, 1H), 2.20 (dt, *J* = 14.1, 4.4 Hz, 1H), 1.77–1.61 (m, 1H), 1.32 (d, *J* = 6.2 Hz, 3H). ^13^C NMR (100 MHz, CDCl_3_, 20 °C) δ (ppm) 169.1, 157.8, 135.4, 130.8, 129.5 (2C), 128.7, 128.6 (2C), 128.1, 123.4, 121.5, 110.9, 76.5, 64.0, 56.0, 52.4, 46.8, 38.4, 29.5, 20.0. HRMS (ESI+) *m*/*z*: [M + H]^+^ calculated for C_21_H_25_NO_4_S 388.1577, found 388.1571.
*(±)-(3S,4S,6S)-1-(Benzyloxy)-4-hydroxy-6-methyl-3-(((4-(trifluoromethyl)phenyl)thio) methyl)piperidin-2-one* (**4n**). Prepared using general procedure B from *N*-(benzyloxy)-N-(4-oxobutan-2-yl)acrylamide **2a** (0.2 mmol, 50 mg, 1 equiv). Reaction time: 30 min + 2 h. White oil. Yield: 32% yield (28 mg; d.r. > 95:5). Rf = 0.53 (EtOAc/Cyclohexane 4:6). IR (cm^−1^): 3379, 2928, 2894, 1745, 1639, 1604, 1328. ^1^H NMR(400 MHz, CDCl_3_, 20 °C) δ 7.53–7.32 (m, 9H), 5.06 (d, *J* = 9.5 Hz, 1H), 4.82 (d, *J* = 9.5 Hz, 1H), 4.39 (m, 1H), 4.06–3.97 (m, 1H), 3.91 (dd, *J* = 14.0, 3.8 Hz, 1H), 3.17 (dd, *J* = 14.0, 11.1 Hz, 1H), 2.63 (dt, *J* = 11.0, 3.4 Hz, 1H), 2.20 (m, 1H), 2.16 (t, *J* = 4.5 Hz, 1H), 1.76–1.62 (m, 1H), 1.32 (d, *J* = 6.3 Hz, 3H) ^13^C NMR (100 MHz, CDCl_3_, 20 °C) δ (ppm) 168.8, 141.4, 135.2, 129.6 (2C), 128.8, 128.6 (2C), 127.8 (q, *J* = 33 Hz), 127.4 (2C), 126.0 (q, *J* = 4Hz, 2C), 124.2 (q, *J* = 270 Hz), 76.7, 63.9, 52.5, 46.9, 38.6, 28.8, 20.0. HRMS (ESI+) *m*/*z*: [M + H]^+^ calculated for C_21_H_22_F_3_NO_3_S 426.1345, found 426.1343.
*(±)-(3S,4S,6S)-1-(Benzyloxy)-3-(((4-chlorophenyl)thio)methyl)-4-hydroxy-6-methylpiperidin-2-one* (**4o**). Prepared using general procedure B from *N*-(benzyloxy)-N-(4-oxobutan-2-yl)acrylamide **2a** (0.2 mmol, 50 mg, 1 equiv). Reaction time: 30 min + 2 h. White solid. Yield: 55% (44 mg; d.r. > 95:5). Rf = 0.55 (EtOAc/Cyclohexane 4:6). mp: 136 °C. IR (cm^−1^): 3406, 3059, 2899, 1639, 1589, 1453. ^1^H NMR(400 MHz, CDCl_3_, 20 °C) δ 7.47 (dd, *J* = 7.4, 2.1 Hz, 2H), 7.40–7.24 (m, 7H), 5.05 (d, *J* = 9.5 Hz, 1H), 4.81 (d, *J* = 9.5 Hz, 1H), 4.42 (m, 1H), 4.05–3.97 (m, 1H), 3.82 (dd, *J* = 14.0, 4.0 Hz, 1H), 3.10 (dd, *J* = 14.0, 11.1 Hz, 1H), 2.59 (dt, *J* = 11.0, 3.5 Hz, 1H), 2.18 (dt, *J* = 14.1, 4.5 Hz, 1H), 1.90 (d, *J* = 4.5 Hz, 1H), 1.69 (ddd, *J* = 13.8, 11.4, 1.8 Hz, 1H), 1.32 (d, *J* = 6.2 Hz, 3H). ^13^C NMR (100 MHz, CDCl_3_, 20 °C) δ (ppm) 168.8, 138.0, 135.4, 135.0, 130.3, 129.6 (2C), 128.8, 128.6 (2C), 128.5, 126.7, 126.4, 76.6, 64.0, 52.4, 46.9, 38.6, 30.0, 20.0. HRMS (ESI+) *m*/*z*: [M + H]^+^ calculated for C_20_H_22_ClNO_3_S 392.1082, found 392.1081.
*(±)-(3S,4S,6S)-1-(Benzyloxy)-3-(((4-bromophenyl)thio)methyl)-4-hydroxy-6-methylpiperidin-2-one* (**4p**). Prepared using general procedure B from *N*-(benzyloxy)-N-(4-oxobutan-2-yl)acrylamide **2a** (0.2 mmol, 50 mg, 1 equiv). Reaction time: 30 min + 2 h. Yield: 55% (48 mg; d.r. = 95:5). Orange solid. Rf = 0.52 (EtOAc/Cyclohexane 4:6). mp: 145 °C. IR (cm^−1^): 3379, 2925, 2854, 2444, 1923, 1787, 1649, 1595, 1507. ^1^H NMR(400 MHz, CDCl_3_, 20 °C) δ 7.55–7.11 (m, 9H), 5.06 (d, *J* = 9.5 Hz, 1H), 4.84 (d, *J* = 9.5 Hz, 1H), 4.48 (m, 1H), 4.03 (m, 1H), 3.86 (dd, *J* = 13.7, 3.7 Hz, 1H), 3.15 (dd, *J* = 13.7, 11.3 Hz, 1H), 2.67 (dt, *J* = 11.3, 3.3 Hz, 1H), 2.20 (dt, *J* = 14.1, 4.4 Hz, 1H), 1.95 (d, *J* = 4.4 Hz, 1H), 1.71 (ddd, *J* = 13.8, 11.4, 1.9 Hz, 1H), 1.33 (d, *J* = 6.2 Hz, 3H). ^13^C NMR (100 MHz, CDCl_3_, 20 °C) δ (ppm) 168.7, 135.4, 135.3, 133.5, 129.9, 129.6 (2C), 128.8, 128.6 (2C), 128.3, 127.6, 126.8, 76.6, 64.0, 52.4, 46.7, 38.6, 29.2, 20.0. HRMS (ESI+) *m*/*z*: [M + H]^+^ calculated for C_20_H_23_BrNO_3_S 436.0571, found 436.0577.
*(±)-(3S,4S,6S)-1-(Benzyloxy)-3-(((3-chlorophenyl)thio)methyl)-4-hydroxy-6-methylpiperidin-2-one* (**4q**). Prepared using general procedure B from *N*-(benzyloxy)-N-(4-oxobutan-2-yl)acrylamide **2a** (0.2 mmol, 50 mg, 1 equiv). Reaction time: 30 min + 2 h. Overall isolated yield: 42% (33 mg; d.r. = 79:21). White solid. Rf = 0.51 (EtOAc/Cyclohexane 4:6). mp: 130 °C. IR (cm^−1^): 3377, 2967, 2927, 2050, 1639, 1578. ^1^H NMR (300 MHz, CDCl3, 20 °C) δ 7.51–7.11 (m, 9H), 5.06 (d, *J* = 9.5 Hz, 1H), 4.82 (d, *J* = 9.5 Hz, 1H), 4.42 (m, 1H), 4.05–3.97 (m, 1H), 3.84 (dd, *J* = 13.9, 4.0 Hz, 1H), 3.12 (dd, *J* = 13.9, 11.1 Hz, 1H), 2.62 (dt, *J* = 11.3, 3.5 Hz, 1H), 2.19 (dt, *J* = 14.2, 4.5 Hz, 1H), 1.84 (d, *J* = 4.3 Hz, 1H), 1.71 (dd, *J* = 14.2, 11.7 Hz, 1H), 1.33 (d, *J* = 6.2 Hz, 3H). ^13^C NMR (75 MHz, CDCl_3_, 20 °C) δ (ppm) 168.8, 138.0, 135.3, 135.0, 130.3, 129.6 (2C), 128.8, 128.6 (2C), 128.3, 126.6, 126.3, 76.6, 63.9, 52.5, 46.9, 38.6, 29.9, 20.0. HRMS (ESI+) *m*/*z*: [M + H]^+^ calculated for C_20_H_22_ClNO_3_S 392.1082, found 392.1080.
*(±)-(3S,4S,6S)-1-(Benzyloxy)-3-(((3-bromophenyl)thio)methyl)-4-hydroxy-6-methylpiperidin-2-one* (**4r**). Prepared using general procedure B from *N*-(benzyloxy)-N-(4-oxobutan-2-yl)acrylamide **2a** (0.2 mmol, 50 mg, 1 equiv). Reaction time: 30 min + 2 h. Overall isolated yield: 42% (37 mg; d.r. = 79:21). Orange solid. Rf = 0.48 (EtOAc/Cyclohexane 4:6). IR (cm^−1^): 3355, 2934, 2865, 1925, 1756, 1652. ^1^H NMR (400 MHz, CDCl_3_, 20 °C) δ 7.51–7.14 (m, 9H), 5.06 (d, *J* = 9.5 Hz, 1H), 4.82 (d, *J* = 9.5 Hz, 1H), 4.42 (m, 1H), 4.05–3.97 (m, 1H), 3.84 (dd, *J* = 13.9, 4.0 Hz, 1H), 3.12 (dd, *J* = 13.9, 11.2 Hz, 1H), 2.63 (dt, *J* = 11.0, 3.3 Hz, 1H), 2.24–2.13 (m, 1H), 1.88 (d, *J* = 4.2 Hz, 1H), 1.69 (d, *J* = 13.4 Hz, 1H), 1.33 (d, *J* = 6.2 Hz, 3H). ^13^C NMR (100 MHz, CDCl_3_, 20 °C) δ (ppm) 168.8, 138.3, 135.4, 131.3, 130.6, 129.6 (2C), 129.3, 128.8, 128.7, 128.6 (2C), 127.8, 127.1, 76.6, 64.0, 52.4, 46.9, 38.7, 30.0, 20.0. HRMS (ESI+) C_20_H_23_BrNO_3_S 436.0573, found 436.0576.
*(±)-(3S,4S,6S)-1-(Benzyloxy)-3-(((2-chlorophenyl)thio)methyl)-4-hydroxy-6-methylpiperidin-2-one* (**4s**). Prepared using general procedure B from *N*-(benzyloxy)-N-(4-oxobutan-2-yl)acrylamide **2a** (0.2 mmol, 50 mg, 1 equiv). Reaction time: 30 min + 2 h. Overall isolated yield: 54% (43 mg; d.r. = 95:5). Pink solid. Rf = 0.53 (EtOAc/Cyclohexane 4:6). mp: 126 °C. IR (cm^−1^): 3376, 2991, 2928, 2162, 1980, 1824, 1637. ^1^H NMR (400 MHz, CDCl_3_, 20 °C) δ 7.53–7.30 (m, 7H), 7.25 (td, *J* = 7.7, 1.4 Hz, 1H), 7.13 (td, *J* = 7.7, 1.5 Hz, 1H), 5.06 (d, *J* = 9.5 Hz, 1H), 4.84 (d, *J* = 9.5 Hz, 1H), 4.48 (m, 1H), 4.07–3.99 (m, 1H), 3.86 (dd, *J* = 13.7, 3.7 Hz, 1H), 3.16 (dd, *J* = 13.7, 11.3 Hz, 1H), 2.66 (dt, *J* = 11.4, 3.4 Hz, 1H), 2.20 (dt, *J* = 14.2, 4.5 Hz, 1H), 2.02 (d, *J* = 4.4 Hz, 1H), 1.71 (ddd, *J* = 13.8, 11.4, 1.9 Hz, 1H), 1.33 (d, *J* = 6.3 Hz, 3H). ^13^C NMR (100 MHz, CDCl_3_, 20 °C) δ (ppm) 168.7, 135.4, 135.2, 133.5, 129.9, 129.6 (2C), 128.8, 128.6 (2C), 128.4, 127.6, 126.8, 76.6, 64.0, 52.4, 46.5, 38.6, 29.2, 20.0. HRMS (ESI+) *m*/*z*: [M + H]^+^ calculated for C_20_H_22_ClNO_3_S 392.1082, found 392.1081.
*(±)-(3S,4S,6S)-1-(Benzyloxy)-3-(((2-bromophenyl)thio)methyl)-4-hydroxy-6-methylpiperidin-2-one* (**4t**). Prepared using general procedure B from *N*-(benzyloxy)-N-(4-oxobutan-2-yl)acrylamide **2a** (0.2 mmol, 50 mg, 1 equiv). Reaction time: 30 min + 2 h. Overall isolated yield: 54% (47 mg; d.r. = 89:11). White solid. Rf = 0.52 (EtOAc/Cyclohexane 4:6). mp: 143 °C. IR (cm^−1^): 3377, 2961, 2869, 2287, 1641, 1498. ^1^H NMR (400 MHz, CDCl_3_, 20 °C) δ 7.53–7.30 (m, 7H), 7.25 (td, *J* = 7.7, 1.4 Hz, 1H), 7.13 (td, *J* = 7.7, 1.5 Hz, 1H), 5.06 (d, *J* = 9.5 Hz, 1H), 4.84 (d, *J* = 9.5 Hz, 1H), 4.48 (m, 1H), 4.08–3.99 (m, 1H), 3.86 (dd, *J* = 13.7, 3.7 Hz, 1H), 3.16 (dd, *J* = 13.7, 11.3 Hz, 1H), 2.66 (dt, *J* = 11.4, 3.4 Hz, 1H), 2.20 (dt, *J* = 14.2, 4.5 Hz, 1H), 2.02 (d, *J* = 4.4 Hz, 1H), 1.71 (ddd, *J* = 13.8, 11.4, 1.9 Hz, 1H), 1.33 (d, *J* = 6.3 Hz, 3H). ^13^C NMR (100 MHz, CDCl_3_, 20 °C) δ (ppm) 168.7, 137.3, 135.3, 133.2, 129.6 (2C), 128.8, 128.6 (2C), 128.2, 128.1, 126.9, 123.5, 76.6, 64.0, 52.4, 46.7, 38.6, 29.6, 20.0. HRMS (ESI+) *m*/*z*: [M + H]^+^ calculated for C_20_H_22_BrNO_3_S 436.0573, found 436.0579.
*(±)-(3S,4S,6S)-3-(((4-Aminophenyl)thio)methyl)-1-(benzyloxy)-4-hydroxy-6-methylpiperidin-2-one* (**4u**). Prepared using general procedure B from *N*-(benzyloxy)-N-(4-oxobutan-2-yl)acrylamide **3a** (0.2 mmol, 50 mg, 1 equiv). Reaction time: 30 min + 2 h. Overall isolated yield: 55% (41 mg; d.r. = 91:9). Yellow solid. Rf = 0.15 (EtOAc/Cyclohexane 4:6). mp: 48 °C. IR (cm^−1^): 3340, 2937, 2244, 1626, 1615. ^1^H NMR (400 MHz, CDCl_3_, 20 °C) δ 7.46–7.21 (m, 7H), 7.07 (td, *J* = 7.7, 1.6 Hz, 1H), 5.01 (d, *J* = 9.5 Hz, 1H), 4.78 (d, *J* = 9.5 Hz, 1H), 4.47 (m, 1H), 4.03–3.94 (m, 1H), 3.78 (s, 2H), 3.65 (dd, *J* = 14.0, 4.1 Hz, 1H), 2.97 (dd, *J* = 14.0, 11.2 Hz, 1H), 2.58–2.48 (m, 1H), 2.16 (dt, *J* = 14.1, 4.4 Hz, 1H), 1.77 (bs, 1H), 1.65 (ddd, *J* = 13.8, 11.4, 2.0 Hz, 1H), 1.30 (d, *J* = 6.2 Hz, 3H). ^13^C NMR (100 MHz, CDCl_3_, 20 °C) δ (ppm) 169.3, 147.1, 135.3, 133.9, 129.4 (2C), 128.6, 128.5 (2C), 125.7 (2C), 115.4 (2C), 76.5, 63.6, 52.3, 46.9, 38.4, 32.8, 19.9. HRMS (ESI^+^) *m*/*z*: [M + H]^+^ calculated for C_20_H_24_N_2_O_3_S 373.1573, found 373.1569.
*(±)-(3S,4S,6S)-3-(((2-Aminophenyl)thio)methyl)-1-(benzyloxy)-4-hydroxy-6-methylpiperidin-2-one* (**4v**). Prepared using general procedure B from *N*-(benzyloxy)-N-(4-oxobutan-2-yl)acrylamide **2a** (0.2 mmol, 50 mg, 1 equiv). Reaction time: 30 min + 2 h. Overall isolated yield: 49% (37 mg; d.r. = 84:16). Yellow solid. Rf = 0.18 (EtOAc/Cyclohexane 4:6). mp: 51 °C. IR (cm^−1^): 3346, 2928, 2245, 1635, 1606. ^1^H NMR (400 MHz, CDCl_3_, 20 °C) δ 7.46–7.16 (m, 6H), 7.07 (td, *J* = 7.7, 1.6 Hz, 1H), 6.71–6.60 (m, 2H), 5.02 (d, *J* = 9.5 Hz, 1H), 4.78 (d, *J* = 9.6 Hz, 1H), 4.48 (m, 1H), 4.38 (s, 2H), 4.03–3.94 (m, 1H), 3.64 (dd, *J* = 13.7, 4.3 Hz, 1H), 2.94 (dd, *J* = 13.7, 10.7 Hz, 1H), 2.55 (dt, *J* = 10.6, 3.5 Hz, 1H), 2.18 (dt, *J* = 14.1, 4.5 Hz, 1H), 2.00 (bs, 1H), 1.70 (ddd, *J* = 13.8, 11.4, 1.9 Hz, 1H), 1.31 (d, *J* = 6.1 Hz, 3H). ^13^C NMR (100 MHz, CDCl_3_, 20 °C) δ (ppm) 169.4, 148.2, 135.5 (2C), 130.0, 129.5 (2C), 128.7, 128.6 (2C), 118.9, 117.3, 115.5, 76.6, 64.2, 52.5, 47.6, 38.5, 31.4, 20.0. HRMS (ESI+) *m*/*z*: [M + H]^+^ calculated for C_20_H_24_N_2_O_3_S 373.1573, found *m*/*z* 373.1580.
*(±)-(3S,4S,6S)-1-(Benzyloxy)-3-((benzylthio)methyl)-4-hydroxy-6-methylpiperidin-2-one* (**4w**). Prepared using general procedure B from *N*-(benzyloxy)-N-(4-oxobutan-2-yl)acrylamide **2a** (0.2 mmol, 50 mg, 1 equiv). Reaction time: 30 min + 3 h. White solid. Yield: 11% (8 mg; d.r. > 95:5). Rf = 0.63 (EtOAc/Cyclohexane 4:6). mp: 84 °C IR (cm^−1^): 3405, 2988, 2923, 2288, 1980, 1886, 1722, 1638, 1495. ^1^H NMR (400 MHz, CDCl_3_, 20 °C) δ 7.49–7.03 (m, 10H), 4.94 (d, *J* = 9.5 Hz, 1H), 4.72 (d, *J* = 9.5 Hz, 1H), 4.20 (m, 1H), 3.93–3.84 (m, 1H), 3.68 (s, 2H), 3.19 (dd, *J* = 13.3, 4.3 Hz, 1H), 2.66 (dd, *J* = 13.2, 10.9 Hz, 1H), 2.42 (dt, *J* = 10.7, 3.5 Hz, 1H), 2.07 (dt, *J* = 14.0, 4.4 Hz, 1H), 1.93 (m, 1H), 1.54–1.51 (m, 1H), 1.22 (d, *J* = 6.2 Hz, 3H). ^13^C NMR (100 MHz, CDCl_3_, 20 °C) δ (ppm) 169.1, 138.5, 135.4, 129.5 (2C), 129.0 (2C), 128.8 (2C), 128.7, 128.6 (2C), 127.4, 76.5, 64.4, 52.5, 47.1, 38.4, 37.3, 28.8, 20.0. HRMS (ESI+) *m*/*z*: [M + H]^+^ calculated for C_21_H_25_NO_3_S 372.1628, found 372.1626.
*(±)-(3S,4S,6S)-1-(Benzyloxy)-4-hydroxy-6-methyl-3-((propylthio)methyl)piperidin-2-one* (**4x**). Prepared using general procedure B from *N*-(benzyloxy)-N-(4-oxobutan-2-yl)acrylamide **2a** (0.2 mmol, 50 mg, 1 equiv). Reaction time: 30 min + 3 h. White solid. Yield: 15% (10 mg; d.r. > 95:5). Rf = 0.51 (EtOAc/Cyclohexane 4:6). mp: 75 °C. IR (cm^−1^): 3398, 2966, 2935, 2901, 2876, 1815, 1650, 1636. ^1^H NMR (400 MHz, CDCl_3_, 20 °C) δ 7.49–7.46 (m, 2H), 7.38–7.33 (m,3H), 5.04 (d, *J* = 9.5 Hz, 1H), 4.82 (d, *J* = 9.5 Hz, 1H), 4.39 (m, 1H), 4.07–3.98 (m, 1H), 3.25 (dd, *J* = 13.3, 4.4 Hz, 1H), 2.86 (dd, *J* = 13.2, 10.7 Hz, 1H), 2.64 (dq, *J* = 10.7, 4.1, 1H), 2.58–2.48 (m, 2H), 2.31 (d, *J* = 3.7 Hz, 1H), 2.21 (dt, *J* = 14.1, 4.5 Hz, 1H), 1.81–1.54 (m, 3H), 1.34 (d, *J* = 6.3 Hz, 3H), 0.99 (t, *J* = 7.3 Hz, 3H). ^13^C NMR (100 MHz, CDCl_3_, 20 °C) δ (ppm) 169.1, 135.4, 129.6 (2C), 128.7, 128.6 (2C), 76.6, 64.5, 52.5, 47.2, 38.6, 34.9, 29.2, 23.0, 20.1, 13.6. HRMS (ESI+) *m*/*z*: [M + H]^+^ calculated for C_17_H_25_NO_3_S 324.1628, found 324.1620.

## 4. Conclusions

We have developed an original two-component thia–Michael/aldol process between aza–Michael adducts of acrylamides and α,β-unsaturated carbonyl compounds, diastereoselectively affording novel types of polysubstituted lactams. This domino reaction features mild conditions, good functional group tolerance, moderate-to-good yields, and generally good-to-high stereoselectivity for these lactam targets. The procedure was extended to a sequential one-pot aza–Michael/thia–Michael/aldol sequence demonstrating for the first time that acrylamides can directly and easily be used as both 1,3-bis-nucleophiles and 1,4-electrophiles in a single flask. The application of this methodology for the synthesis of more complex heterocyclic structures of potential bioactivity is currently underway in our laboratory and will be published in due course.

## Figures and Tables

**Table 1:** The optimization of the reaction conditions using PhS-TMS as the nucleophile ^1^.

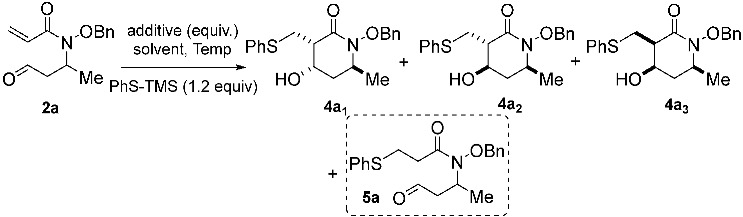						
Entry	Additive (equiv.)	Solvent	T °C	Overall Yield 4a (%) ^2^	Ratio ^3^ 4a_1_:4a_2_:4a_3_	Yield 5a (%) ^2^
1	-	CHCl_3_	20	0	-	8
2	TBAF (0.2)	CHCl_3_	20	55	44:0:56	11
3	TBAT (0.2)	CHCl_3_	20	0	-	8
4	^t^BuOK (0.2)	CHCl_3_	20	45	84:16:0	2
5	Na_2_CO_3_ (0.2)	CHCl_3_	20	0	-	-
6	Cs_2_CO_3_ (0.2)	CHCl_3_	20	29	93:7:0	2
7	K_2_CO_3_ (0.2)	CHCl_3_	20	37	95:0:5	3
8	K_2_CO_3_ (0.2)	CHCl_3_	40	46	87:0:13	10
9	K_2_CO_3_ (1.0)	CHCl_3_	40	62	87:13:0	5
10	K_2_CO_3_ (1.0)	CH_2_Cl_2_	40	n.d. ^4^	-	n.o. ^5^
11	K_2_CO_3_ (1.0)	DCE	40	n.d. ^4^	-	n.o. ^5^
12	K_2_CO_3_ (1.0)	MeCN	40	24	100:0:0	20
13	K_2_CO_3_ (1.0)	toluene	40	36	100:0:0	6
14	K_2_CO_3_ (1.0)	DMF	40	44	67:11:22	6
15	K_2_CO_3_ (1.0)	Et_2_O	40	44	77:23:0	6
16	K_2_CO_3_ (1.0)	THF	40	68	88:12:0	8

^1^ Reactions were carried out with alkoxyacrylamide **2a** (0.28 mmol, 1 equiv.), PhSTMS **3a** (1.2 equiv.), and an additive in 1.4 mL of solvent. ^2^ Overall isolated yield of **4a**. ^3^ d.r.: the diastereomeric ratio determined from the isolated yields. ^4^ n.d.: not determined. ^5^ n.o.: not observed.

## Data Availability

The data are contained within the article or the Supplementary Materials.

## Supplementary Materials

The following supporting information can be downloaded at https://www.mdpi.com/article/10.3390/molecules30102154/s1. The crystallographic data for compound **4a_1_** (CCDC 2396647) is available free of charge from the Cambridge Crystallographic Data Center at https://www.ccdc.cam.ac.uk/structures (accessed on 11 April 2024). Refs. [14,17] are cited in the Supplementary Materials.

## References

[1] Ferro C.T.B., Santos B.F., Silva C.D.G., Brand G., Silva B.A.L., Domingues N.L.C. (2020). Review of the Syntheses and Activities of Some Sulfur-Containing Drugs. Curr. Org. Synth..

[2] Ahangarpour M., Kavianinia I., Brimble M.A. (2023). Thia-Michael addition: The route to promising opportunities for fast and cysteine-specific modification. Org. Biomol. Chem..

[3] Berne D., Ladmiral V., Leclerc E., Caillol S. (2022). Thia-Michael Reaction: The Route to Promising Covalent Adaptable Networks. Polymers.

[4] Wadhwa P., Kharbanda A., Sharma A. (2018). Thia-Michael Addition: An Emerging Strategy in Organic Synthesis. Asian J. Org. Chem..

[5] Niu C., Du D.-M. (2023). Recent Advances in Organocatalyzed Asymmetric Sulfa-Michael Addition Triggered Cascade Reactions. Chem. Rec..

[6] Wang X., Luo Y., Zhao J., Luo S. (2023). CPA-catalyzed asymmetric domino thia-Michael/aldol reactions for simultaneous chiral center and axial chirality formation. Org. Biomol. Chem..

[7] Alahyen I., Benhamou L., Dalla V., Taillier C., Comesse S. (2021). 20 Years of Forging N-Heterocycles from Acrylamides Through Domino/Cascade Reactions. Synthesis.

[8] Kamimura A., Omata Y., Mitsudera H., Kakehi A. (2000). A simple preparation of syn-NH-amide aldols and amide-Baylis-Hillman adducts via a Michael-aldol tandem process. J. Chem. Soc. Perkin Trans. 1.

[9] Zu L., Wang J., Li H., Xie H., Jiang W., Wang W. (2007). Cascade Michael-Aldol Reactions Promoted by Hydrogen Bonding Mediated Catalysis. J. Am. Chem. Soc..

[10] Zhang Y.-P., You Y., Zhao J.-Q., Zhang X.-M., Xu X.-Y., Yuan W.-C. (2019). Chiral Bifunctional Amine-Squaramide-Catalyzed Highly Diastereoand Enantioselective Michael/Aldol Cascade Reaction of 2-Mercaptobenzaldehyde and α,β-Unsaturated 7-Azaindoline Amides. J. Org. Chem..

[11] Arai T., Miyazaki T., Ogawa H., Masu H. (2016). PyBidine-Ni(OAc)_2_-Catalyzed Michael/Aldol Reaction of Methyleneindolinones and Thiosalicylaldehydes for Stereochemically Divergent Thiochromanyl-spirooxindoles. Org. Lett..

[12] Hao Y., Li Z.-H., Lian P.-F., Li Q.-Z., She Y., Ma Z.-G., Zhang S.-Y. (2023). Stereoselective Sulfa-Michael/Aldol Reaction Promoted by an Axially Chiral Styrene-Based Organocatalyst. Org. Lett..

[13] Champetter P., Castillo-Aguilera O., Taillier C., Brière J.-F., Dalla V., Oudeyer S., Comesse S. (2019). *N*-Alkoxyacrylamides in Domino Reactions: Catalytic and Stereoselective Access to δ-Lactams. Eur. J. Org. Chem..

[14] Alahyen I., Taillier C., Lhoste J., Dalla V., Comesse S. (2024). *N*-Benzyloxyacrylamides as bis-Nucleophiles in an Organocatalyzed Domino aza-Michael/Morita-Baylis-Hillman Sequence. Org. Lett..

[15] Champetter P., Alahyen I., Taillier C., Brière J.-F., Dalla V., Oudeyer S., Comesse S. (2022). Probing *N*-Alkoxy Effects in Domino Reactions of α-Bromoacetamide Derivatives Towards Functionalized γ-Lactams. ChemistrySelect.

[16] Khodair A.I., Al-Masoudi N.A., Gesson J.-P. (2003). A new approach to the synthesis of benzothiazole, benzoxazole, and pyridine nucleosides as potential antitumor agents. Nucleosides Nucleotides Nucleic Acids.

[17] Sheldrick G.M. (2014). “SHELXL-2014”, Program for Crystal Structure Determination.

