# Supplementary material for: Straightforward Access to Polyfunctionalized δ-Lactams via Domino Aza–Michael/Thia–Michael/Aldol Sequence
Source: Molecules. 2025 May 14;30(10):2154. doi: 10.3390/molecules30102154 (PMC12114351; doi:10.3390/molecules30102154)
Supplement: Supplementary file 1 [file molecules-30-02154-s001.zip › molecules-3604257-supplementary.pdf]

# Straightforward Access to Polyfunctionalized δ-Lactams via an Aza-Michael/Thia-Michael/Aldol Sequence

**Authors:** Axelle Genty [1], Ismail Alahyen [1], Marie-José Tranchant, Jérôme Lhoste [2], Vincent Dalla [1], Catherine Taillier [1,*] and Sébastien Comesse [1*]

**Affiliations:**

[1] Université Le Havre Normandie, Normandie Univ., URCOM UR 3221, F-76600 Le Havre, France.

[2] IMMM, UMR 6283 CNRS, Le Mans Université, 72085 Le Mans, France.

*Corresponding authors. Emails: catherine.taillier@univ-lehavre.fr, sebastien.comesse@univ-lehavre.fr

# Table of Contents

Optimization of the thia-Michael/aldol domino sequence: complementary experiments…..................... 2

Crystallographic data collection and structure determination of compound **4a$_1$**…................................. 4

Stereochemistry determination of compounds **4a$_2$** and **4a$_3$**……………………………………..…….. 6

$^1$H and 13C{1H} NMR of compounds **4a-4z**........................................................................................ 8

# Optimization of the thia-Michael/aldol domino sequence: complementary experiments

We started our study with acrylamide **2a** and thiophenol as model substrates (Table S1). No reaction occurred in the absence of additive in $CHCl_3$ (entry 1). Total decomposition was observed upon addition of a strong base (entry 2). When running the reaction at higher temperature or with various amounts of $Et_3N$, yields in cyclic product **4a** were increased but lower d.r. were obtained probably due to some post-epimerization reactions (compare entries 3 to 5, Table 1). In each case, high amounts of the uncyclized compound **5a** were isolated. The use of a carbonated base, *i.e.* $K_2CO_3$, at 20 or 40°C in $CHCl_3$ gave acceptable yields and selectivities but still with important quantities of the undesired product **5a** (entries 7-8).

Considering the competitive protonation of the intermediate enolate formed after the thia-Michael addition leading to the undesired amide **5a**, we decided to turn our attention toward the commercially available PhS-TMS as nucleophile in order to limit the presence of acidic protons in the media. Surprisingly, in the absence of additive only the formation of small amounts of the uncyclized compound **5a** was observed (entry 10). Thankfully, in the presence of TBAF acceptable yields of the desired product **4a** were obtained albeit with low d.r. (entry 11). The use of other solvents or other fluorinated agent was detrimental to the yields (entries 12-19). We shifted then to other promoting agents bearing a nucleophilic oxygen atom such as *t*-BuOK and even if the desired product was isolated in moderate yields, the diastereoselectivity remained low (entries 20-21). $K_2CO_3$ proved to be a good compromise as it was reactive enough to promote the desilation of the nucleophile but not too basic to favor side reactions such as epimerization. After screening of different solvents and temperatures (entries 22-33), the best conditions allowing the higher yields and acceptable d.r. were selected, *i.e.* C(**2a**) = 0.2 M in THF, PhS-TMS (1.2 equiv.), $K_2CO_3$ (1 equiv.) at 40°C (entry 32).

**Table S1. Optimisation of the reaction conditions**[a]

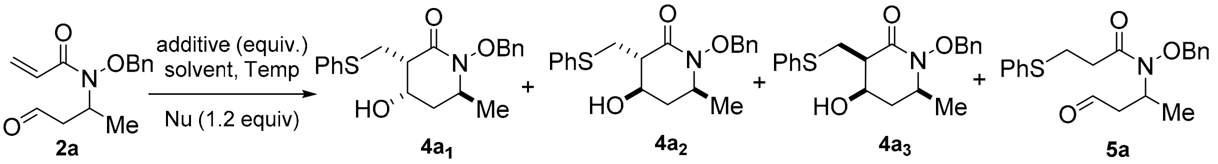


| Entry | Nu | Additive (equiv.) | Solvent | T (°C) | Yield **4a** (%)[b] | Ratio[c] **4a1:4a2:4a3c** | Yield **5a** (%)[b] |
|---|---|---|---|---|---|---|---|
| 1 | PhSH | - | $CHCl_3$ | 20 | nr[d] | | |
| 2 | PhSH | NaH (0.2) | $CH_2Cl_2$ | -15 to 20 | dec[e] | | |
| 3 | PhSH | TEA (0.2) | $CHCl_3$ | 20 | 11 | 100:0:0 | 51 |
| 4 | PhSH | TEA (0.2) | $CHCl_3$ | 40 | 31 | 68:26:6 | 47 |
| 5 | PhSH | TEA (1) | $CHCl_3$ | 20 | 28 | 64:25:11 | 51 |
| 7 | PhSH | $K_2CO_3$ (0.2) | $CHCl_3$ | 20 | 25 | 100:0:0 | 23 |
| 8 | PhSH | $K_2CO_3$ (0.2) | $CHCl_3$ | 40 | 50 | 90:10:0 | 22 |
| 9 | PhSH | $K_2CO_3$ (1) | $CHCl_3$ | 20 | 54 | 85:15:0 | 37 |
| 10 | PhS-TMS | - | $CHCl_3$ | 20 | 0 | - | 8 |
| 11 | PhS-TMS | TBAF (0.2) | $CHCl_3$ | 20 | 55 | 44:0:56 | 11 |

| | | | | | | | |
|---|---|---|---|---|---|---|---|
| 12 | PhS-TMS | TBAF (0.2) | $CH_2Cl_2$ | 20 | 3 | 100:0:0 | 25 |
| 13 | PhS-TMS | TBAF (0.2) | THF | 20 | 18 | 61:0:39 | 15 |
| 14 | PhS-TMS | TBAF (0.2) | DMF | 20 | 49 | 27:0:73 | 3 |
| 15 | PhS-TMS | TBAF (0.2) | EtOH | 20 | 7 | 0:0:100 | 55 |
| 16 | PhS-TMS | TBAF (0.2) | $^t$BuOH | 20 | 22 | 27:18:55 | 2 |
| 17 | PhS-TMS | TBAF (0.2) | toluene | 20 | 30 | 70:0:30 | 18 |
| 18 | PhS-TMS | TBAF (0.2) | dioxane | 20 | 22 | 73:0:27 | 15 |
| 19 | PhS-TMS | TBAT (0.2) | $CHCl_3$ | 20 | 0 | - | 8 |
| 20 | PhS-TMS | $^t$BuOK (0.2) | $CHCl_3$ | 20 | 45 | 84:16:0 | 2 |
| 21 | PhS-TMS | $^t$BuOK (0.2) | THF | 20 | 19 | 53:32:15 | - |
| 22 | PhS-TMS | $Na_2CO_3$ (0.2) | $CHCl_3$ | 20 | 0 | - | - |
| 23 | PhS-TMS | $Cs_2CO_3$ (0.2) | $CHCl_3$ | 20 | 29 | 93:7:0 | 2 |
| 24 | PhS-TMS | $K_2CO_3$ (0.2) | $CHCl_3$ | 20 | 37 | 95:0:5 | 3 |
| 25 | PhS-TMS | $K_2CO_3$ (1.0) | $CHCl_3$ | 20 | 32 | 93:0:7 | 3 |
| 26 | PhS-TMS | $K_2CO_3$ (1.0) | $CHCl_3$ | 40 | 62 | 87:13:0 | 5 |
| 27 | PhS-TMS | $K_2CO_3$ (1.0) | $CH_2Cl_2$ | 40 | n.d.[f] | - | n.o.[g] |
| 28 | PhS-TMS | $K_2CO_3$ (1.0) | DCE | 40 | n.d.[f] | - | n.o.[g] |
| 29 | PhS-TMS | $K_2CO_3$ (1.0) | MeCN | 40 | 24 | 100:0:0 | 20 |
| 30 | PhS-TMS | $K_2CO_3$ (1.0) | toluene | 40 | 36 | 100:0:0 | 6 |
| 31 | PhS-TMS | $K_2CO_3$ (1.0) | DMF | 40 | 44 | 67:11:22 | 6 |
| 32 | PhS-TMS | $K_2CO_3$ (1.0) | THF | 40 | 68 | 88:12:0 | 8 |
| 33 | PhS-TMS | $K_2CO_3$ (1.0) | $Et_2O$ | 40 | 44 | 77:23:0 | 6 |

[a]Reactions were carried out with acrylamide **2a** (0.28 mmol, 1 equiv.), PhSH or PhSTMS (1.2 equiv.) and additive in 1.4 mL of solvent. [b]Isolated yield of **4a**. [c]dr: diastereomeric ratio determined on the crude mixture. [d]nr: no reaction. [e]dec: decomposition. [f]n.d.: not determined. [g]n.o.: not observed.

Considering the competitive protonation of the intermediate enolate formed after the thia-Michael addition leading to the undesired amide **5a**, we decided to turn our attention toward the commercially available PhS-TMS as nucleophile in order to limit the presence of acidic protons in the media. Surprisingly, in the absence of additive only the formation of small amounts of the uncyclized compound **5a** was observed (entry 10). Thankfully, in the presence of TBAF acceptable yields of the desired product **4a** were obtained albeit with low d.r. (entry 11). The use of other solvents or other fluorinated agent was detrimental to the yields (entries 12-19). We shifted then to other promoting agents bearing a nucleophilic oxygen atom such as *t*-BuOK and even if the desired product was isolated in moderate yields, the diastereoselectivity remained low (entries 20-21). $K_2CO_3$ proved to be a good compromise as it was reactive enough to promote the desilation of the nucleophile but not too basic to favor side reactions such as epimerization. After screening of different solvents and temperatures (entries 22-33), the best conditions allowing the higher yields and acceptable d.r. were selected, *i.e.* C(**2a**) = 0.2 M in THF, PhS-TMS (1.2 equiv.), $K_2CO_3$ (1 equiv.) at 40°C (entry 32).

# Crystallographic data collection and structure determination of compound 4a$_1$

### *X-ray Crystal Structure Determination*

Crystals were selected under polarizing optical microscope and mounted on MicroMount needles (MiTiGen) for single-crystal X-ray diffraction experiments. X-ray intensity data were collected on a Bruker APEX II Quazar diffractometer (4 circle Kappa goniometer, CCD detector) using Ims microfocus source (Mo-$K_\alpha$ radiation with $\lambda$ = 0.71073 Å) at 296 K. The structure solutions were obtained by direct methods, developed by successive difference Fourier syntheses, and refined by full-matrix least-squares on all $F^2$ data using SHELX program suite[1] in Bruker APEX2 interface. Details of the structure determinations are given in Table 1.

Crystallographic data (excluding structure factors) have been deposited at the Cambridge Crystallographic Data Centre, CCDC **n°**2396647 ($C_{20}H_{23}NO_3S$). Copies of the data can be obtained, free of charge, on application to CCDC, 12 Union Road, Cambridge CB2 1EZ, UK, (fax: +44 1223 336033 or e-mail: deposit@ccdc.cam.ac.uk).

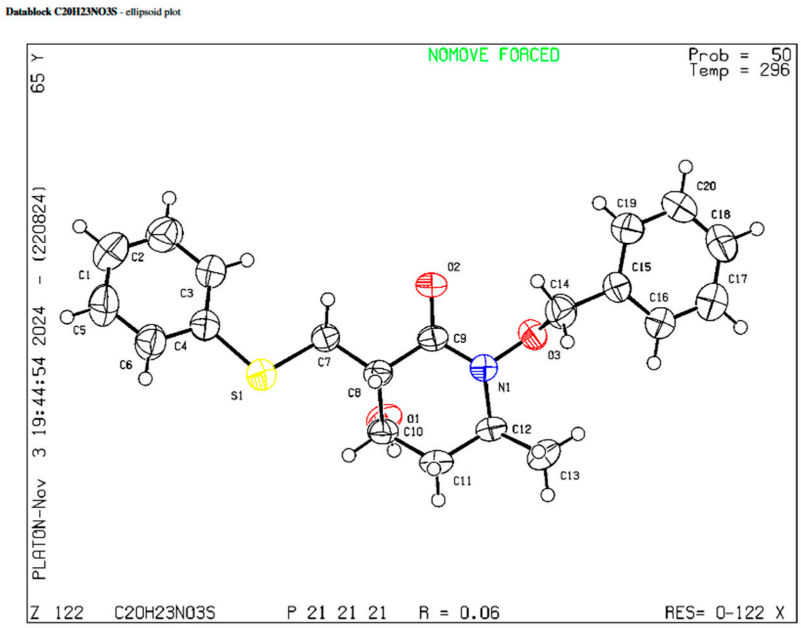


***Figure S1. Molecular structure of compound 4a1***

---

1 Sheldrick, G.M. " SHELXL-2014 ", Program for crystal structure determination, Göttingen Univ., Germany (2014).

***Table S2. Crystal data and structure refinement for compound 4a1***

| | |
|---|---|
| Identification code | $C_{20}H_{23}NO_3S$ |
| Formula mass (g.mol$^{-1}$) | 357.45 |
| *Unit cell dimensions*<br>a (Å) =<br>b (Å) =<br>c (Å) = | <br>4.9520(7)<br>12.0372(12)<br>31.375(3) |
| Volume, Z | 1870.2(4), 4 |
| Calculated density (g.cm$^{-3}$) | 1.270 |
| Crystal system,<br>Space group | Orthorhombic,<br>$P2_12_12_1$ |
| 2q range for data collection (°) | 5.2-55.2 |
| Temperature (K), scan method | 296, 2q/w |
| $(hkl)_{min}$<br>$(hkl)_{max}$ | (-5 -15 -40)<br>(6 15 40) |
| coefficient (mm$^{-1}$) | 0.191 |
| Reflections (I>2s(I)), refined numbers | 2217, 232 |
| Goodness of fit (F$^2$) | 1.017 |
| $R_1$ [I >2s(I)], $wR_2$ (all data) | 0.0579 / 0.1319 |

# Stereochemistry determination of compounds $4a_2$ and $4a_3$

We have recently described a domino aza-Michael/Morita-Baylis-Hillman (MBH) sequence from alkoxyacrylamides and enals, giving access to δ-lactams such as **6a**.[2]

In this study, we have previously demonstrated that the major diastereomer **4a₁** obtained, presents a syn relationship between the hydroxy group and the alkyl chain (see the NOE effect between the protons $H_2$ and $H_4$ in Figure S2 below). The stereochemistry of the compound **6a** was thus determined to be (±)-(4*R*,6*S*).

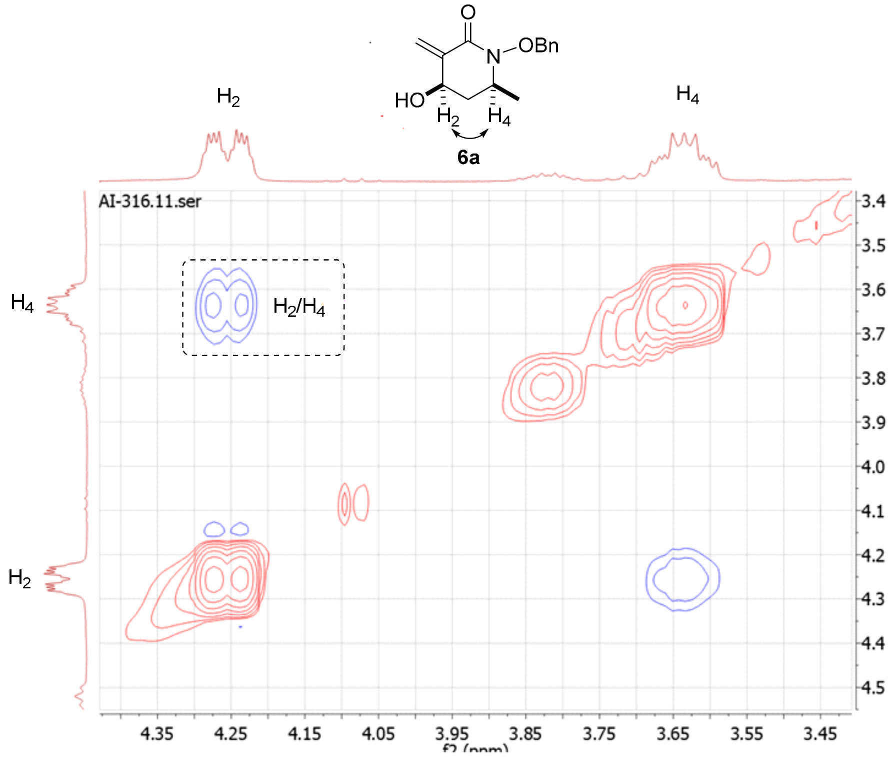


***Figure S2. Assignment of the stereochemistry of the major diastereomer 6a by NOESY experiment***

A thia-Michael reaction was next performed on the aza-Michael/MBH major diastereomer **6a** using thiophenol as nucleophile, under basic conditions [$Et_3N$ (0.2 equiv.), $CH_2Cl_2$, r.t.] at room temperature). This resulted in the formation of the cyclic adduct **4a** in 94% overall yield as a mixture of three diastereomers **$4a_1$**,**$4a_2$** and **$4a_3$** (d.r. = 4 :34 :62, Scheme S1).

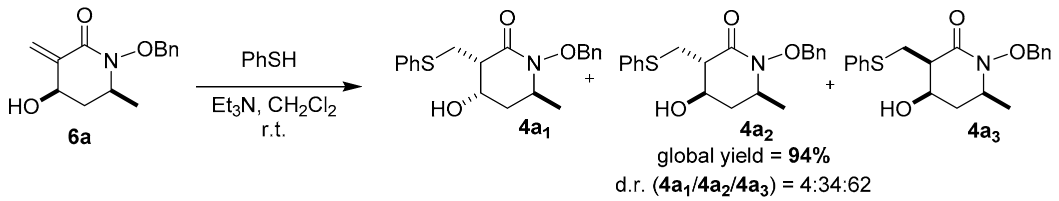


***Scheme S1. Thia-Michael reaction on the aza-Michael/MBH major diastereomer 6a***

[2] Alahyen, I. ; Taillier, C. ; Lhoste, J. ; Dalla, V. ; Comesse, S. *Org. Lett.* **2024**, *26*, 1926-1930. Full characterization of diastereoisomers **$4a_2$** and **$4a_3$** is given.

After isolation of each diastereomer, **NOESY NMR** experiment was carried out on the diastereomer **4a$_3$**. An interaction in space between the protons H$_2$ and H$_1$ α was observed, as well as an interaction between the protons H$_2$ and H$_4$, thus confirming a syn relationship between the hydroxy group, the methyl and the chain bearing the thiophenyl group (Figure S3). The diastereomer **4a$_3$** was therefore assigned to have the stereochemistry (±)-(3*R*,4*R*,6*S*).

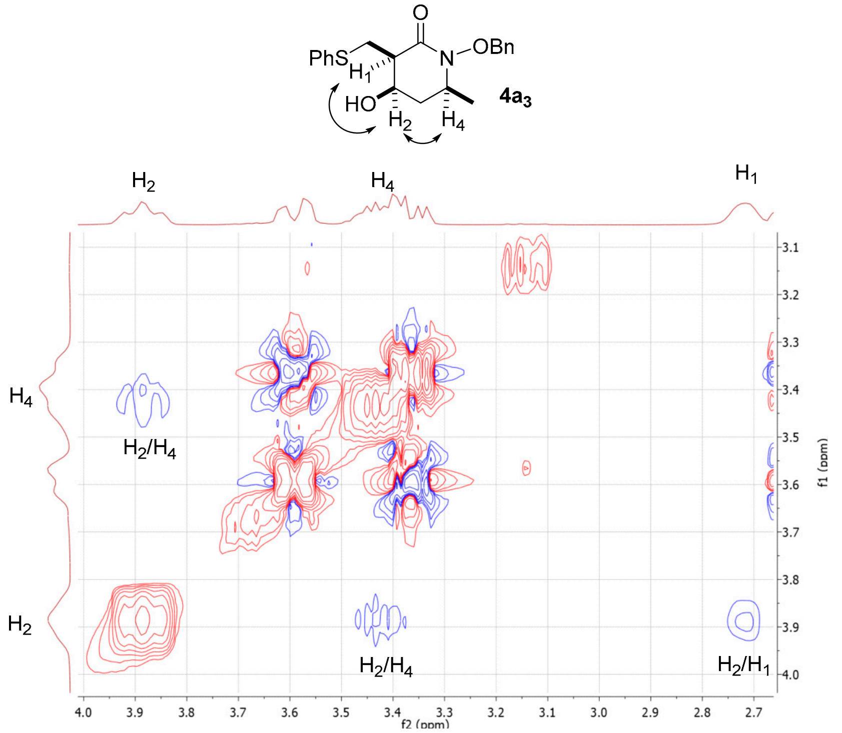


***Figure S3. Assignment of the stereochemistry of the diastereomer 4a$_3$ by NOESY experiment***

Having identified the diastereomers **4a$_1$** and **4a$_3$**, the stereochemistry of the diastereomer **4a$_2$** resulting from the same diastereomer of the substrate **6a** can be deduced as (±)-(3*S*,4*R*,6*S*).

Full NMR description of the three diastereoisomers of compound **4a** is given below:

**(±)-(3*S*,4*S*,6*S*)-1-(Benzyloxy)-4-hydroxy-6-methyl-3-((phenylthio)methyl)piperidin-2-one 4a$_1$** :

**$^1$H-NMR** (400 MHz, $CDCl_3$, 20 °C) δ 7.48 – 7.17 (m, 10H), 5.04 (d syst AB, *J* = 9.5 Hz, 1H), 4.81 (d syst AB, *J* = 9.5 Hz, 1H), 4.43 (bs, 1H), 4.06-3.95 (m, 1H), 3.85 (dd, *J* = 14.0, 4.0 Hz, 1H), 3.11 (dd, *J* = 14.0, 11.3 Hz, 1H), 2.60 (dt, *J* = 11.3, 3.5 Hz, 1H), 2.28 (d, *J* = 4.1 Hz, 1H), 2.16 (dt, *J* = 14.0, 4.3 Hz, 1H), 1.65 (t, *J* = 12.7 Hz, 1H), 1.30 (d, *J* = 6.2 Hz, 3H).

**$^{13}$C-NMR** (100 MHz, $CDCl_3$, 20 °C) δ (ppm) 166.1, 135.7, 135.4, 129.5 (2C), 129.3 (2C), 129.1 (2C),128.7, 128.6 (2C), 126.3, 76.5, 63.8, 52.4, 47.0, 38.5, 30.1, 20.0.

**(±)-(3*S*,4*R*,6*S*)-1-(benzyloxy)-4-hydroxy-6-methyl-3-((phenylthio)methyl)piperidin-2-one $\mathbf{4a_2}$** :

**$^1$H-NMR** (300 MHz, $CDCl_3$, 20 °C) δ 7.51 – 7.27 (m,), 7.23 – 7.18 (m, $1H_{17}$), 5.00 (d syst AB, *J* = 10 Hz, 1H), 4.89 (d syst AB, *J* = 10.0 Hz, 1H), 4.41 (m, 1H), 3.80 (dd, *J* = 13.8, 4.2 Hz, 1H), 3.75 – 3.65 (m, 1H), 3.18 (dd, *J* = 13.8, 10.7 Hz, 1H), 2.70 (dq, *J* = 10.6, 4.1 Hz, 1H), 2.01 (m, 3H), 1.45 (d, *J* = 6.6 Hz, 3H).

**$^{13}$C-NMR** (75 MHz, $CDCl_3$, 20 °C) δ (ppm) 167.1, 135.5, 129.5 (2C), 129.3 (5C), 128.8, 128.6 (2C), 126.5, 76.4, 65.1, 54.5, 47.2, 35.7, 30.7, 20.8.

**(±)-(3*R*,4*R*,6*S*)-1-(benzyloxy)-4-hydroxy-6-methyl-3-((phenylthio)methyl)piperidin-2-one $\mathbf{4a_3}$** :

**$^1$H-NMR** (300 MHz, $CDCl_3$, 20 °C) δ (ppm) 7.49 – 7.41 (m, 4H), 7.39 – 7.24 (m, 5H), 7.22 – 7.15 (m, 1H), 5.03 (d, *J* = 9.9 Hz, 1H), 4.77 (d, *J* = 9.9 Hz, 1H), 4.04 – 3.86 (m, 1H), 3.67 (dd, *J* = 13.7, 4.0 Hz, 1H), 3.57 – 3.46 (m, 1H), 3.42 (dd, *J* = 13.7, 5.9 Hz, 1H), 2.75 – 2.61 (m, 2H), 2.15 (dt, *J* = 13.1, 4.2 Hz, 1H), 1.66 (dt, *J* = 13.2, 11.2 Hz, 1H), 1.29 (d, *J* = 6.2 Hz, 3H).

**$^{13}$C-NMR** (75 MHz, $CDCl_3$, 20 °C) δ (ppm) 167.0, 136.1, 135.3, 129.7(2C), 129.5(2C), 129.3(2C), 128.9, 128.6(2C), 126.5, 76.6, 67.3, 53.2, 50.5, 40.0, 32.9, 20.2.

# $^{1}H$ and $^{13}C\{1H\}$ NMR of compounds 4a-4z

## (±)-(3*S*,4*S*,6*S*)-1-(Benzyloxy)-4-hydroxy-6-methyl-3-((phenylthio)methyl)piperidin-2-one 4a$_1$

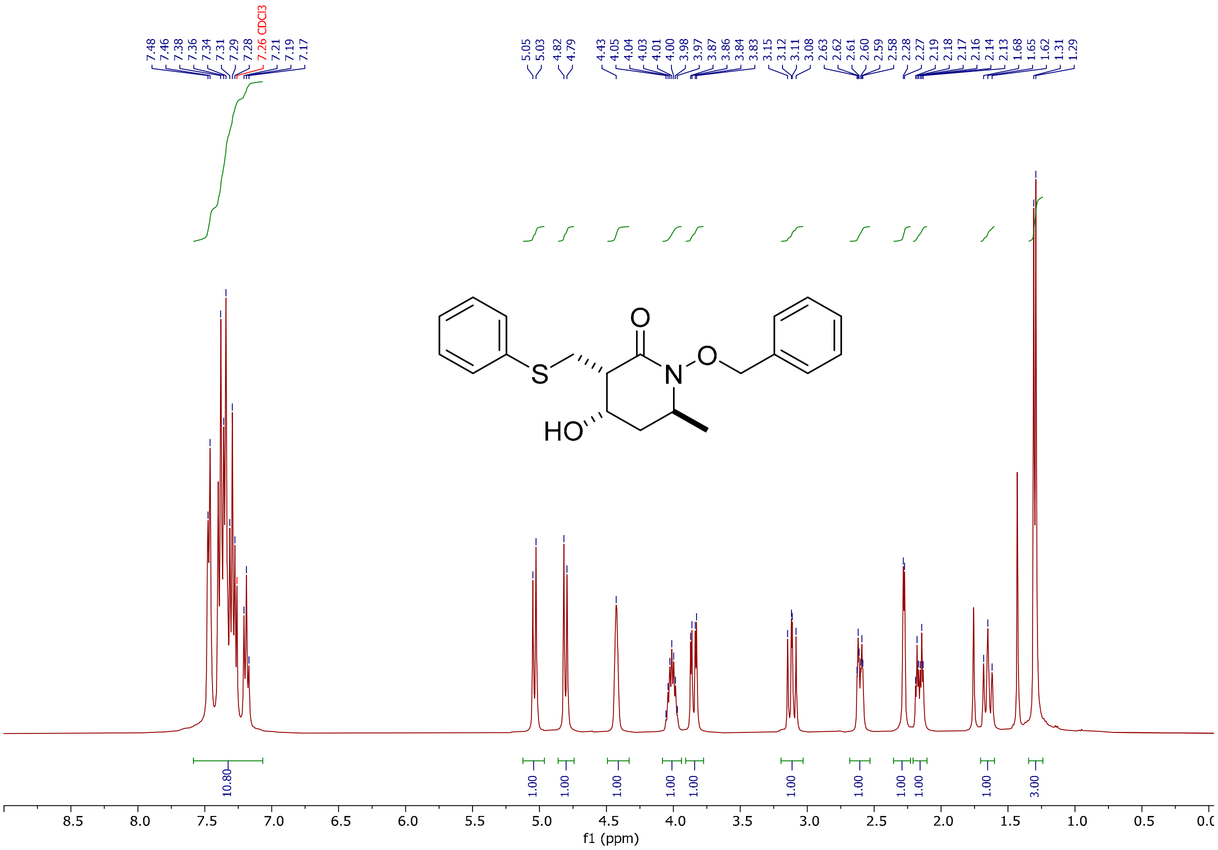


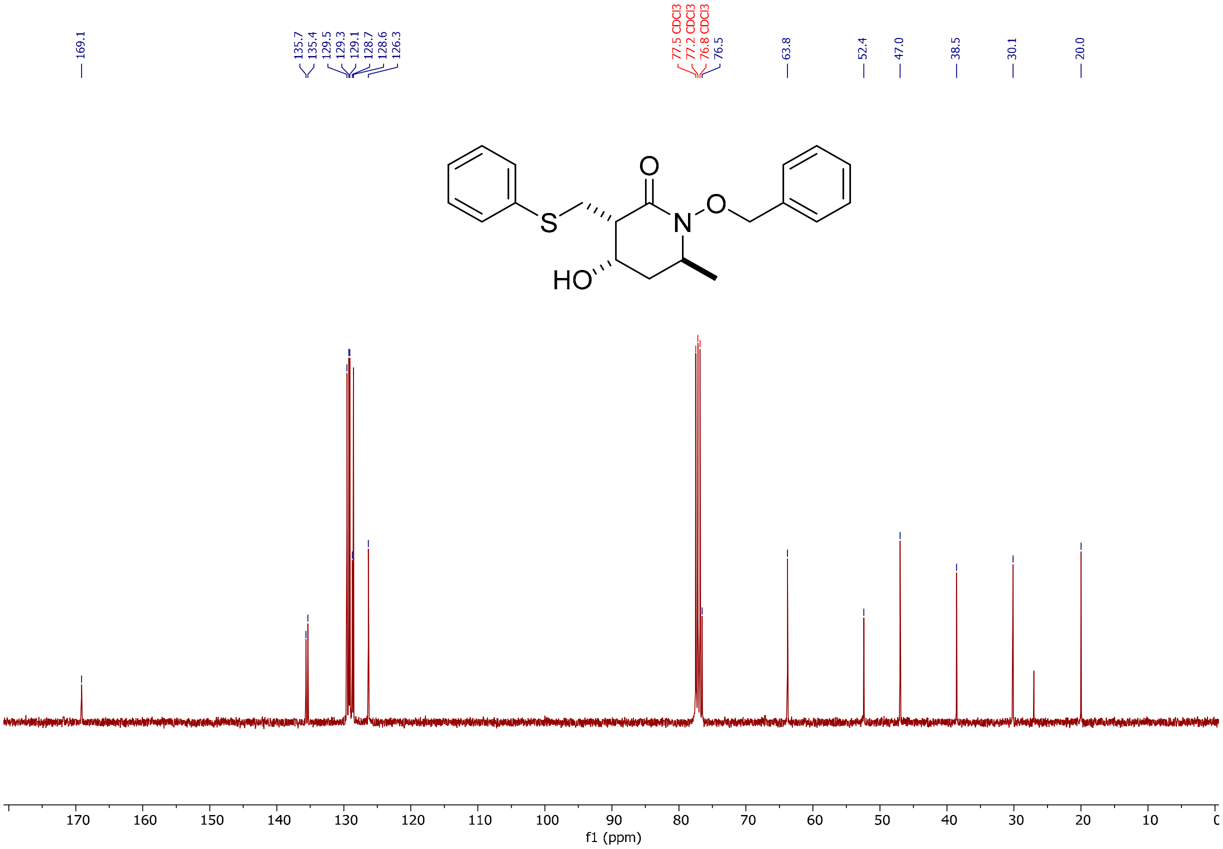

**(±)-(3*S*,4*R*,6*S*)-1-(Benzyloxy)-4-hydroxy-6-methyl-3-((phenylthio)methyl)piperidin-2-one 4a$_2$**

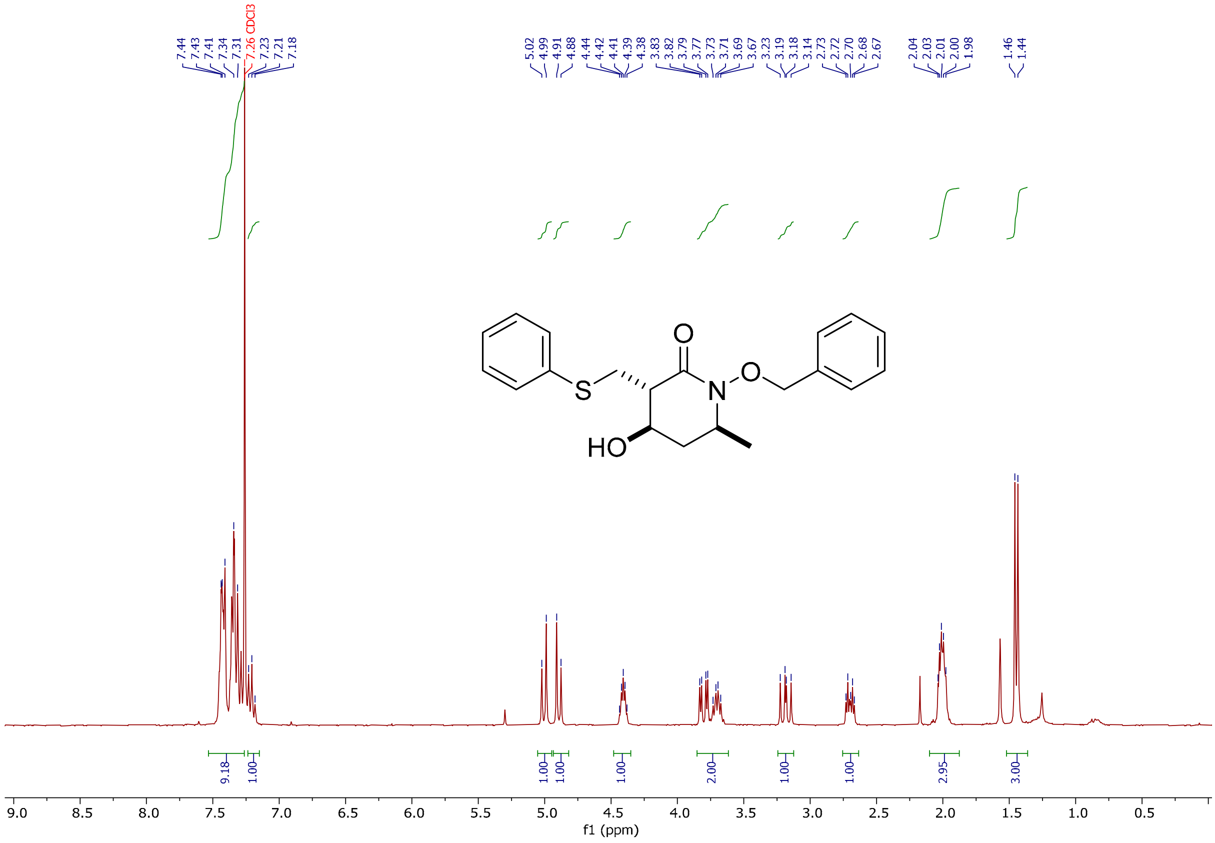

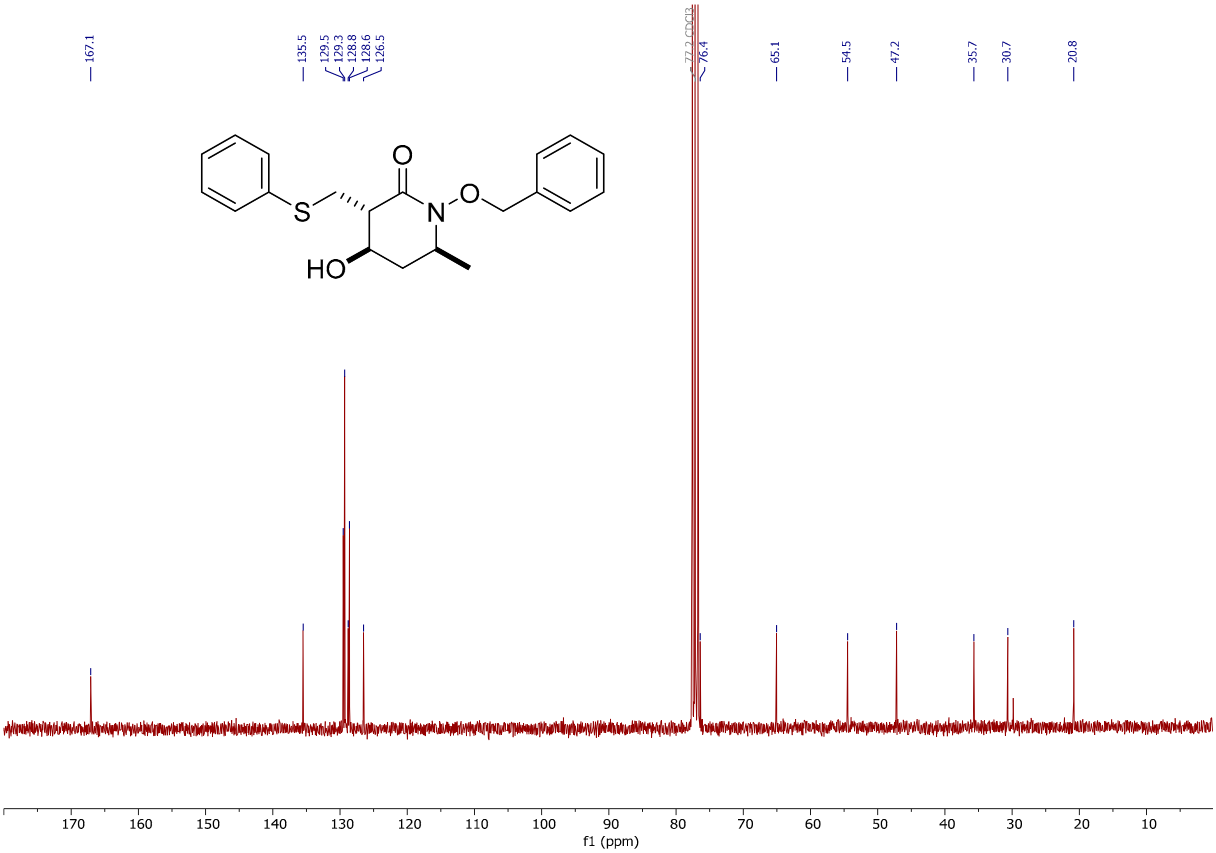

**(±)-(3*R*,4*R*,6*S*)-1-(benzyloxy)-4-hydroxy-6-methyl-3-((phenylthio)methyl)piperidin-2-one 4a$_3$**

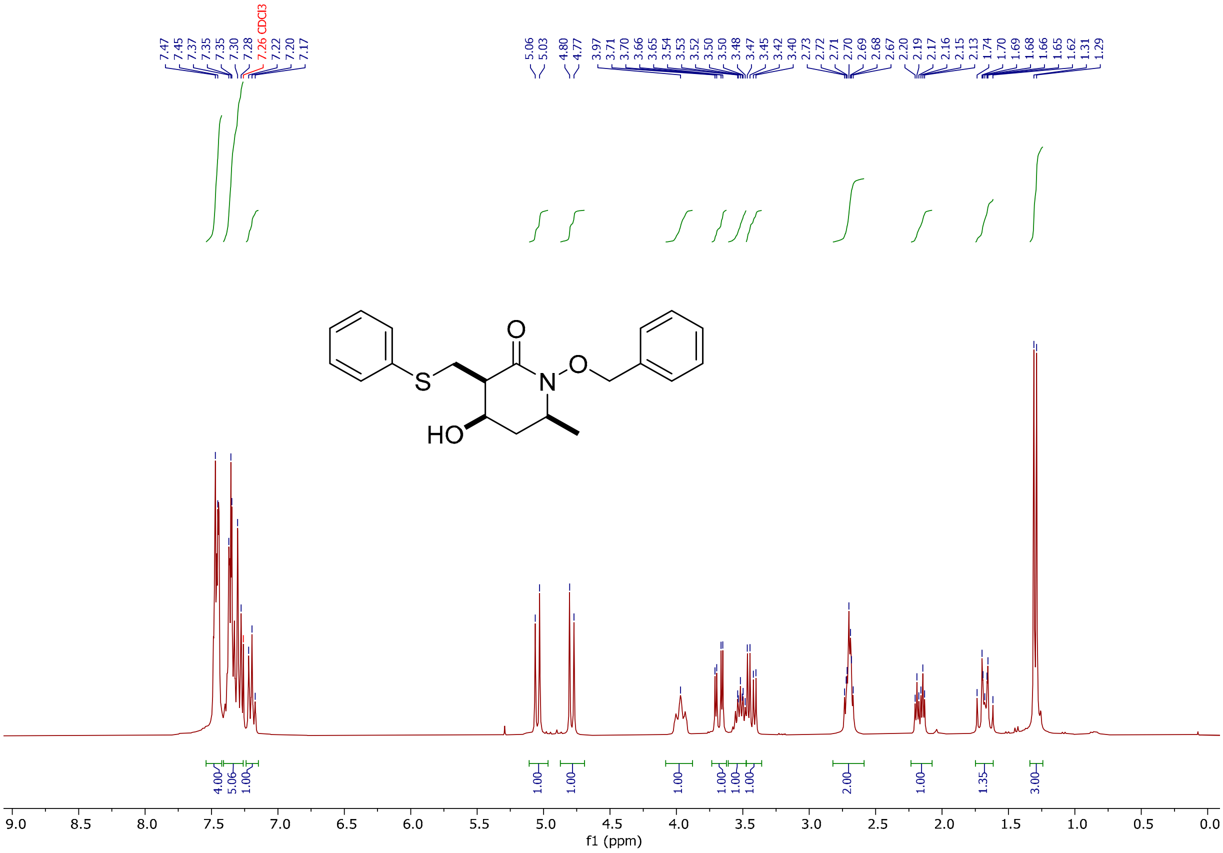


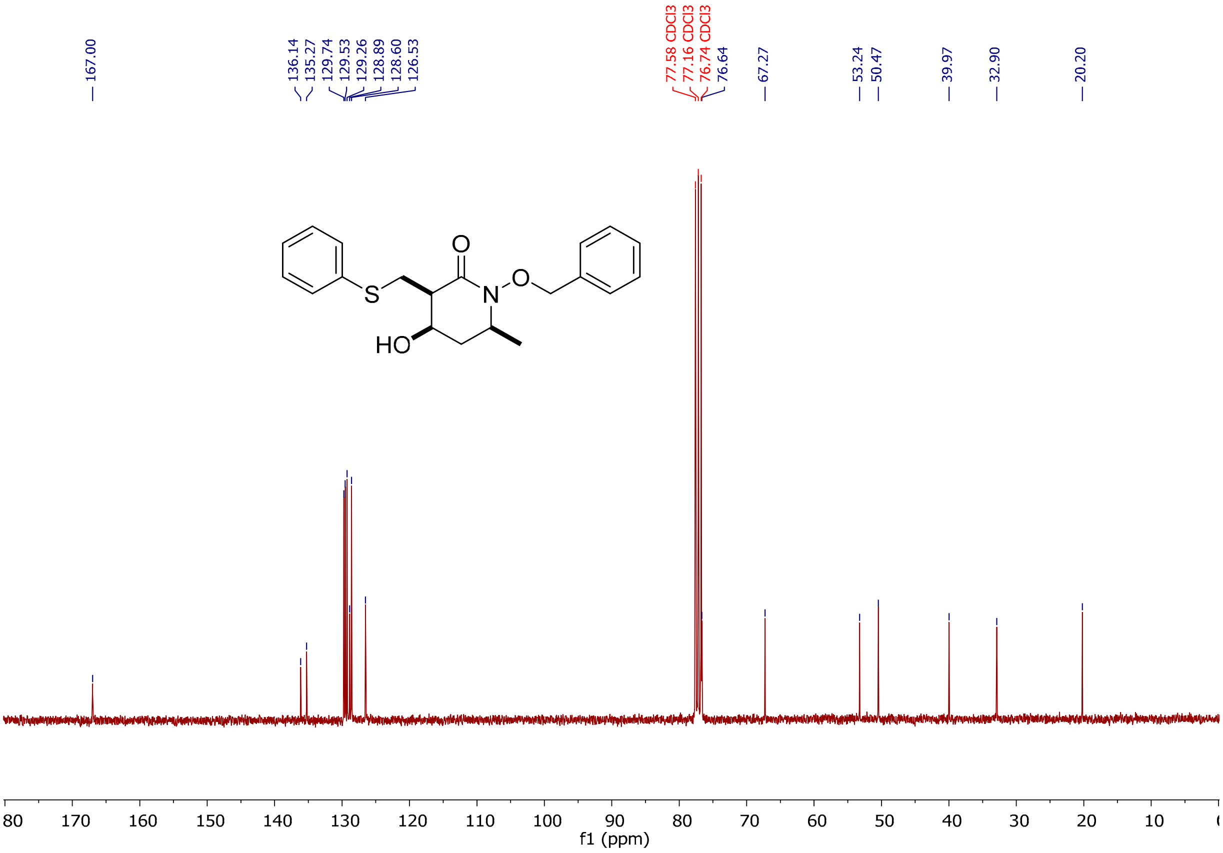

**(±)-(3*S*,4*S*,6*S*)-1-(Benzyloxy)-6-ethyl-4-hydroxy-3-((phenylthio)methyl)piperidin-2-one 4b**

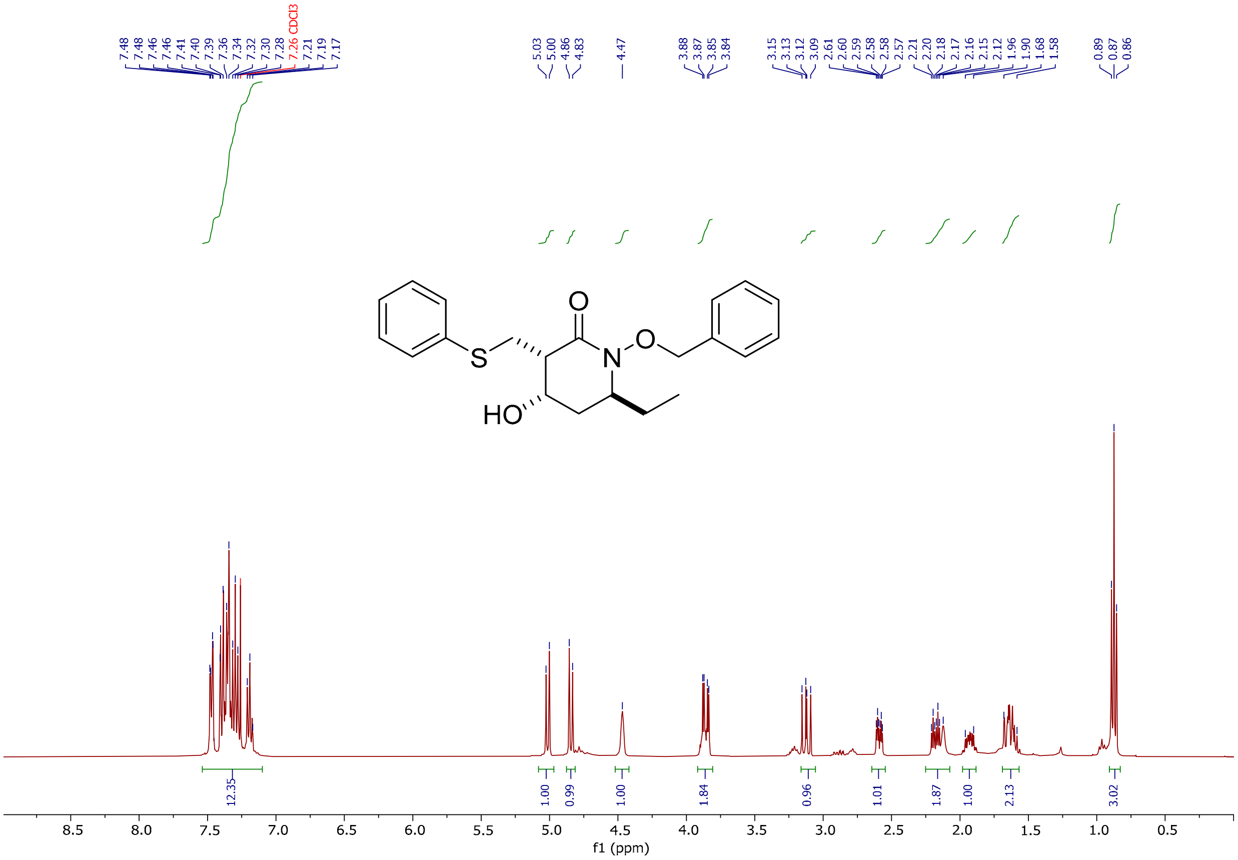

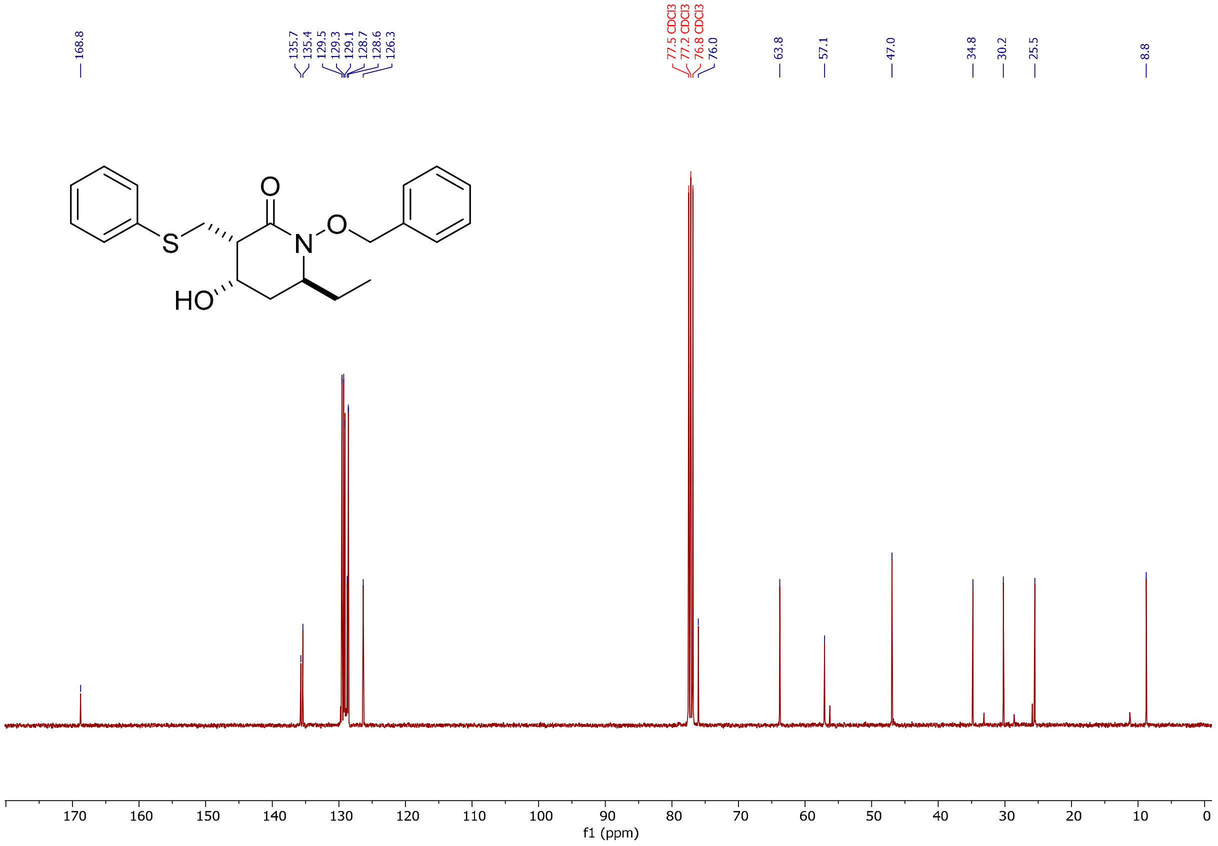

**(±)-(3*S*,4*S*,6*S*)-1-(Benzyloxy)-6-ethyl-4-hydroxy-3-((phenylthio)methyl)piperidin-2-one 4c**

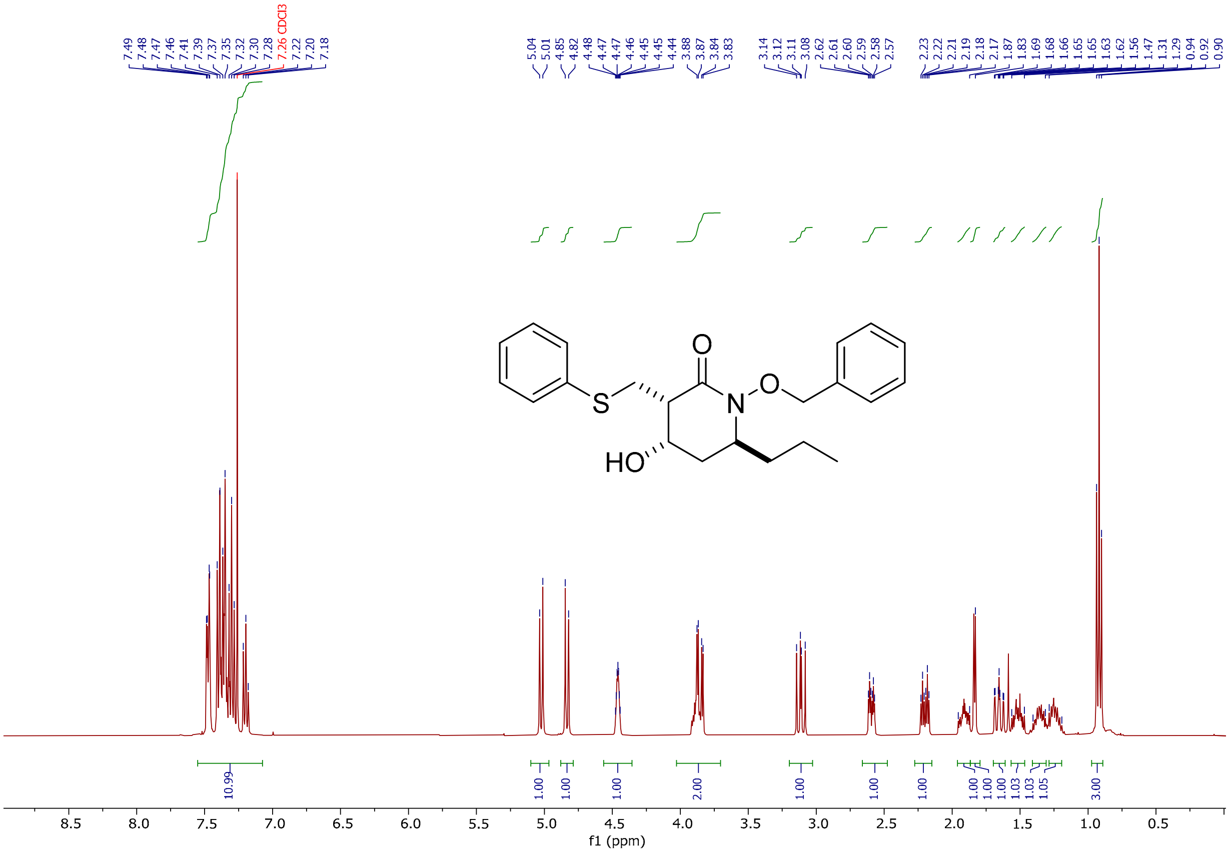

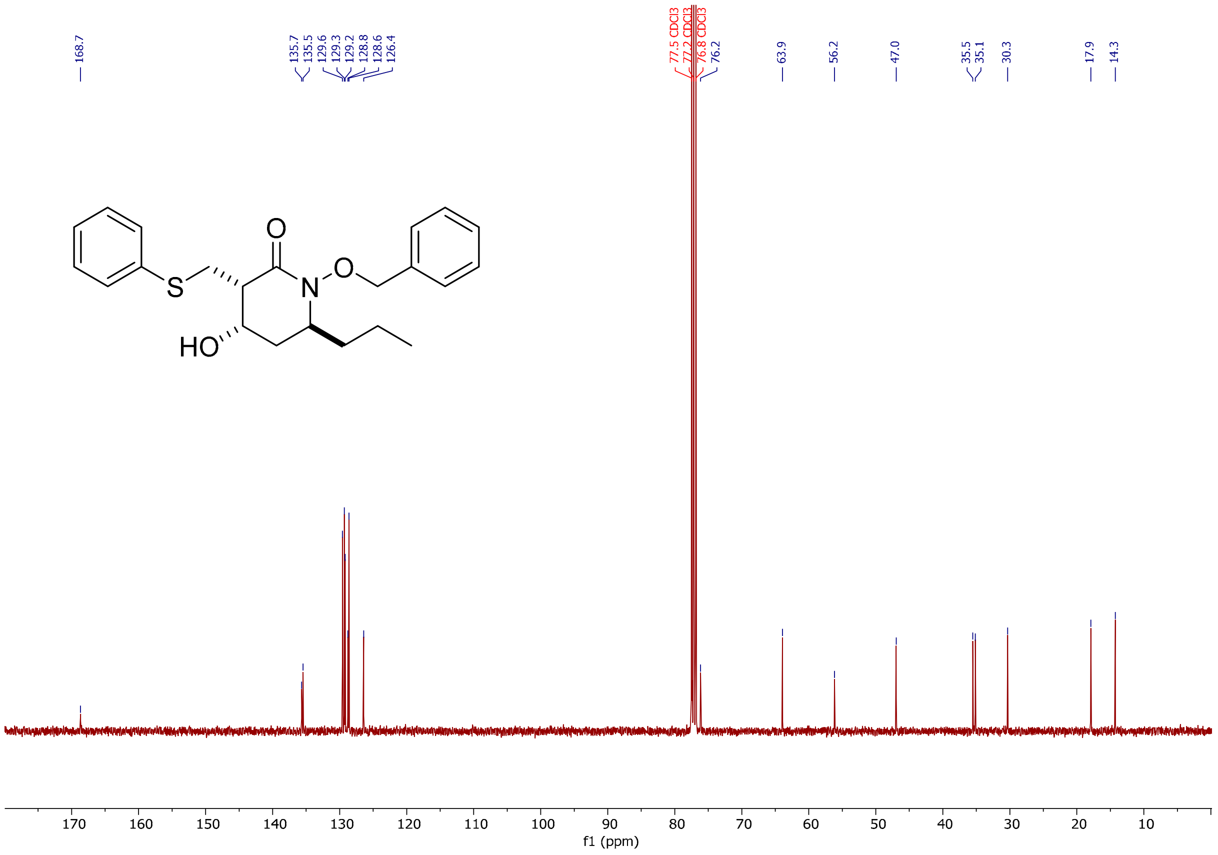

**(±)-(3*S*,4*S*,6*S*)-4-Hydroxy-1-methoxy-6-methyl-3-((phenylthio)methyl)piperidin-2-one 4d**

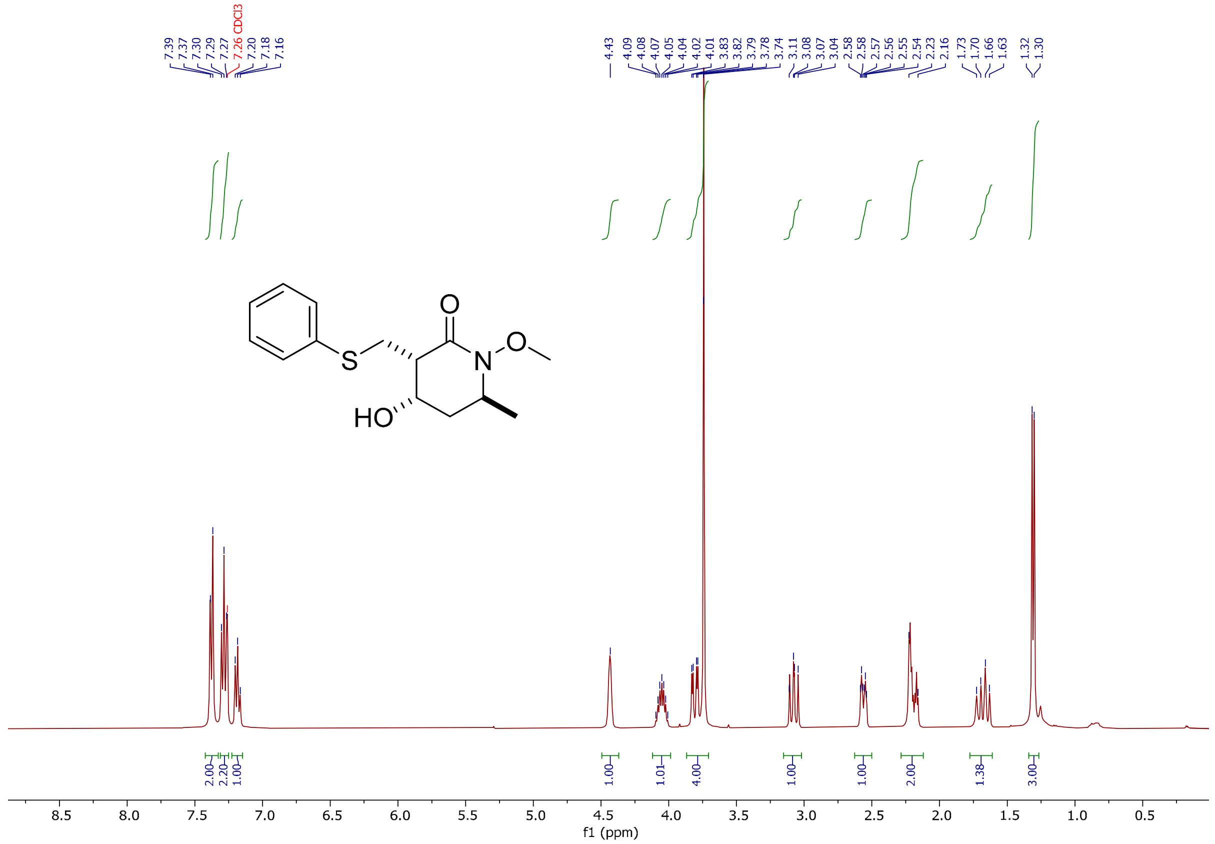


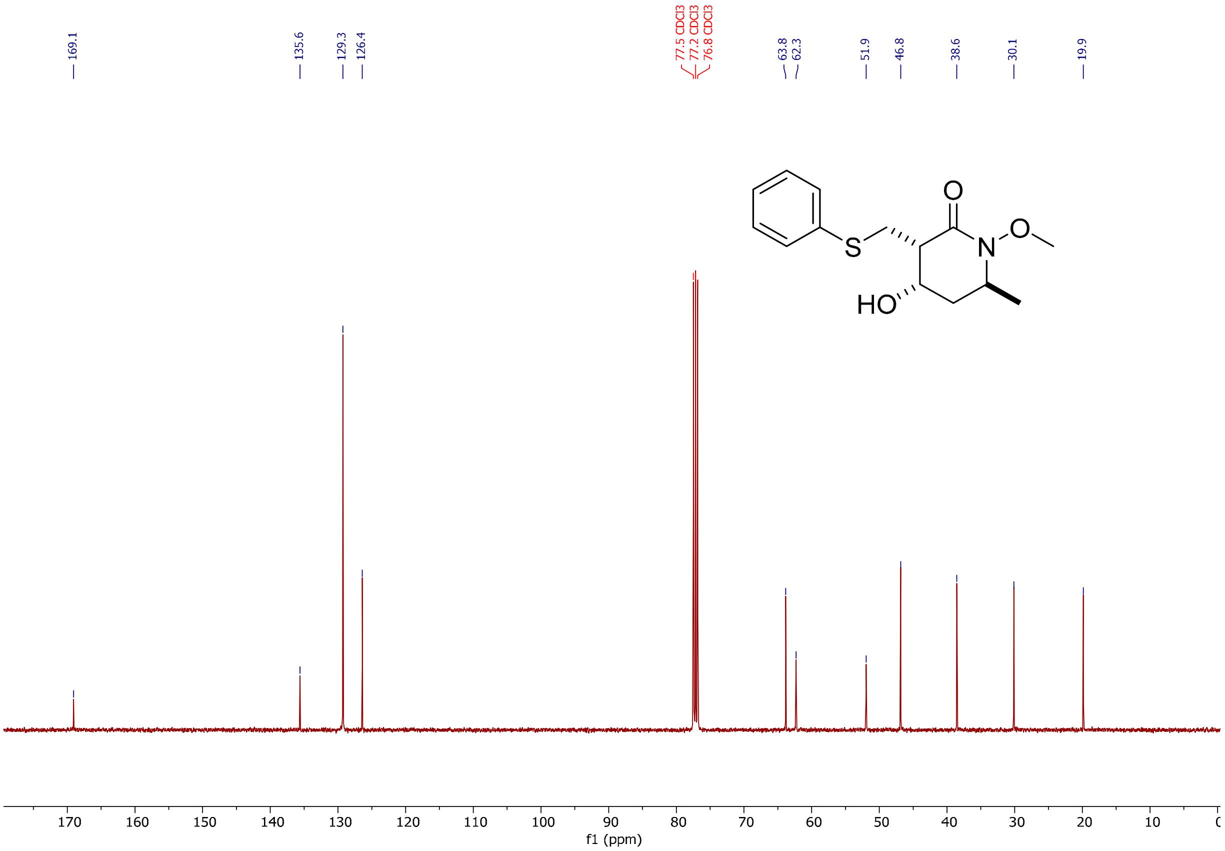

**(±)-(3*S*,4*S*,6*S*)-1-(Benzyloxy)-4-hydroxy-4,6-dimethyl-3-((phenylthio)methyl)piperidin-2-one 4e**

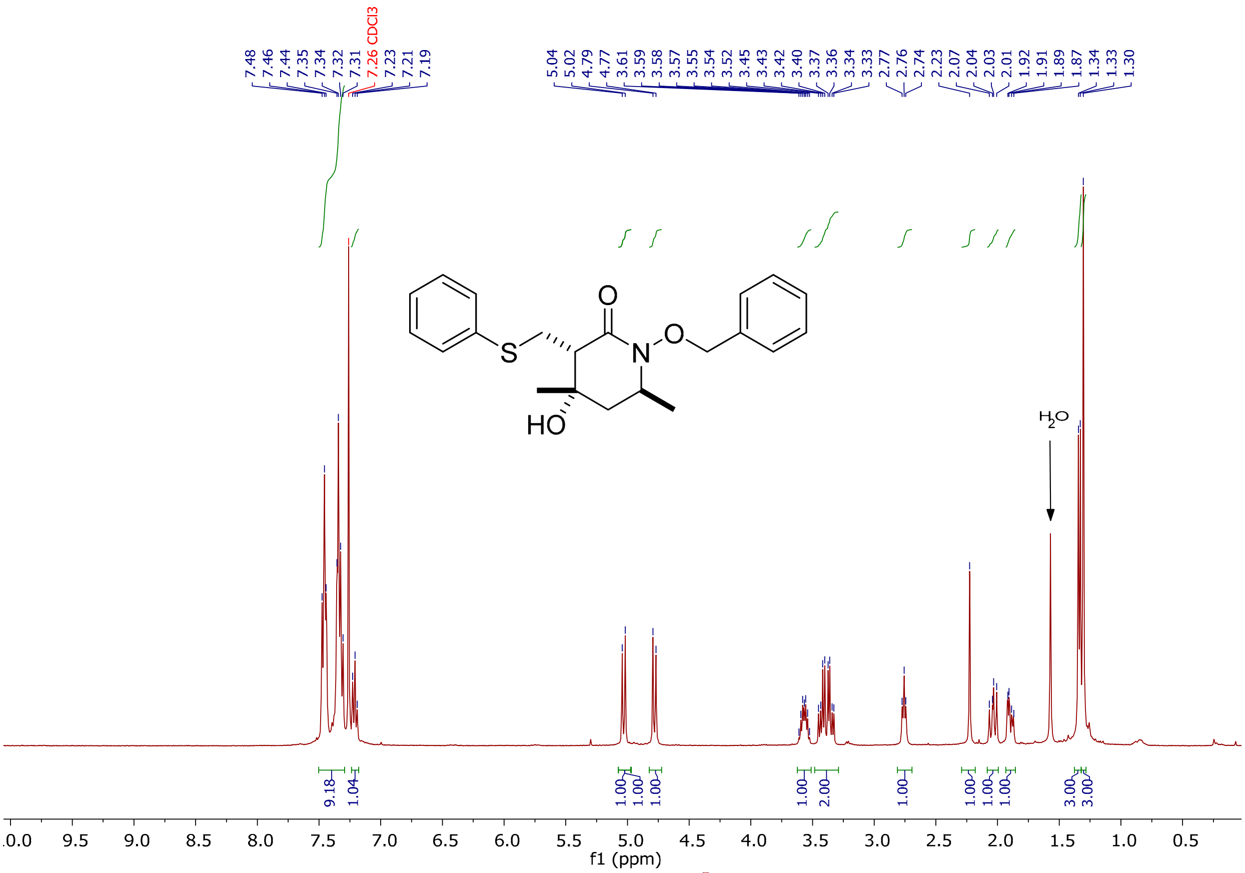


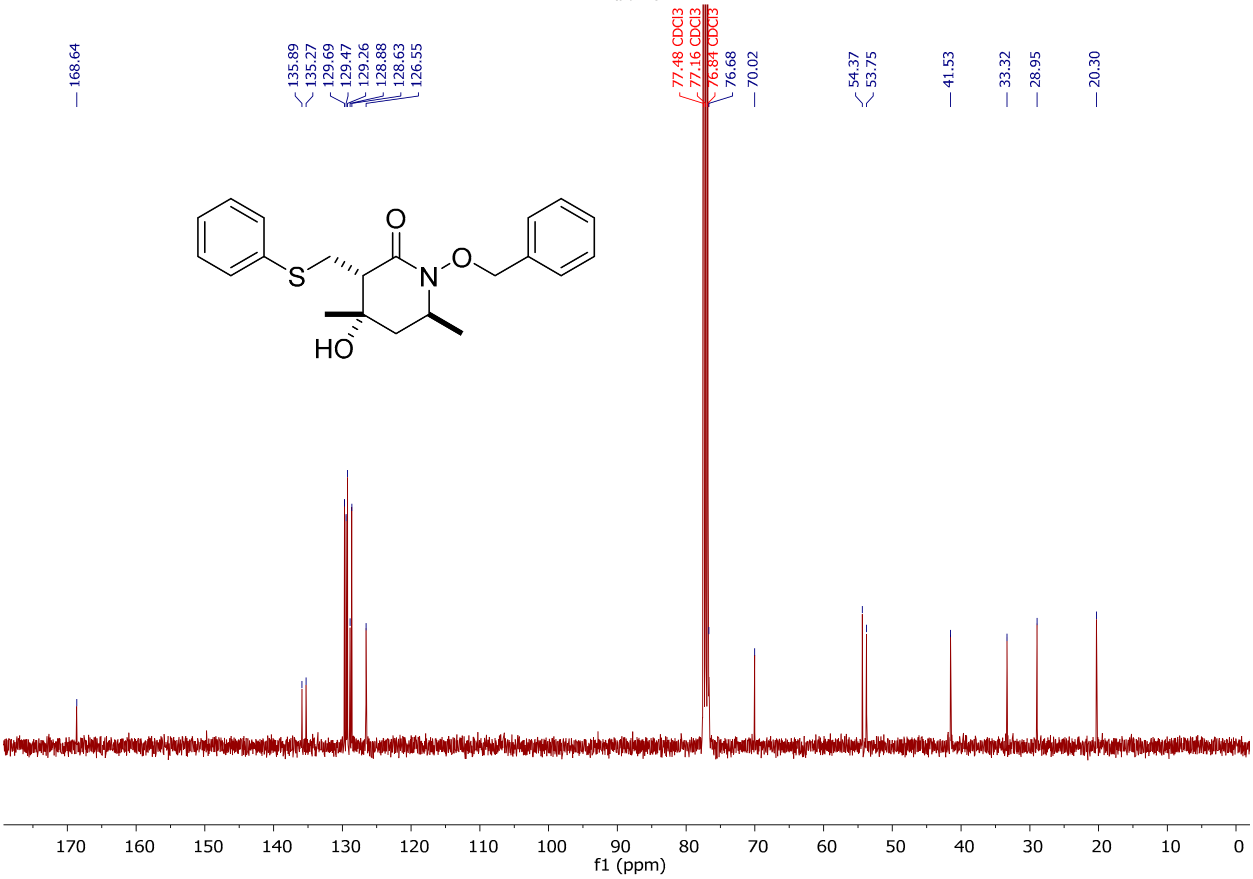

**(±)-(3*S*,4*S*,6*S*)-1-(Benzyloxy)-4-hydroxy-6-methyl-3-((o-tolylthio)methyl)piperidin-2-one 4f**

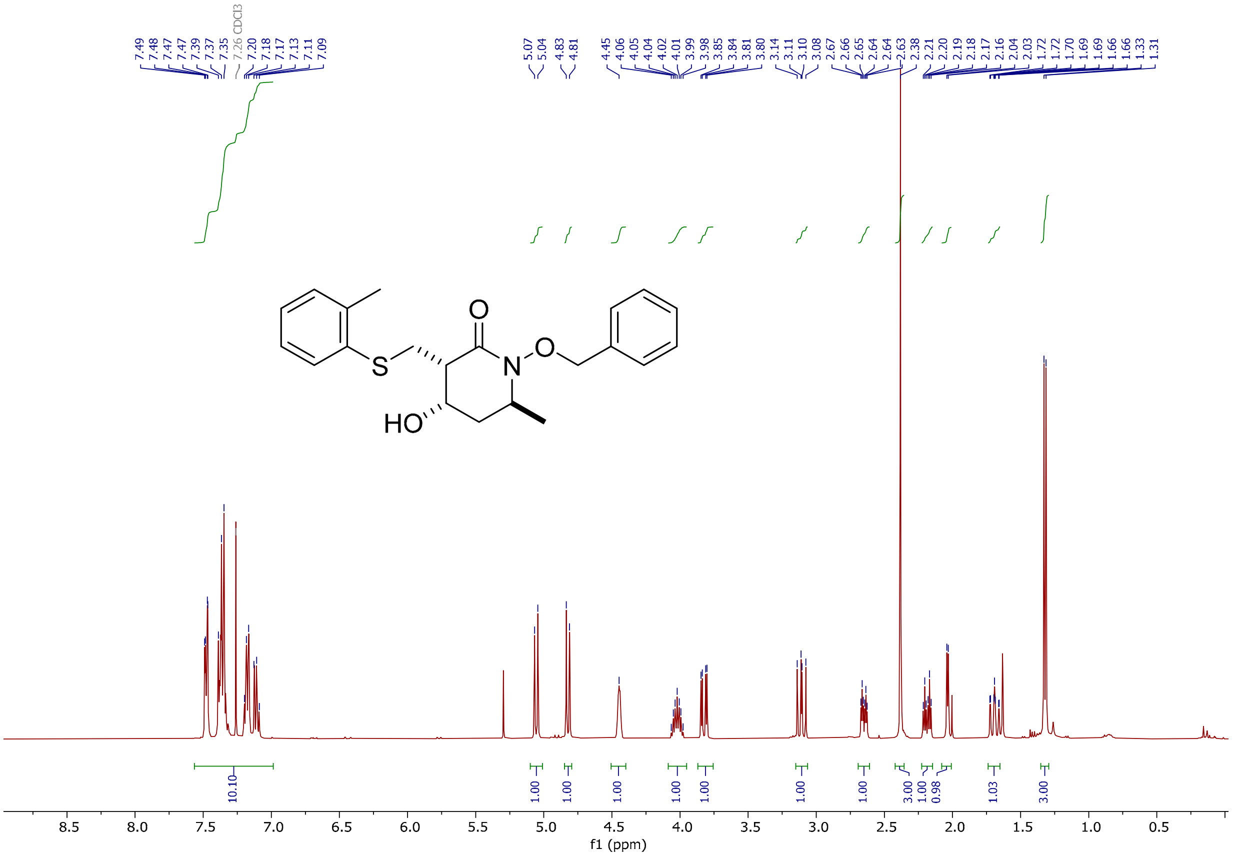

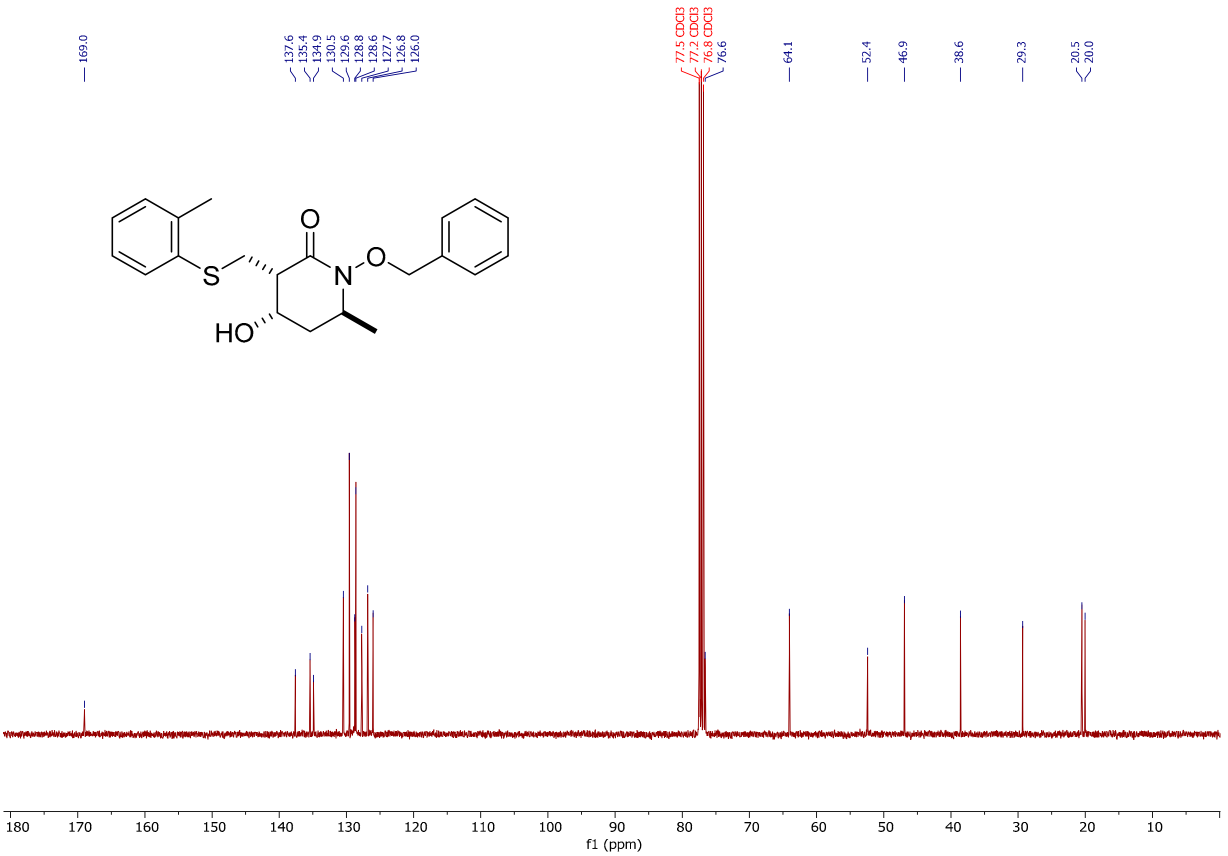

**(±)-(3*S*,4*S*,6*S*)-1-(Benzyloxy)-3-(((2,4-dimethylphenyl)thio)methyl)-4-hydroxy-6-methyl piperidin-2-one 4g**

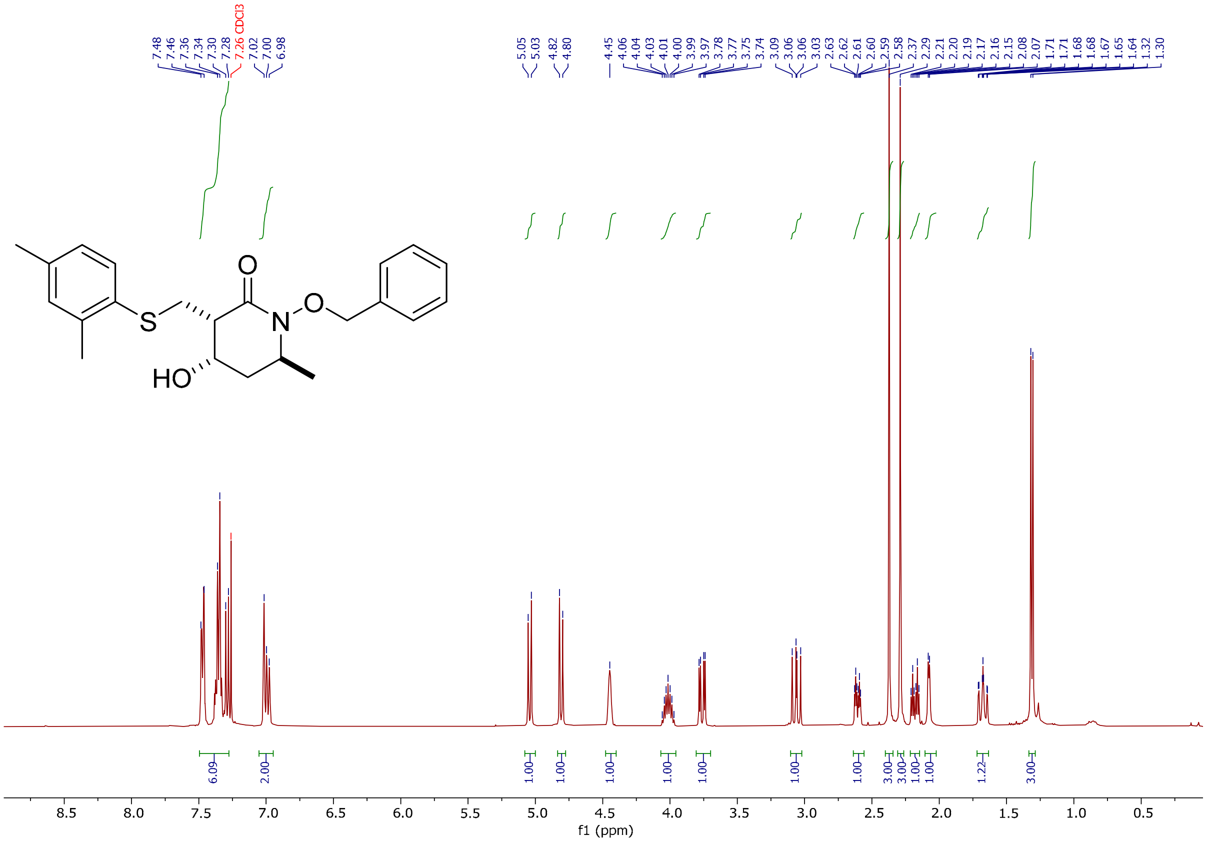


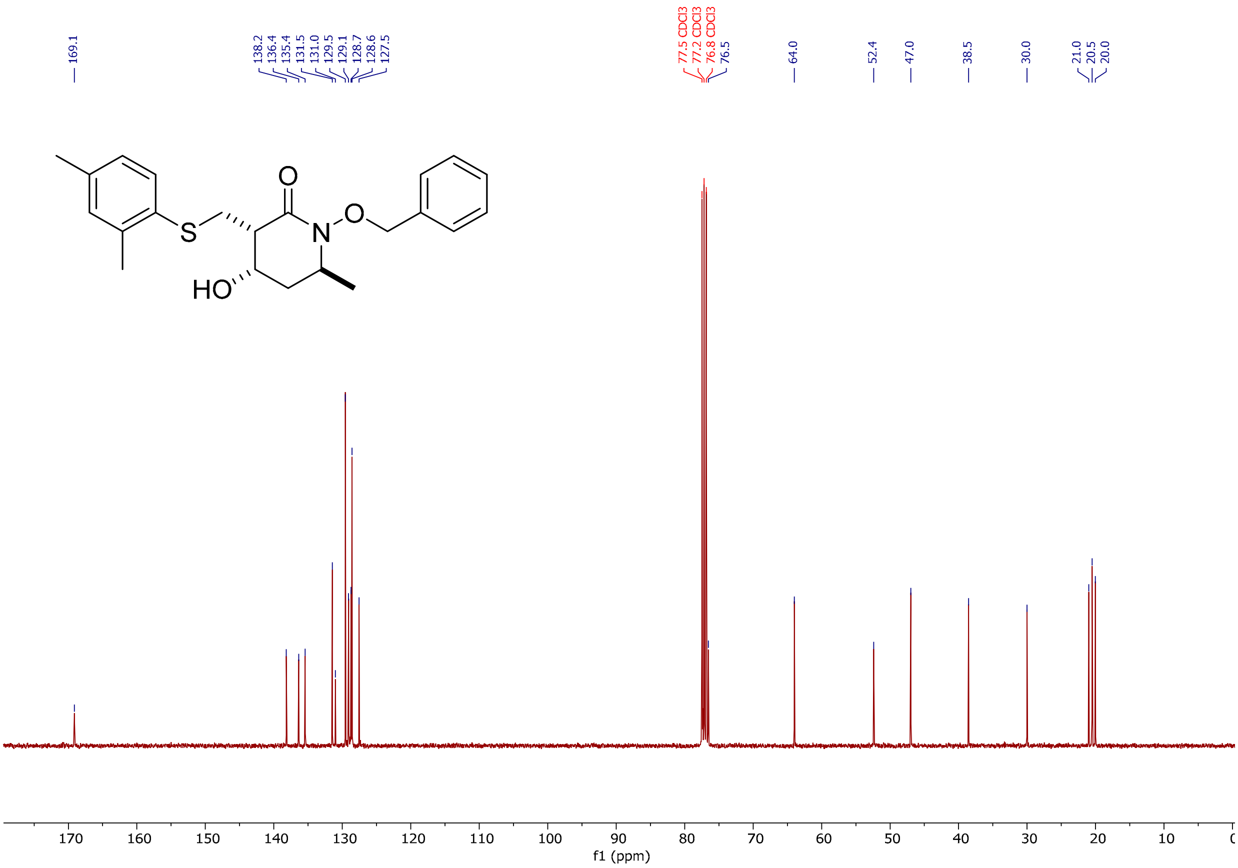

**(±)-(3*S*,4*S*,6*S*)-1-(Benzyloxy)-3-(((2,6-dimethylphenyl)thio)methyl)-4-hydroxy-6-methylpiperidin-2-one 4h**

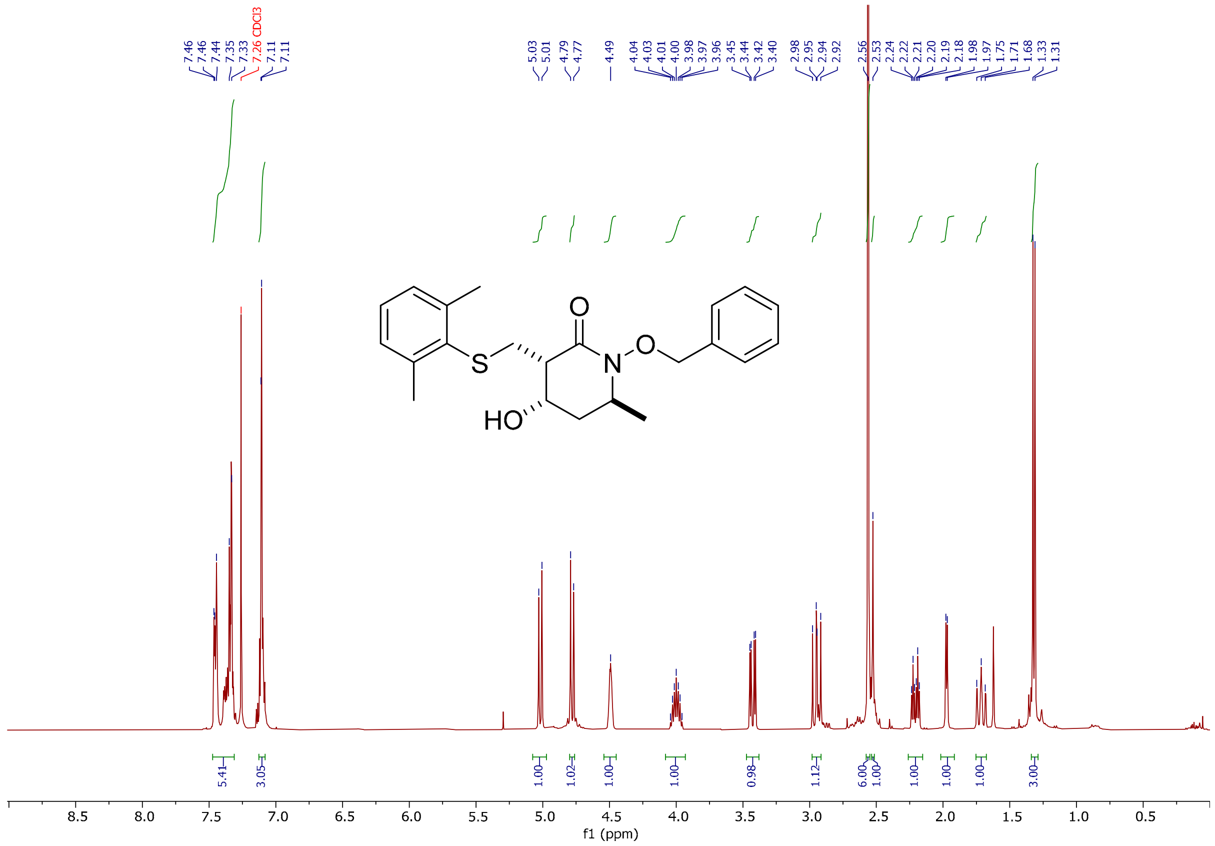

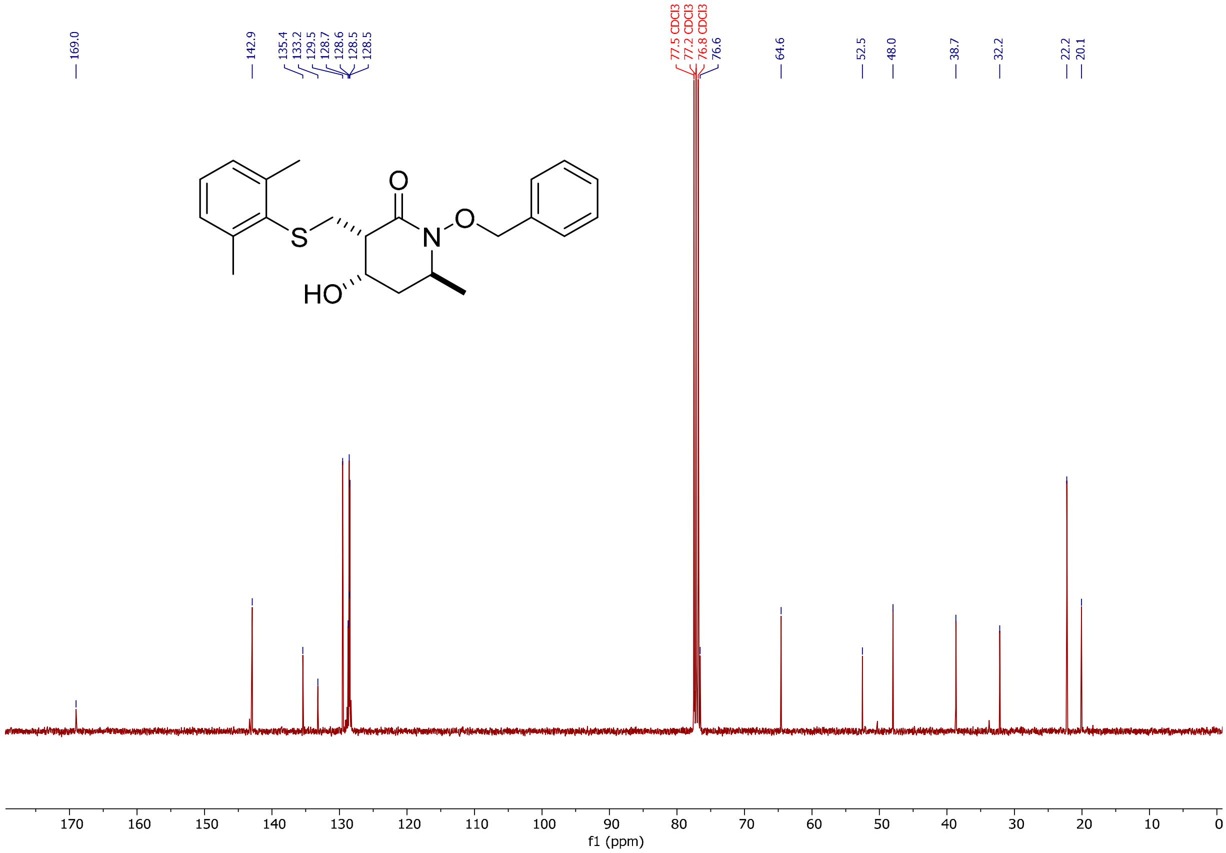

**(±)-(3*S*,4*S*,6*S*)-3-(([1,1'-Biphenyl]-4-ylthio)methyl)-1-(benzyloxy)-4-hydroxy-6-methylpiperidin-2-one 4i**

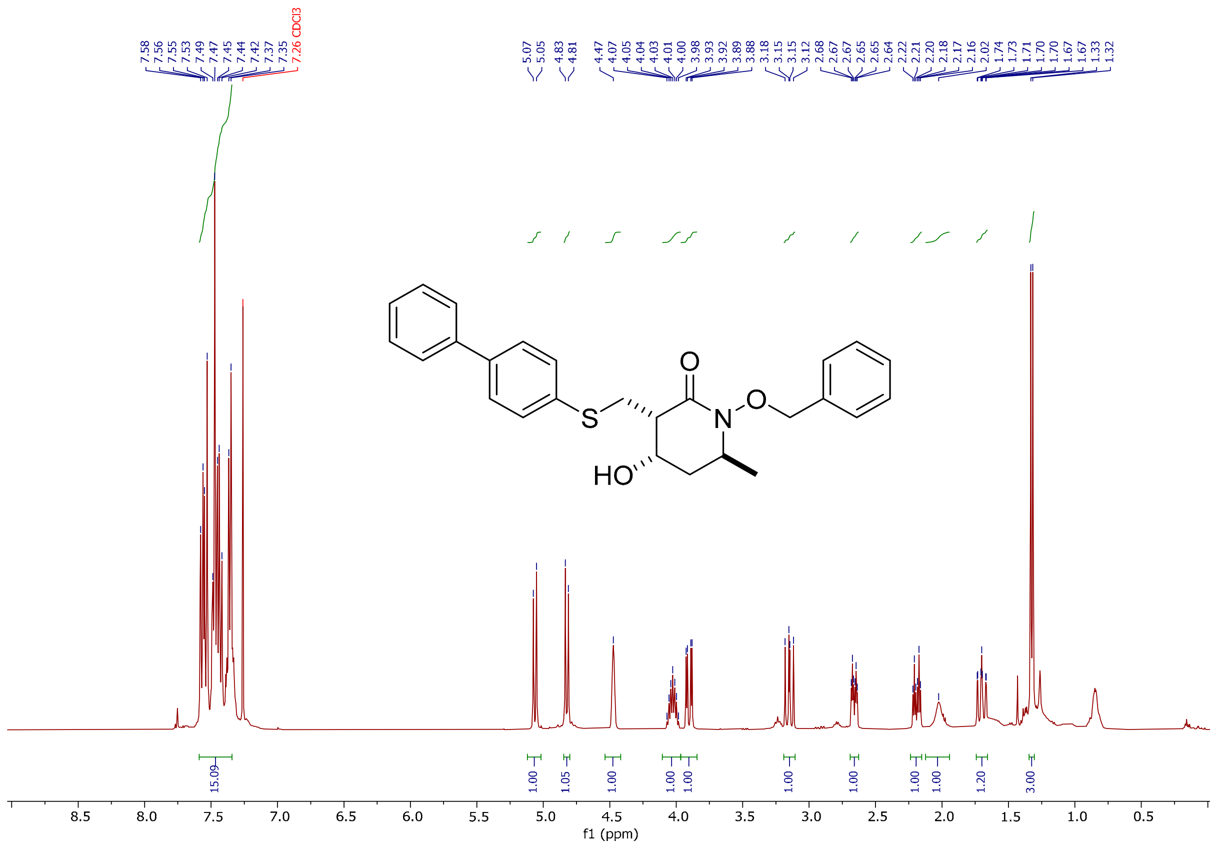


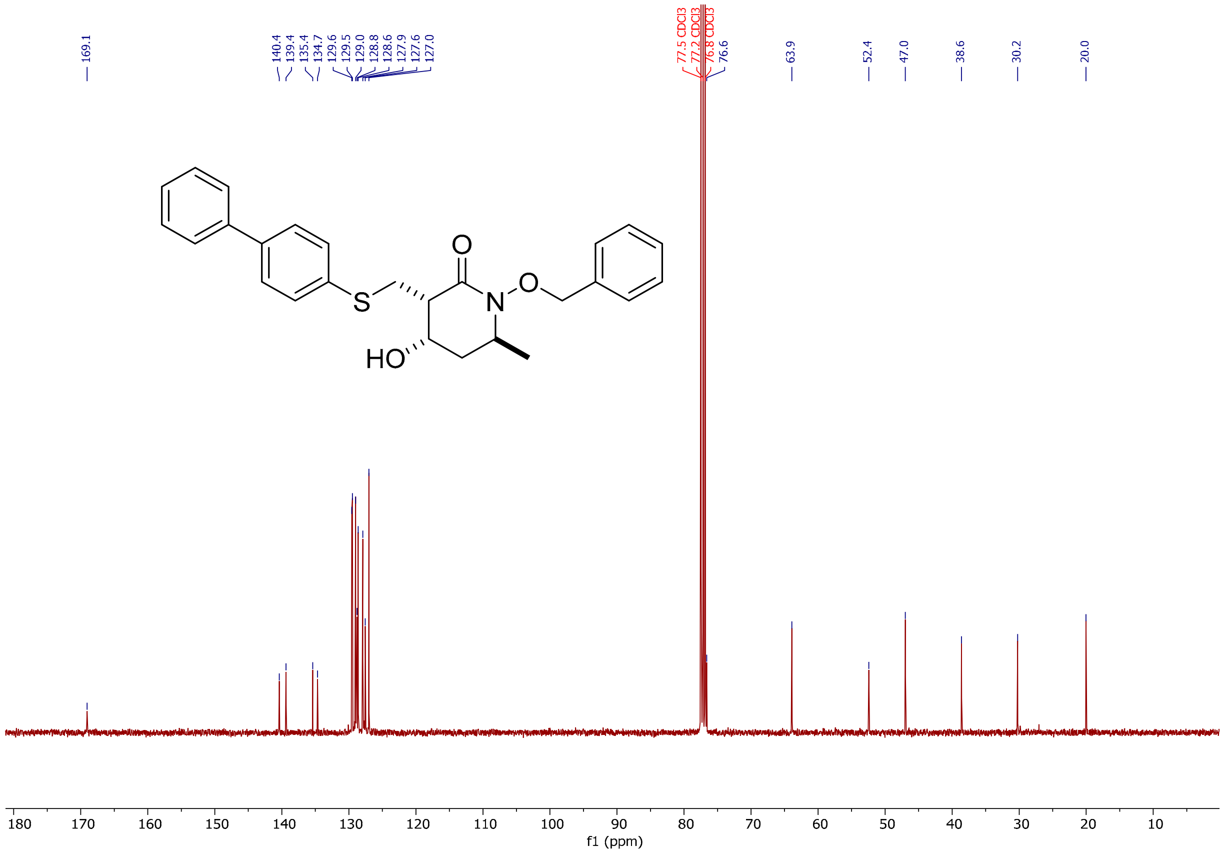

**(±)-(3*S*,4*S*,6*S*)-1-(Benzyloxy)-3-(((4-(tert-butyl)phenyl)thio)methyl)-4-hydroxy-6-methylpiperidin-2-one 4j**

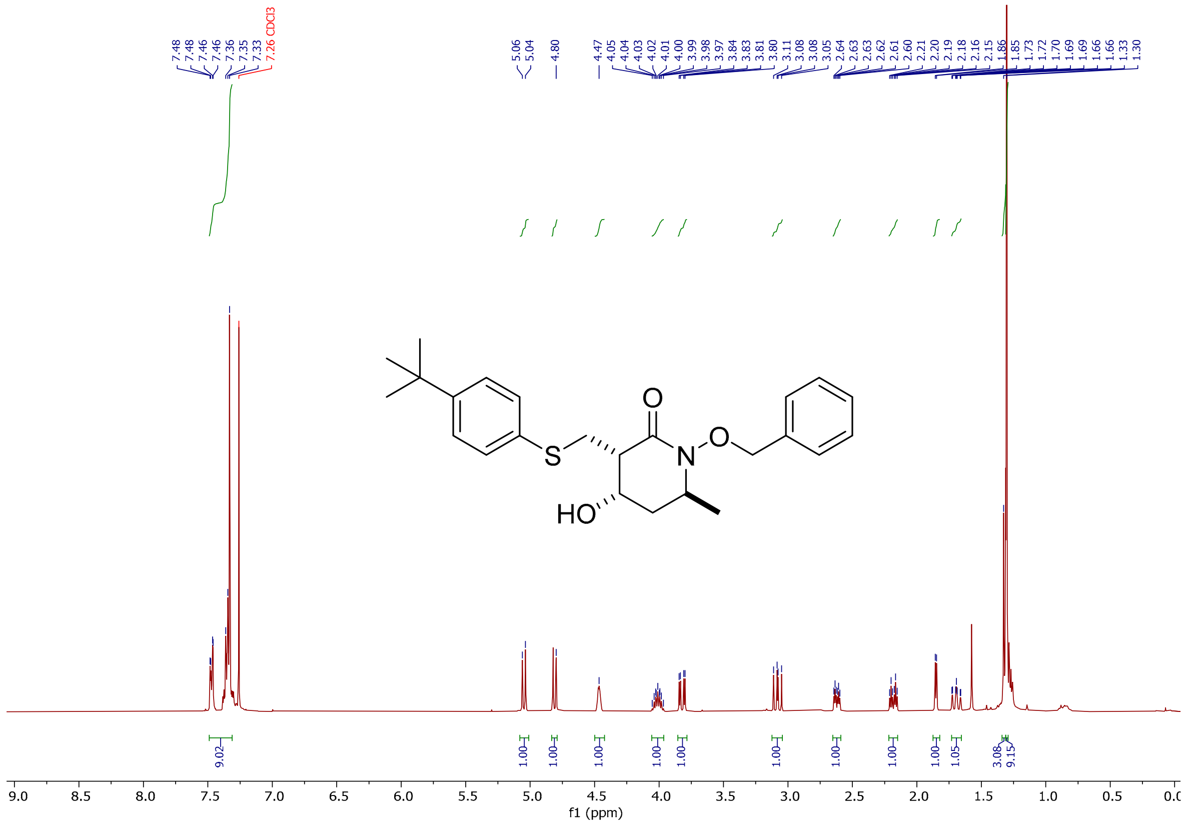


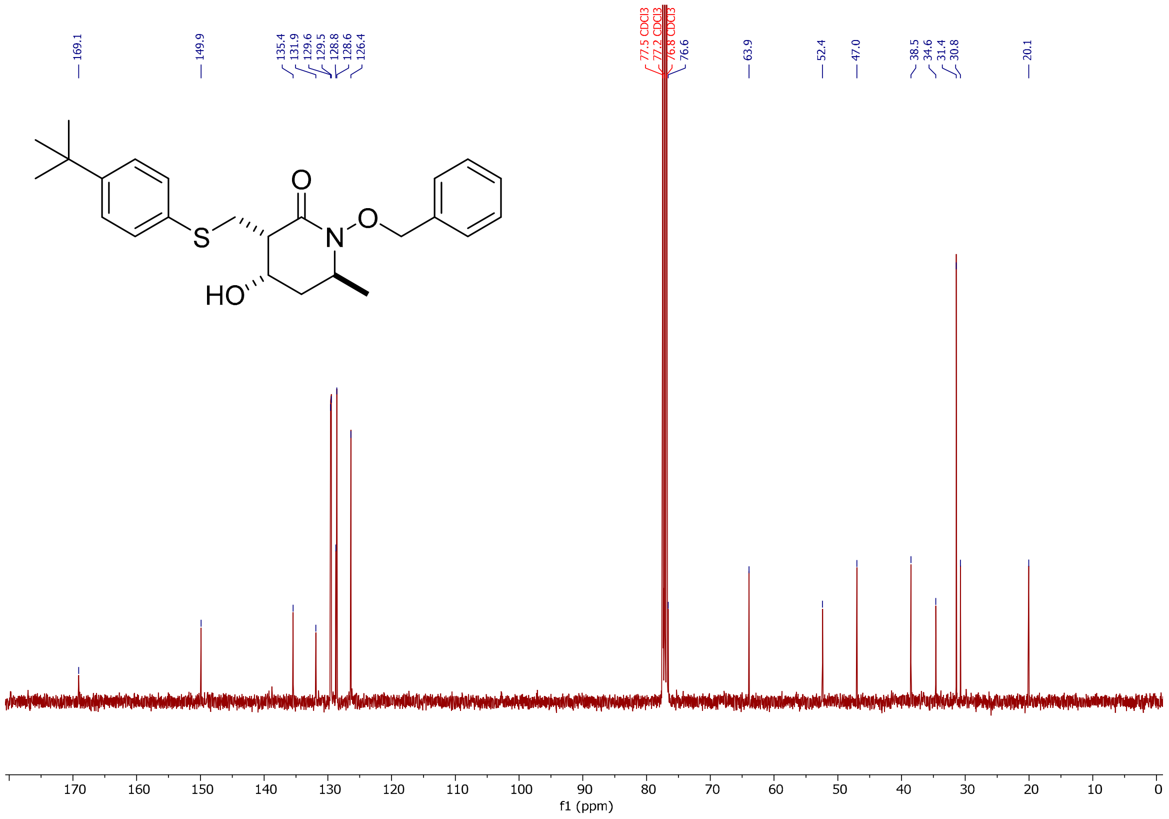

**(±)-(3*S*,4*S*,6*S*)-1-(Benzyloxy)-4-hydroxy-6-methyl-3-((naphthalen-2-ylthio)methyl)piperidin-2-one 4k**

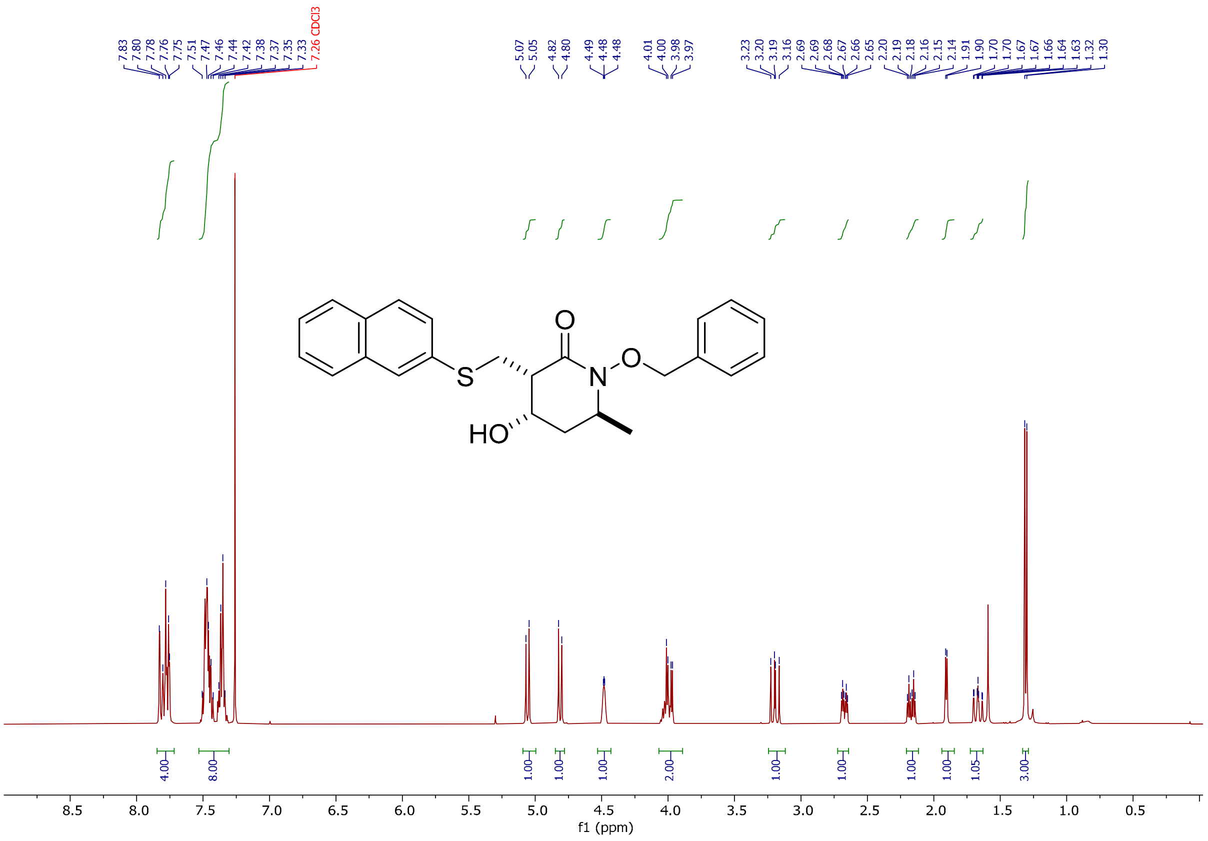

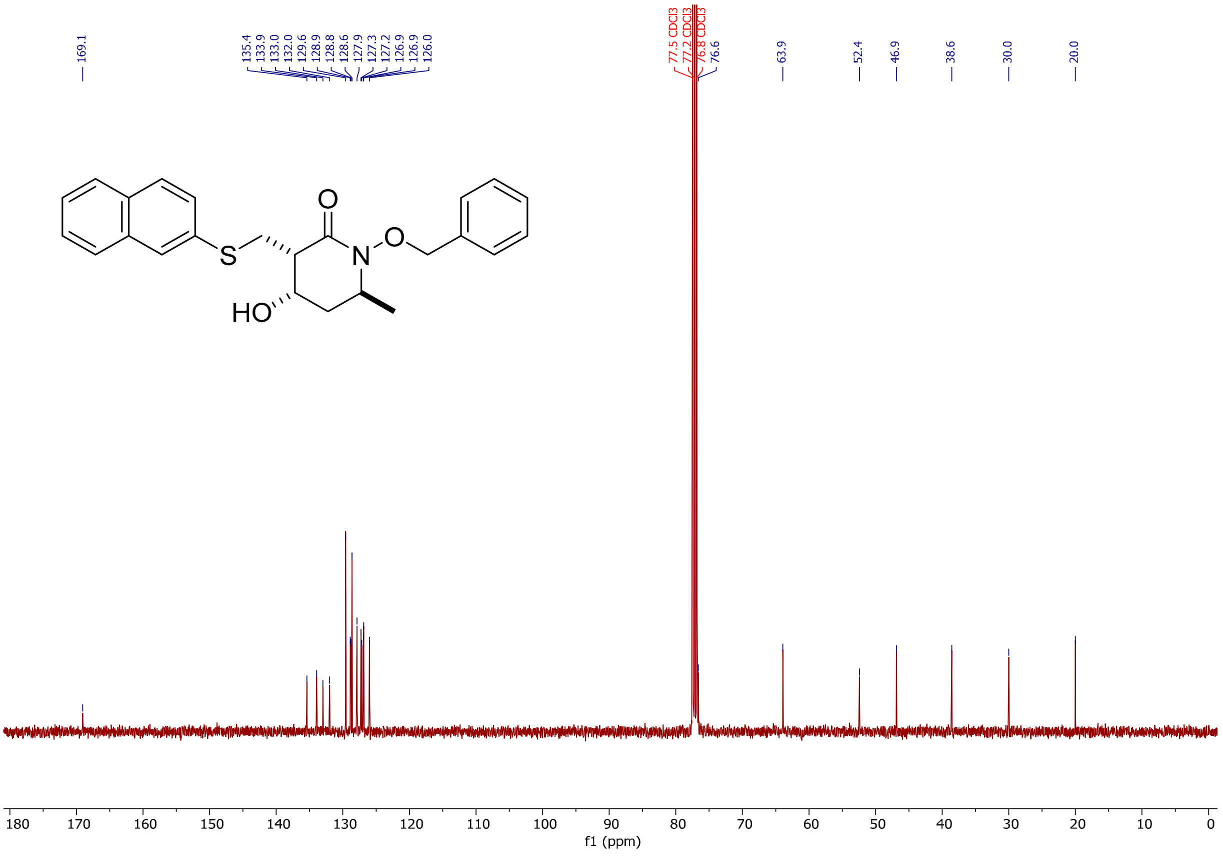

**(±)-(3*S*,4*S*,6*S*)-1-(Benzyloxy)-4-hydroxy-3-(((4-methoxyphenyl)thio)methyl)-6-methylpiperidin-2-one 4l**

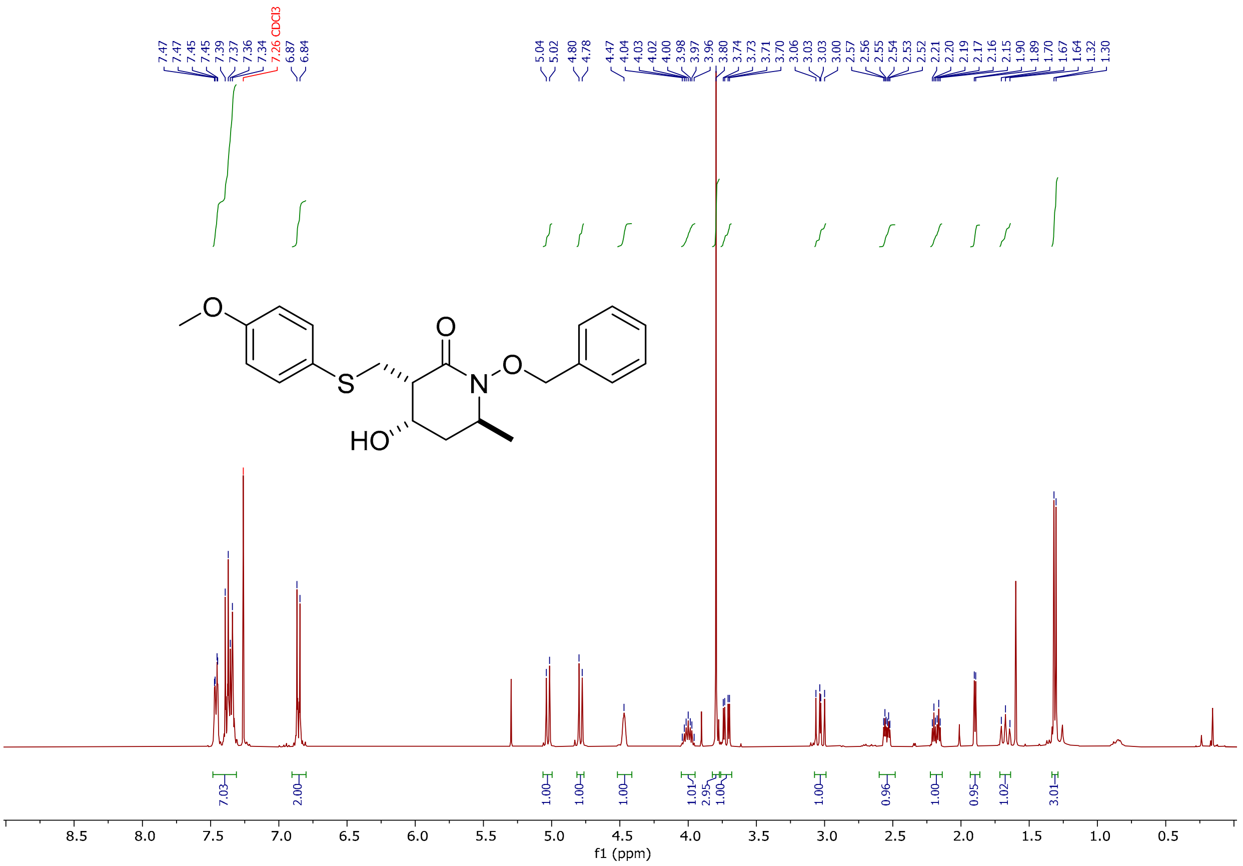

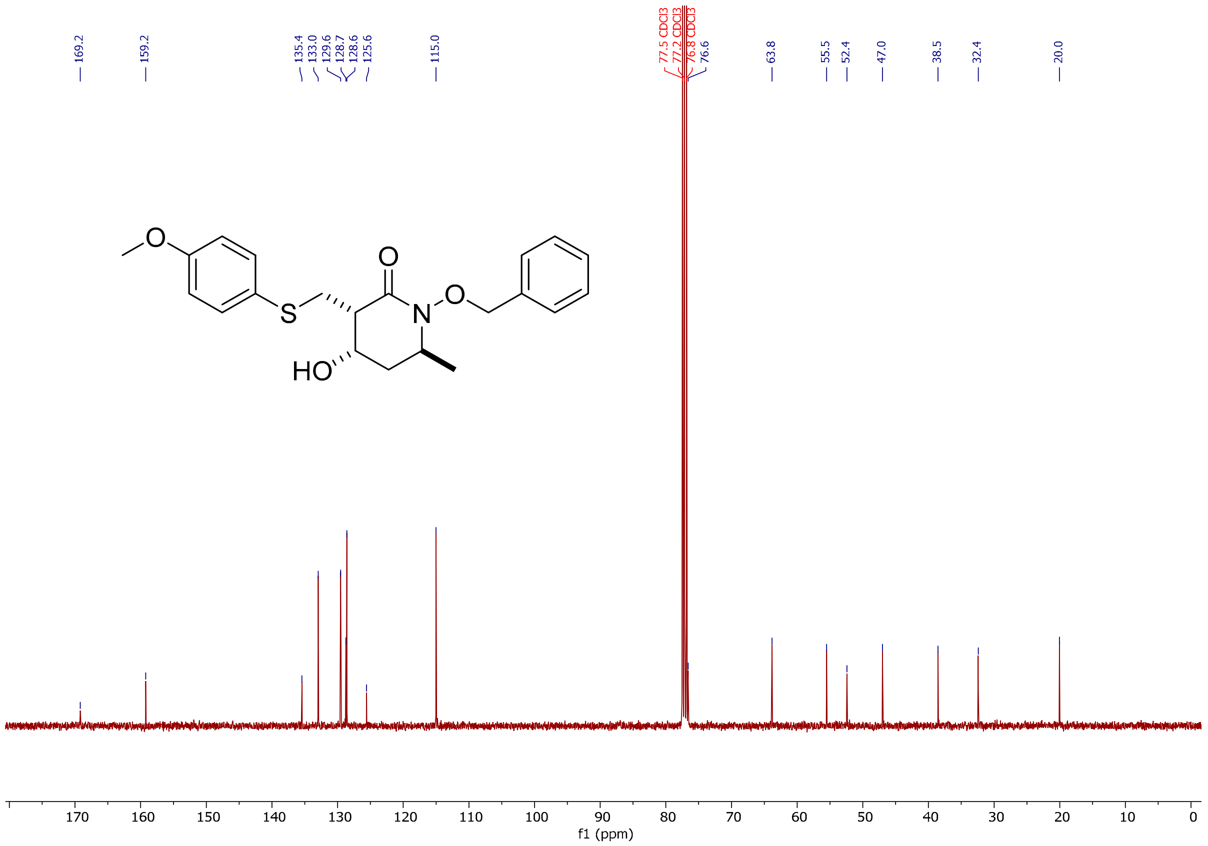

**(±)-(3*S*,4*S*,6*S*)-1-(Benzyloxy)-4-hydroxy-3-(((2-methoxyphenyl)thio)methyl)-6-methylpiperidin-2-one 4m**

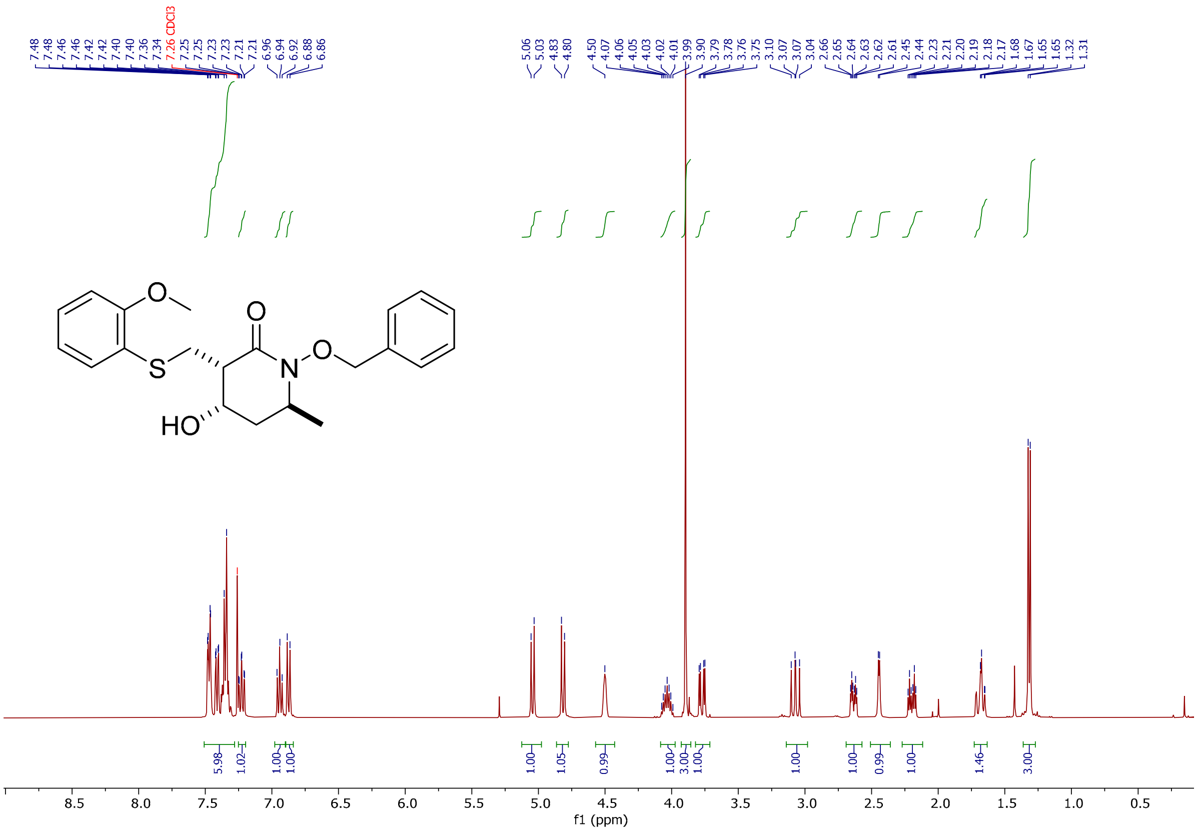

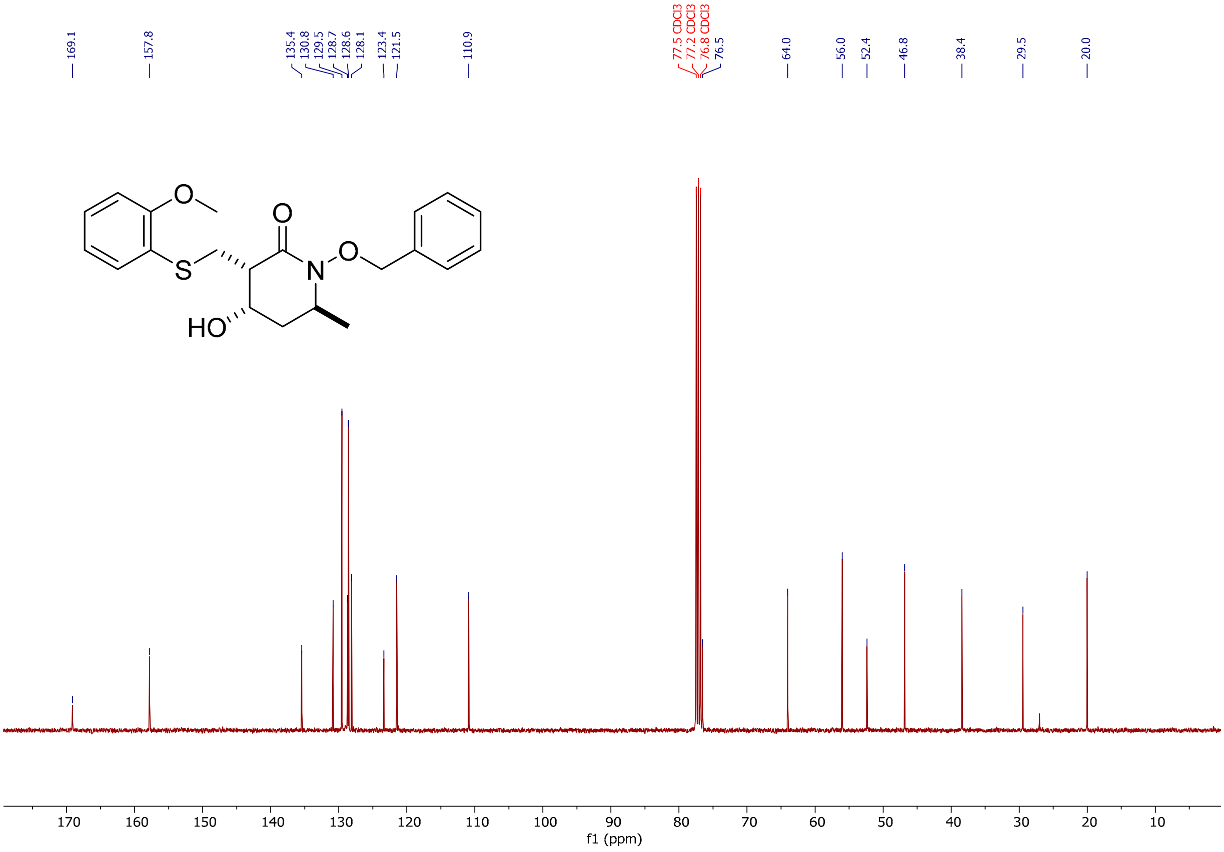

**(±)-(3*S*,4*S*,6*S*)-1-(Benzyloxy)-4-hydroxy-6-methyl-3-(((4-(trifluoromethyl)phenyl)thio)methyl) piperidin-2-one 4n**

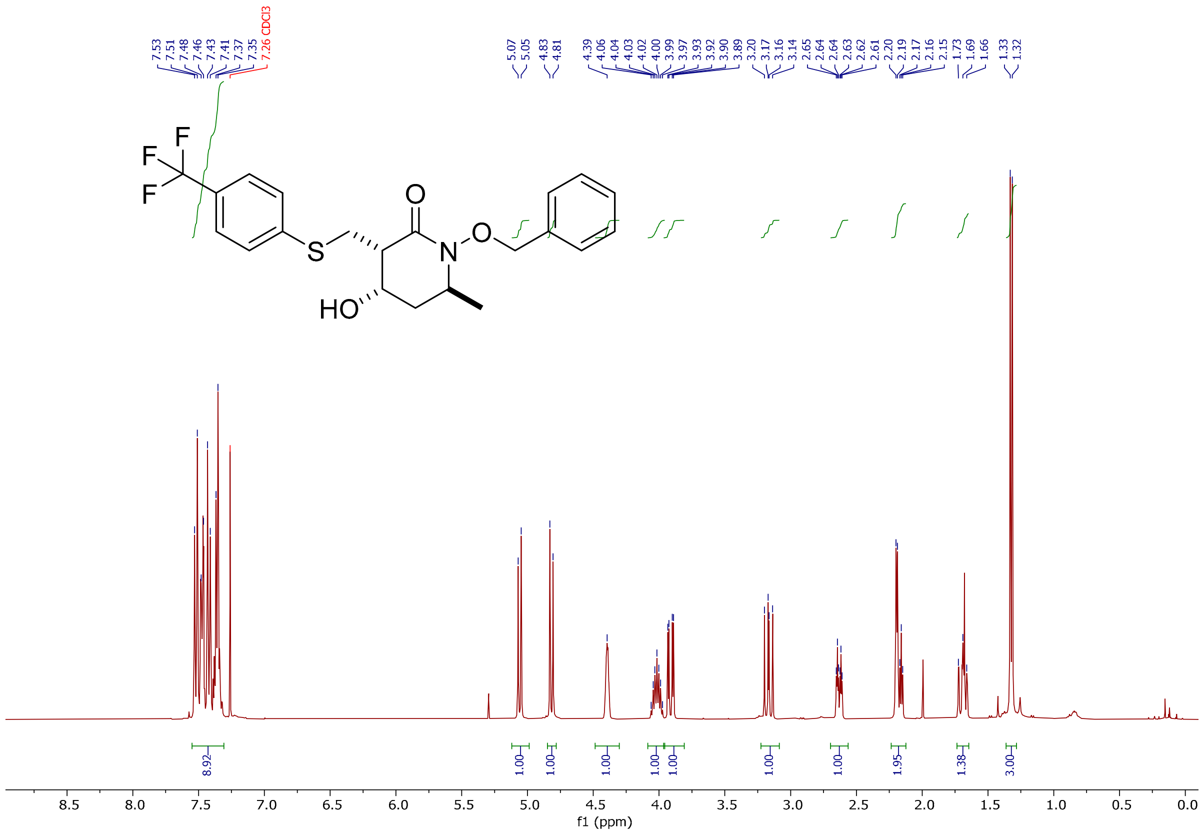

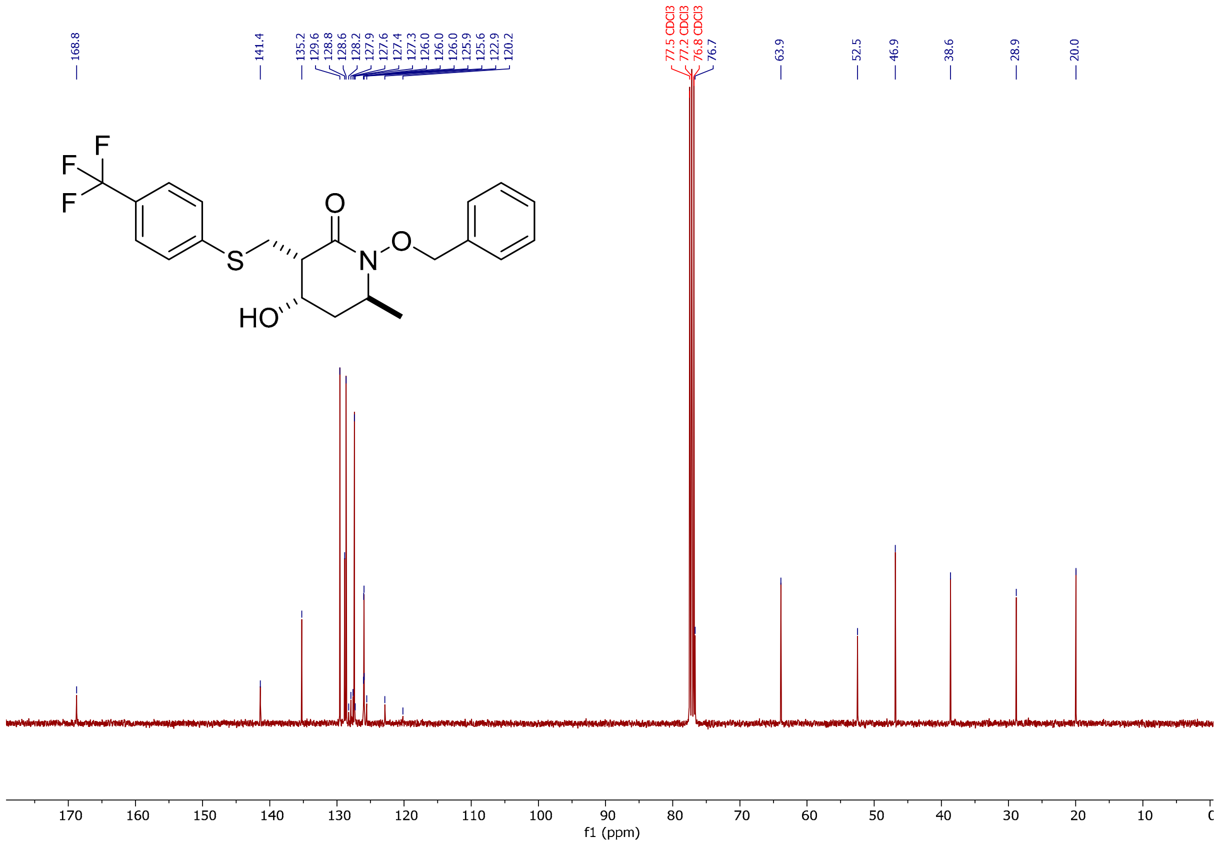

**(±)-(3*S*,4*S*,6*S*)-1-(Benzyloxy)-3-(((4-chlorophenyl)thio)methyl)-4-hydroxy-6-methylpiperidin-2-one 4o**

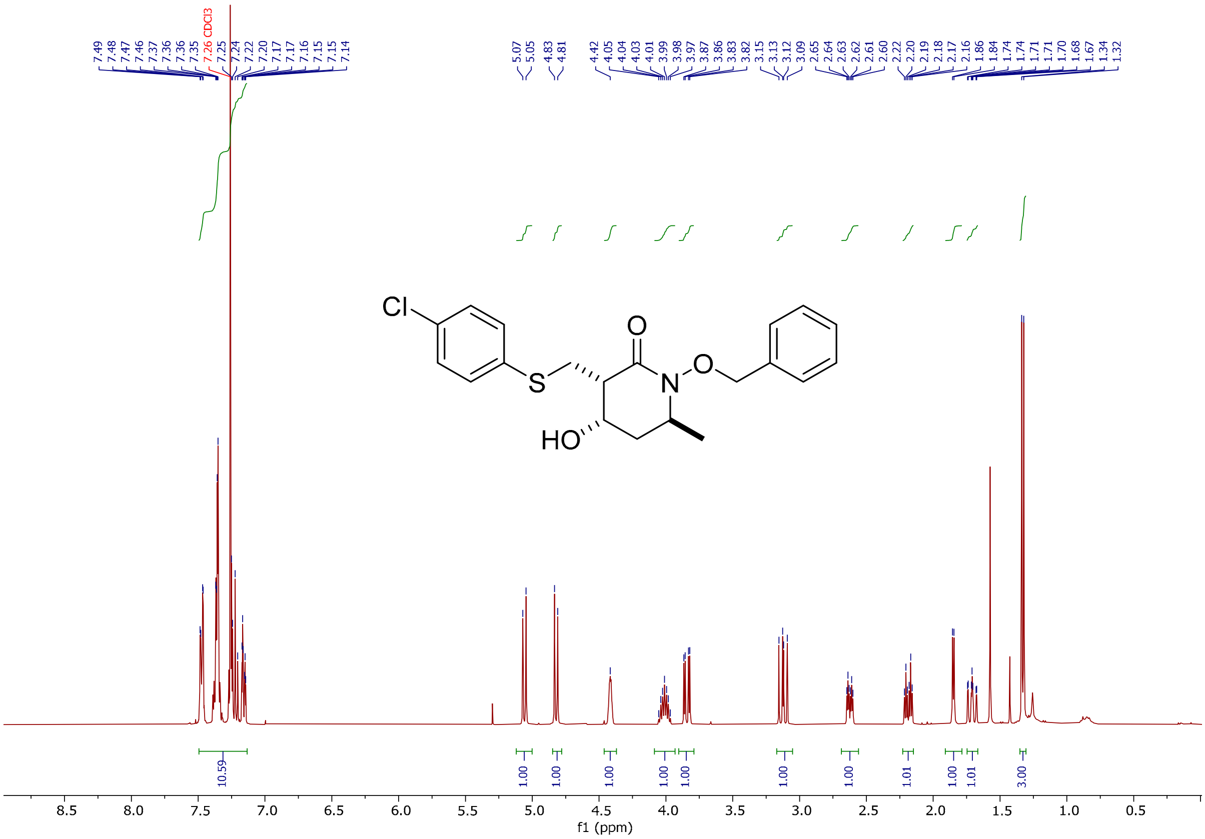

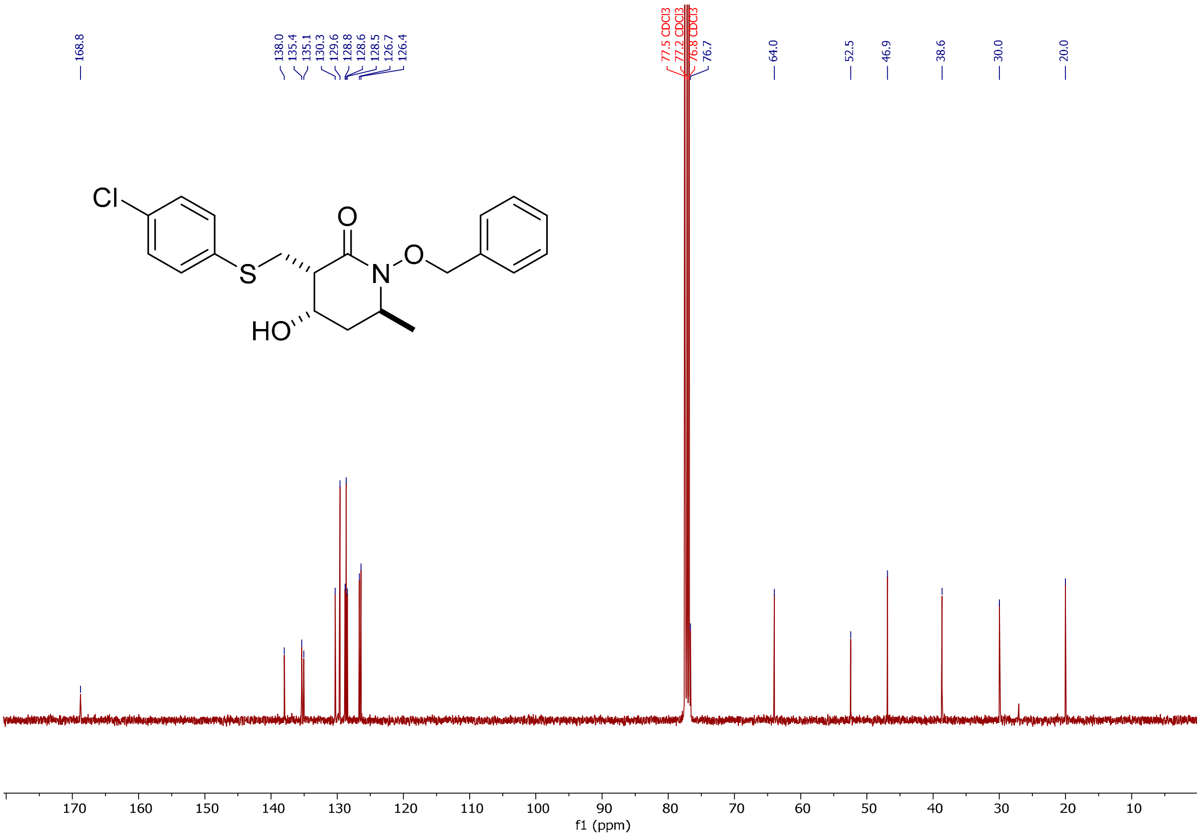

**(±)-(3*S*,4*S*,6*S*)-1-(Benzyloxy)-3-(((4-bromophenyl)thio)methyl)-4-hydroxy-6-methylpiperidin-2-one 4p**

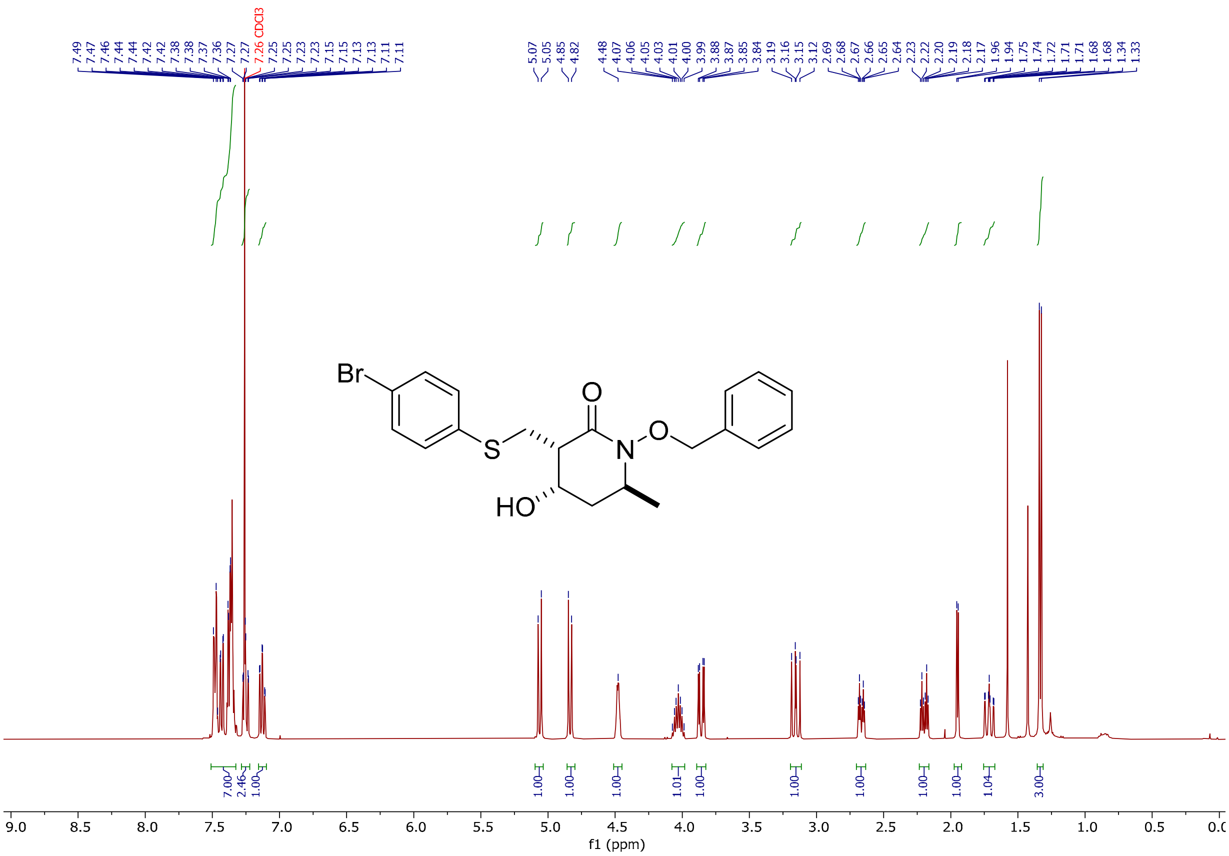

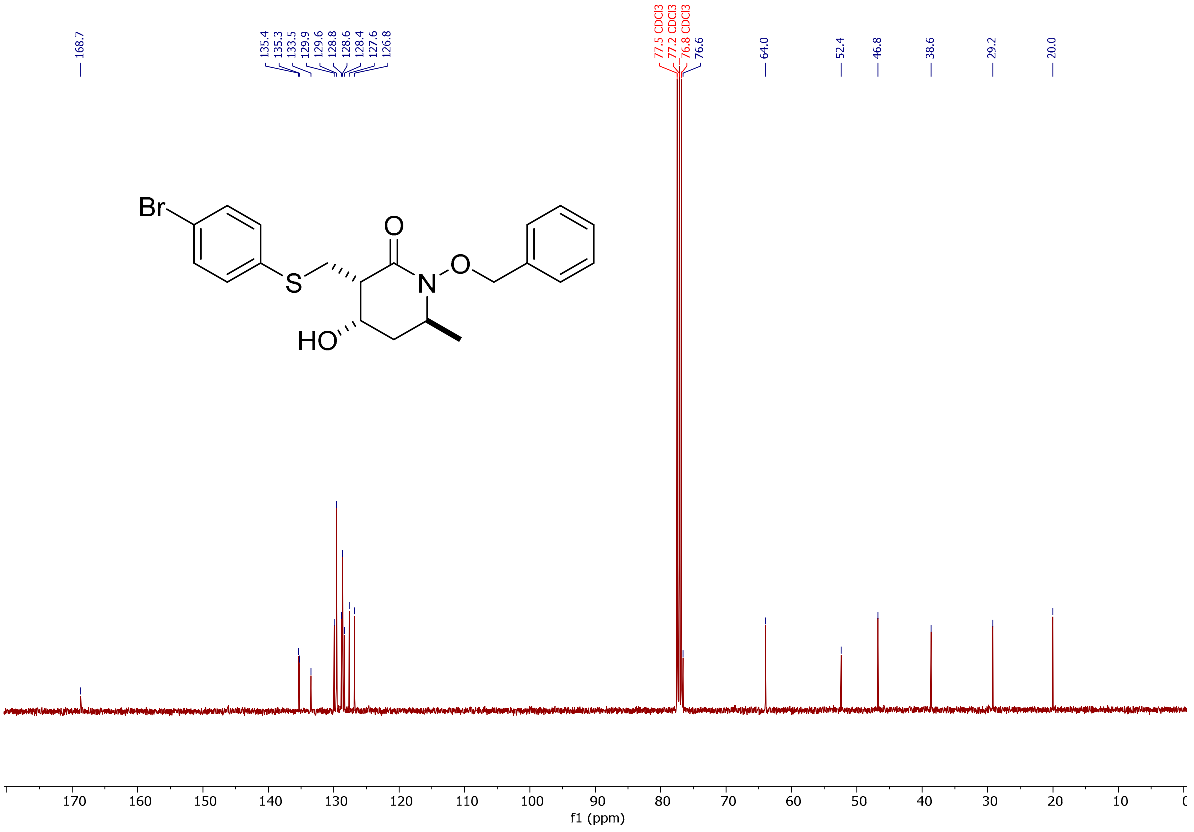

**(±)-(3*S*,4*S*,6*S*)-1-(Benzyloxy)-3-(((3-chlorophenyl)thio)methyl)-4-hydroxy-6-methylpiperidin-2-one 4q**

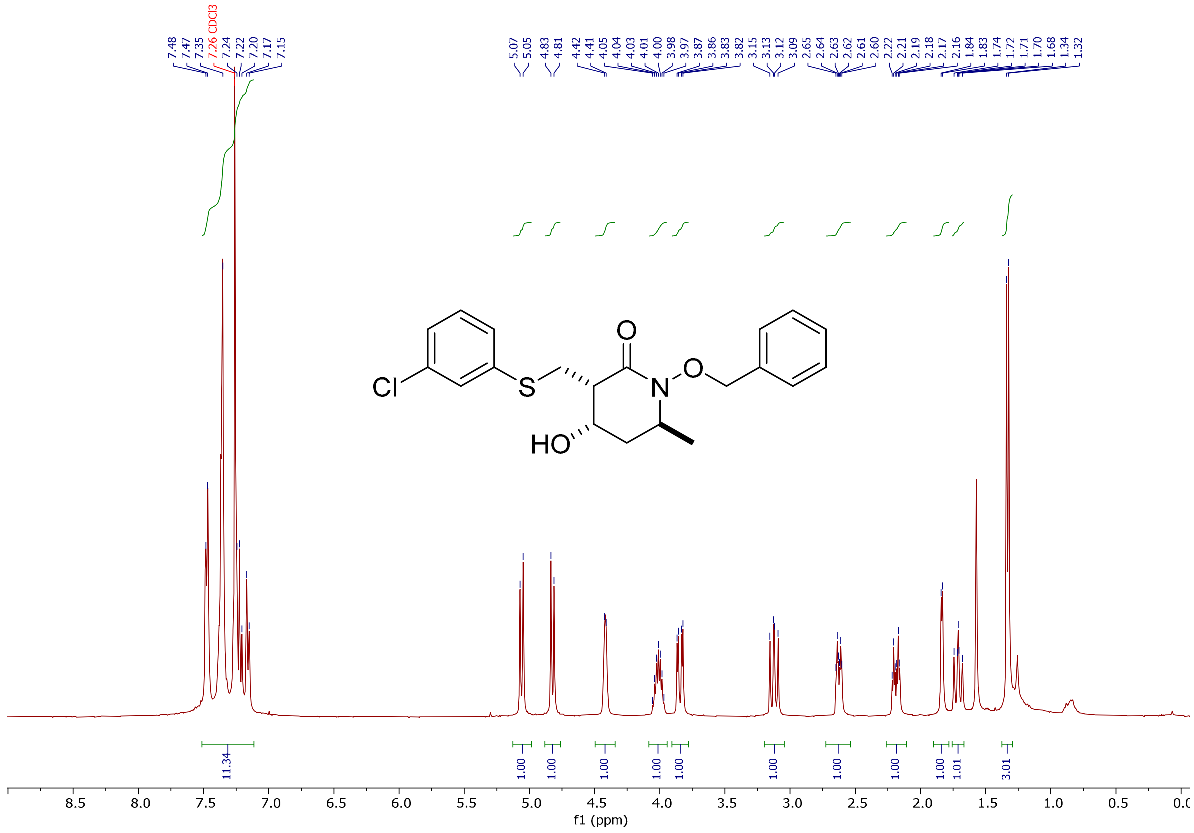


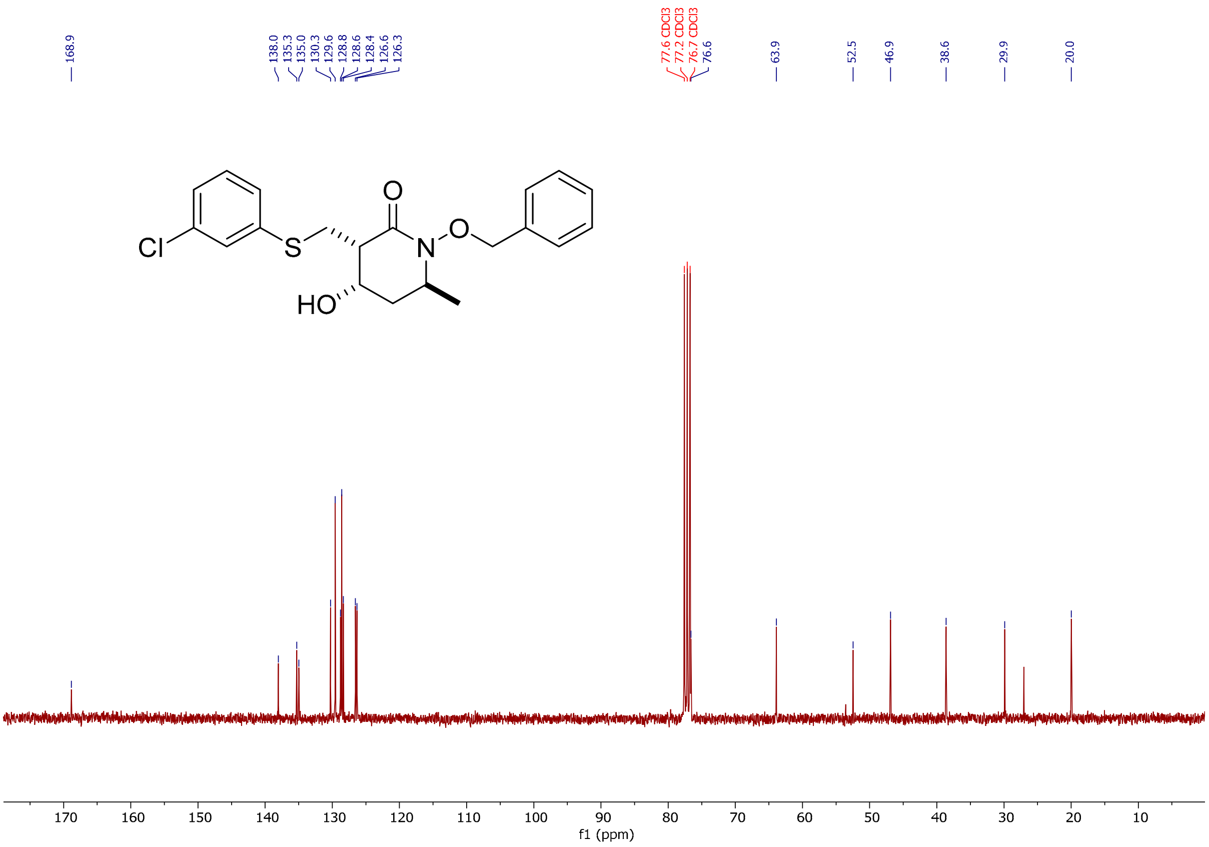

**(±)-(3*S*,4*S*,6*S*)-1-(Benzyloxy)-3-(((3-bromophenyl)thio)methyl)-4-hydroxy-6-methylpiperidin-2-one 4r**

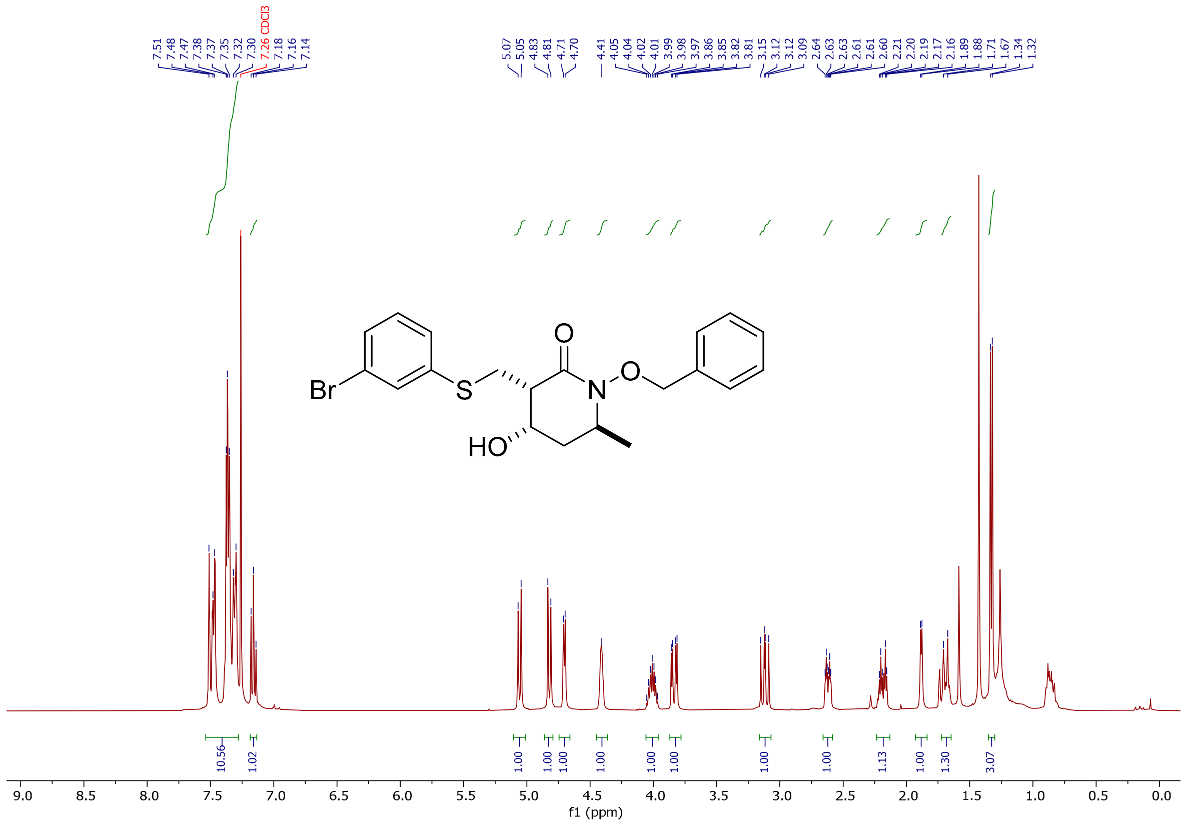

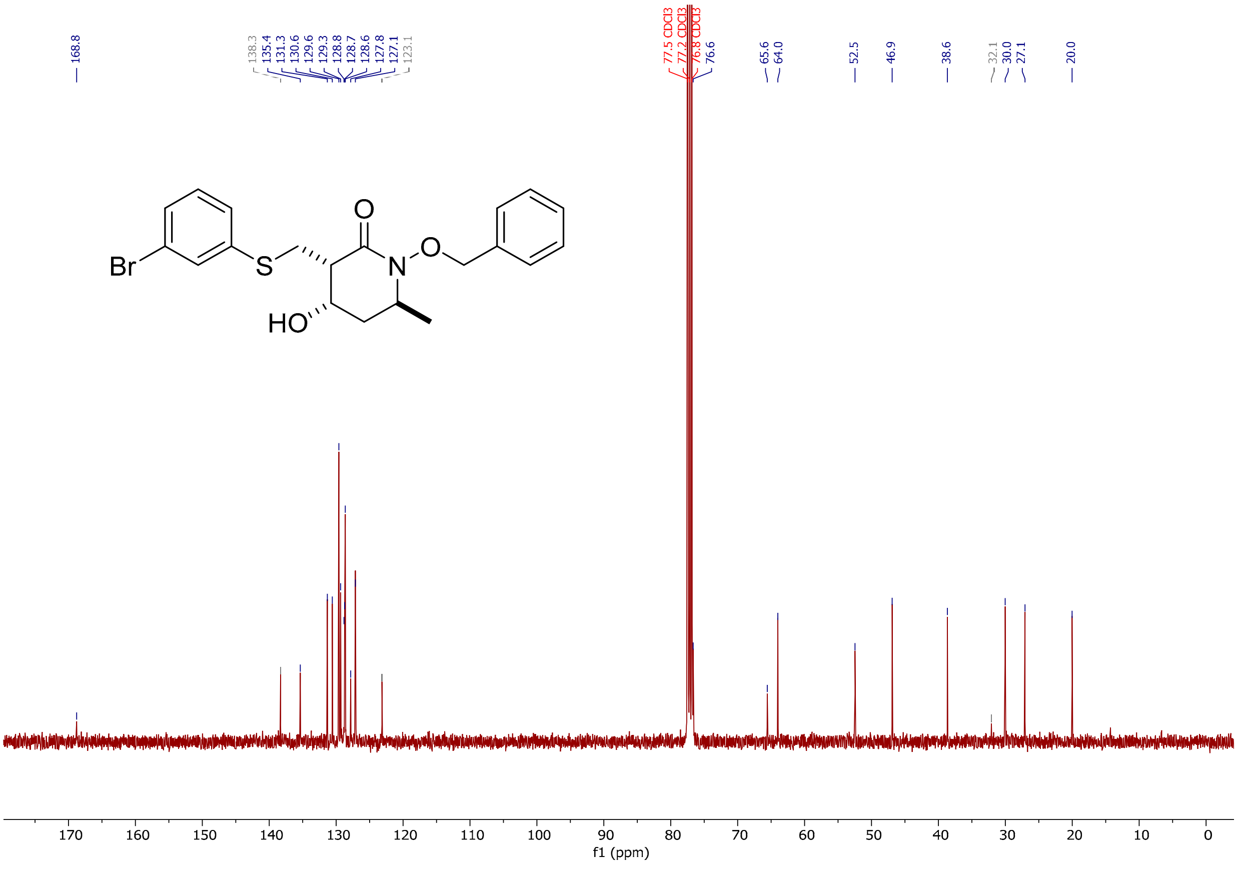

**(±)-(3*S*,4*S*,6*S*)-1-(Benzyloxy)-3-(((2-chlorophenyl)thio)methyl)-4-hydroxy-6-methylpiperidin-2-one 4s**

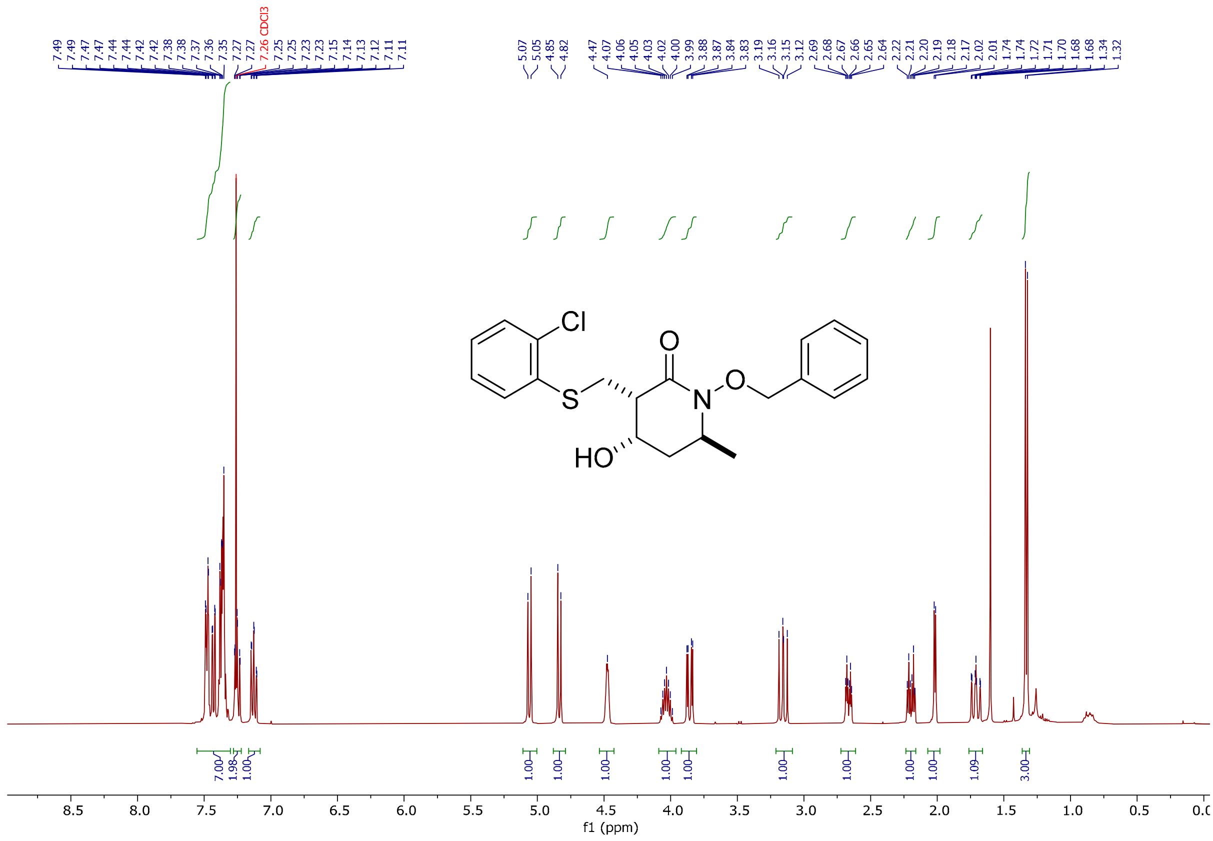


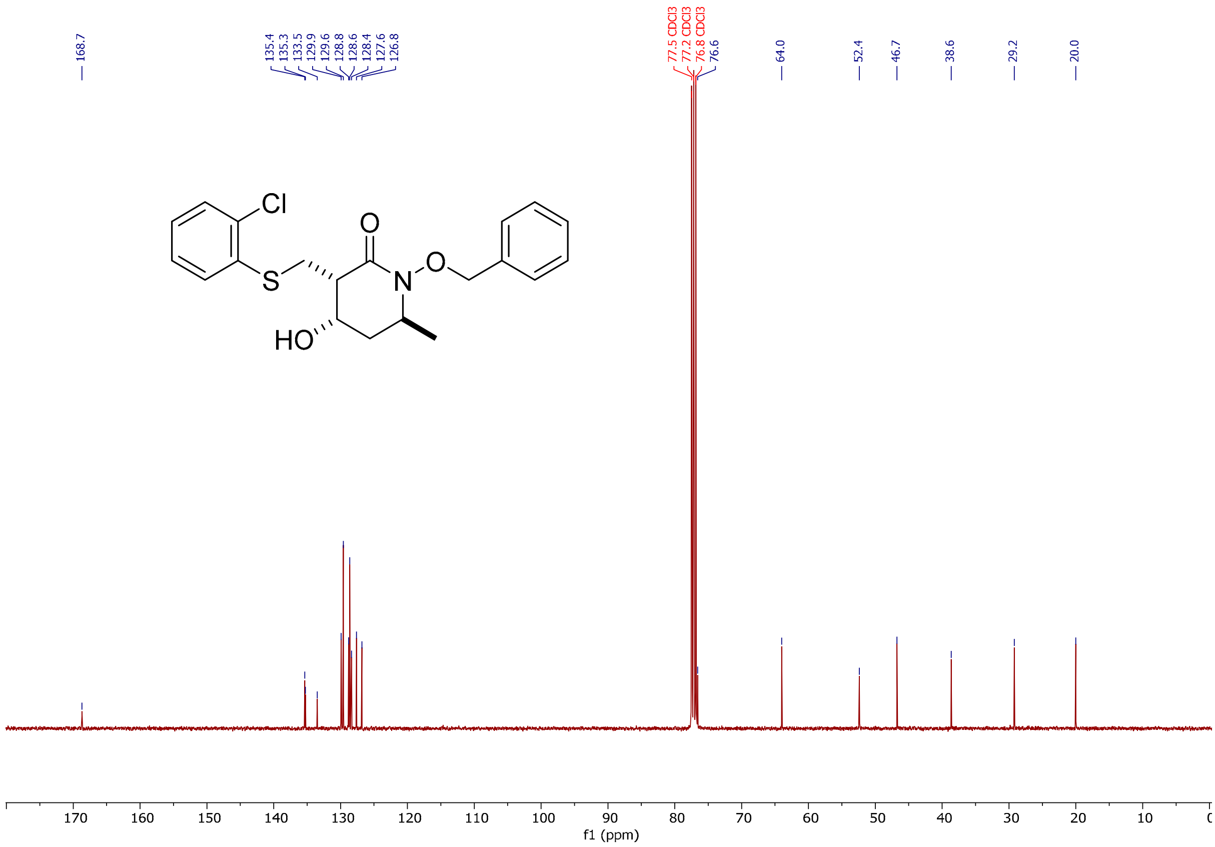

**(±)-(3*S*,4*S*,6*S*)-1-(Benzyloxy)-3-(((2-bromophenyl)thio)methyl)-4-hydroxy-6-methylpiperidin-2-one 4t**

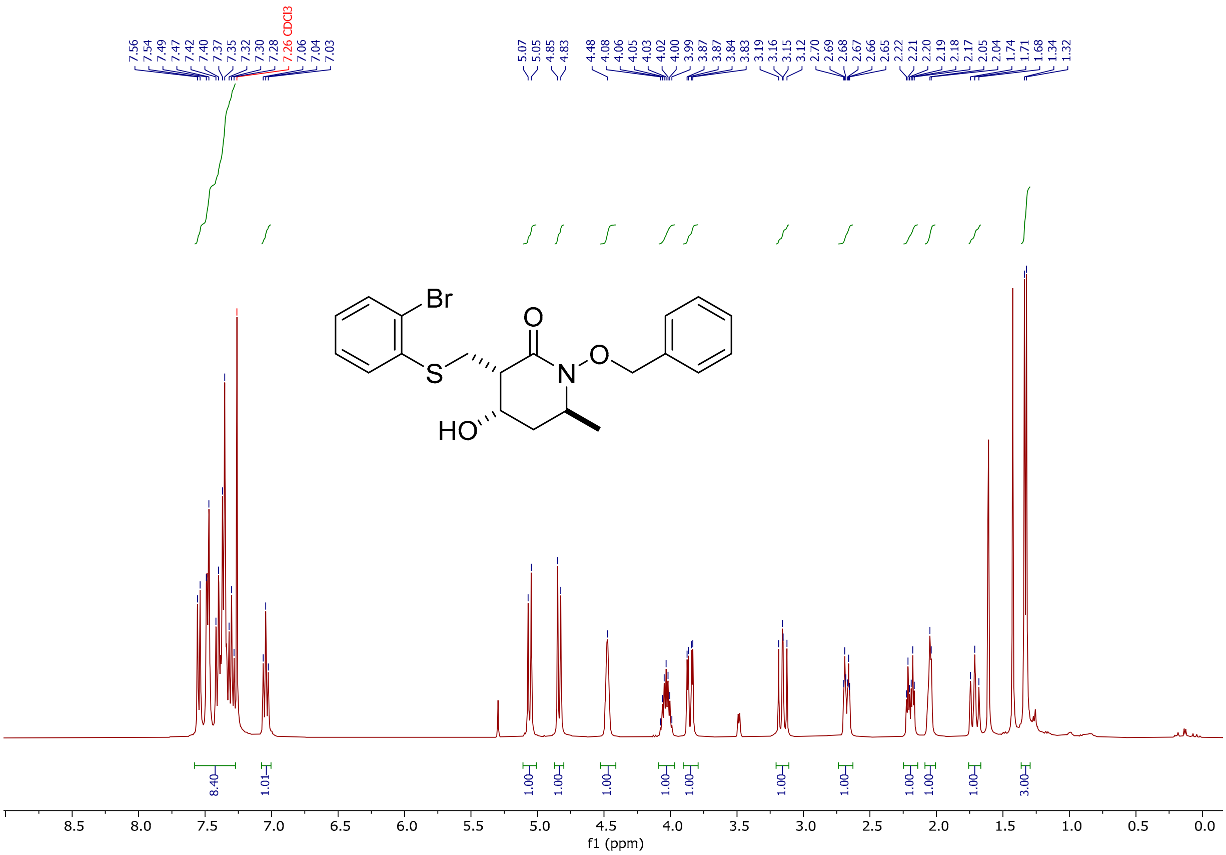


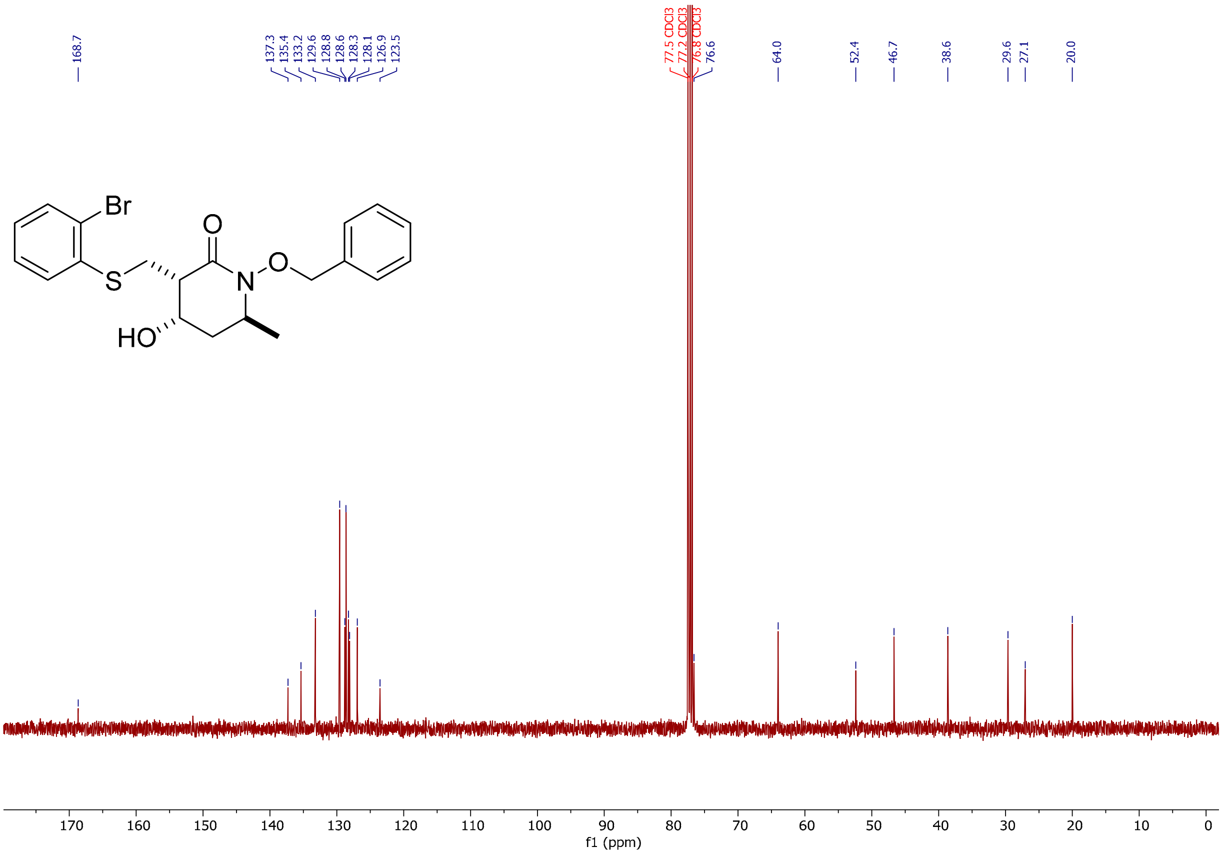

**(±)-(3*S*,4*S*,6*S*)-3-(((4-Aminophenyl)thio)methyl)-1-(benzyloxy)-4-hydroxy-6-methylpiperidin-2-one 4u**

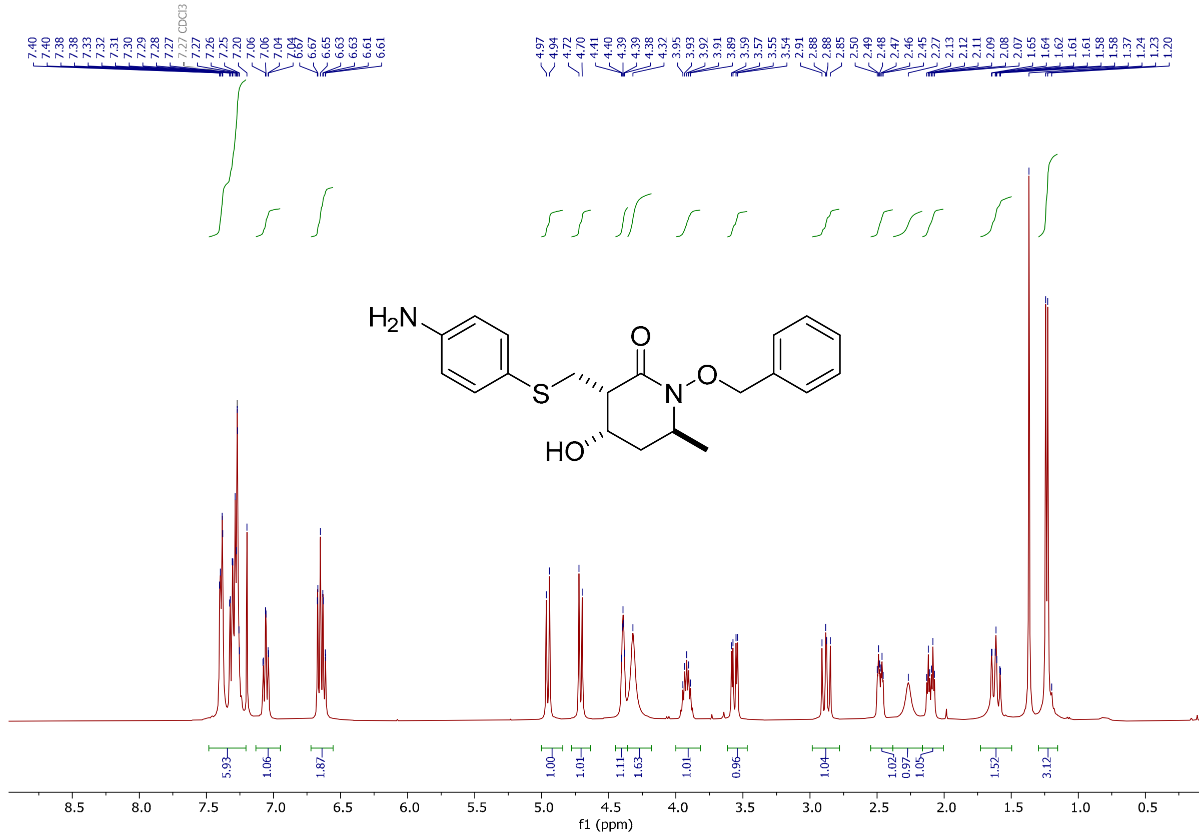

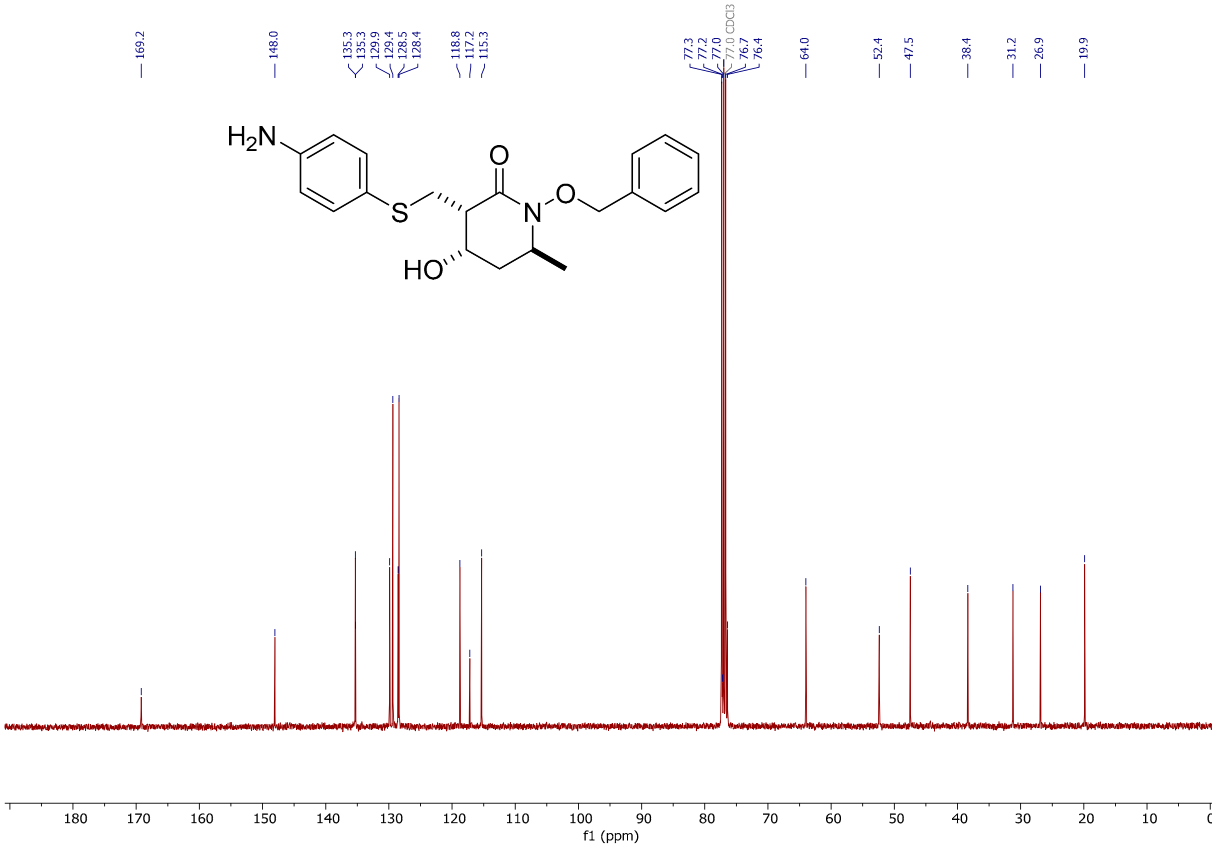

**(±)-(3*S*,4*S*,6*S*)-3-(((2-Aminophenyl)thio)methyl)-1-(benzyloxy)-4-hydroxy-6-methylpiperidin-2-one 4v**

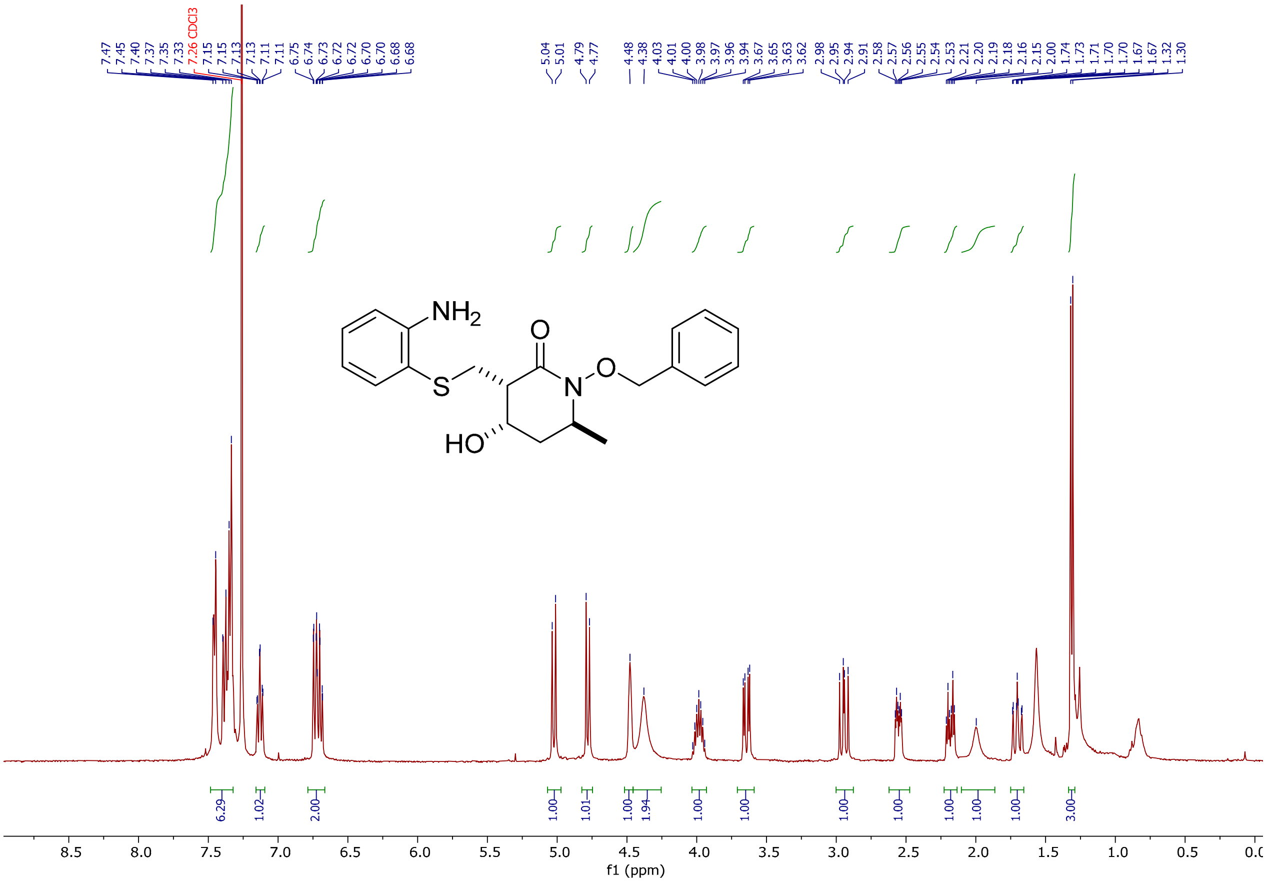

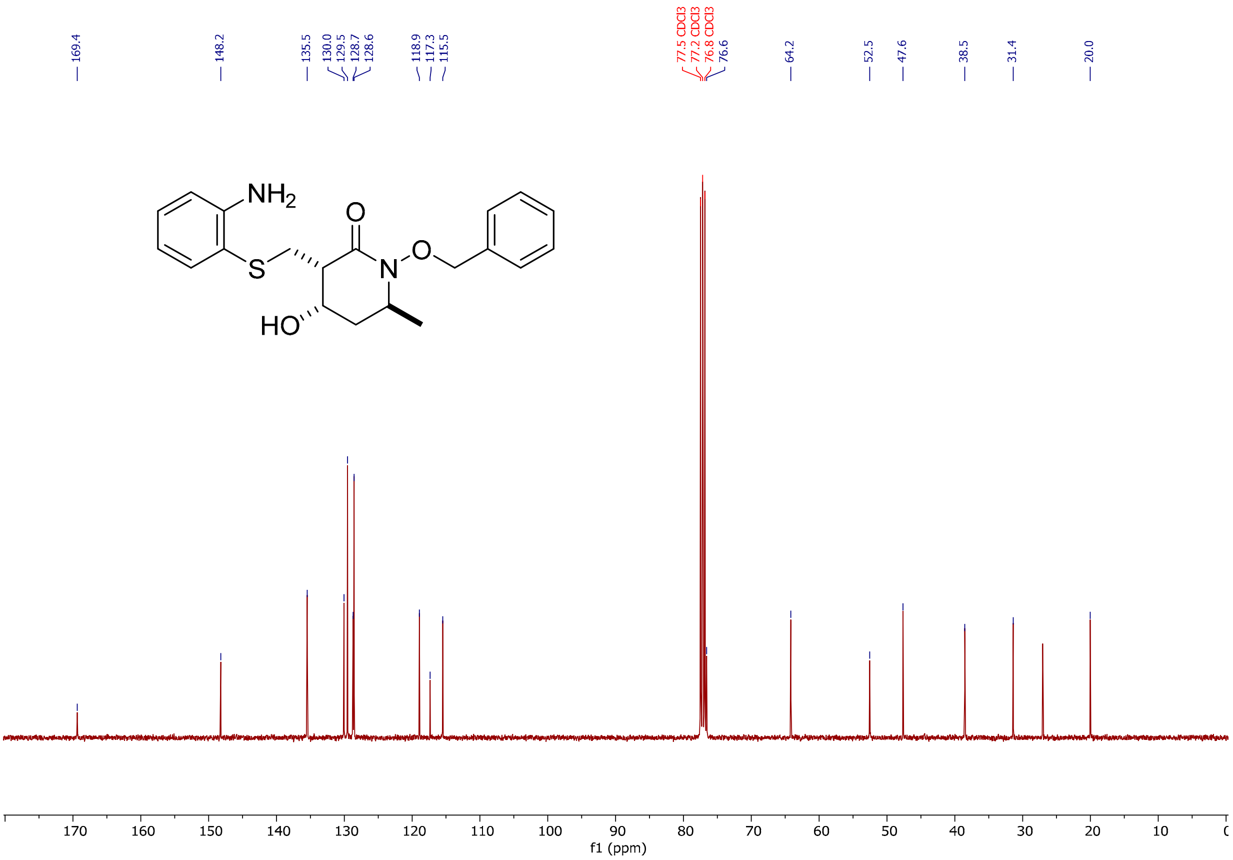

**(±)-(3*S*,4*S*,6*S*)-1-(Benzyloxy)-3-((benzylthio)methyl)-4-hydroxy-6-methylpiperidin-2-one 4w**

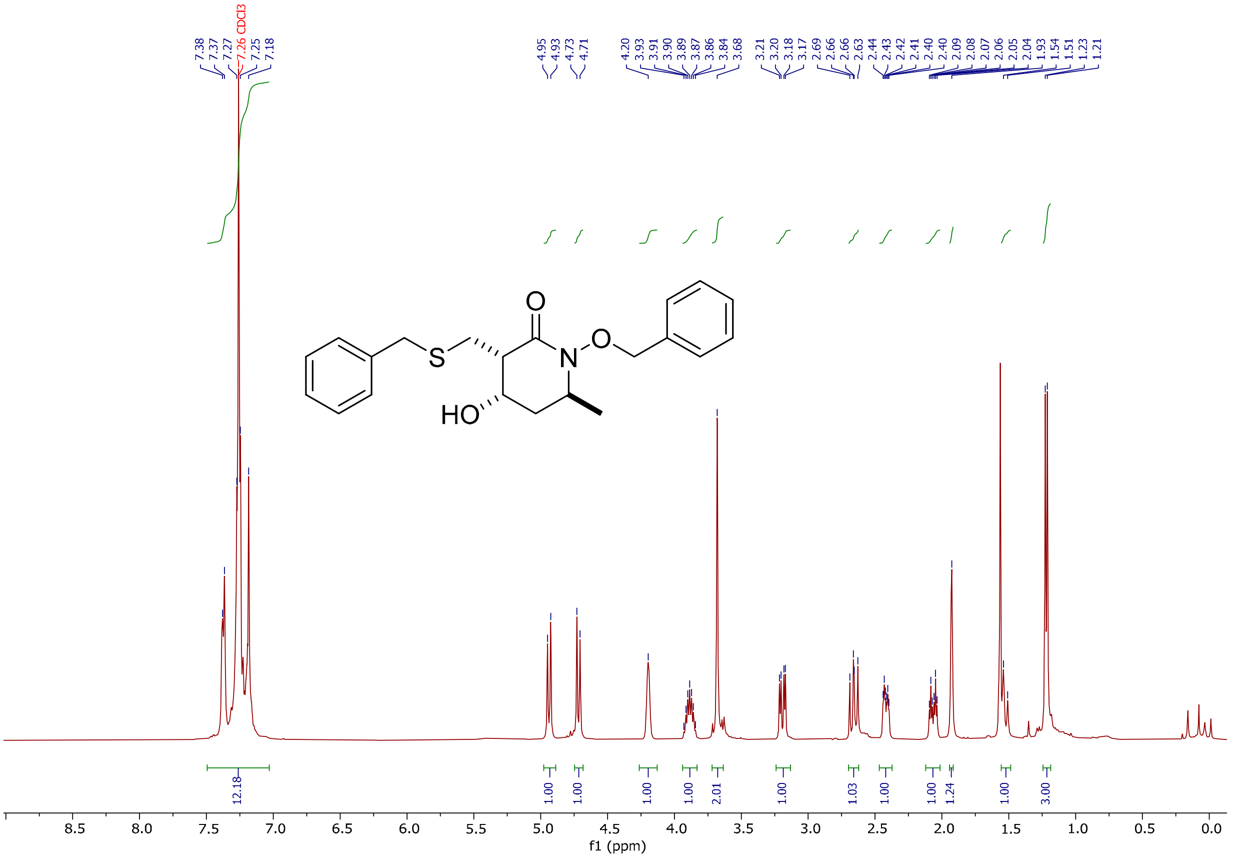

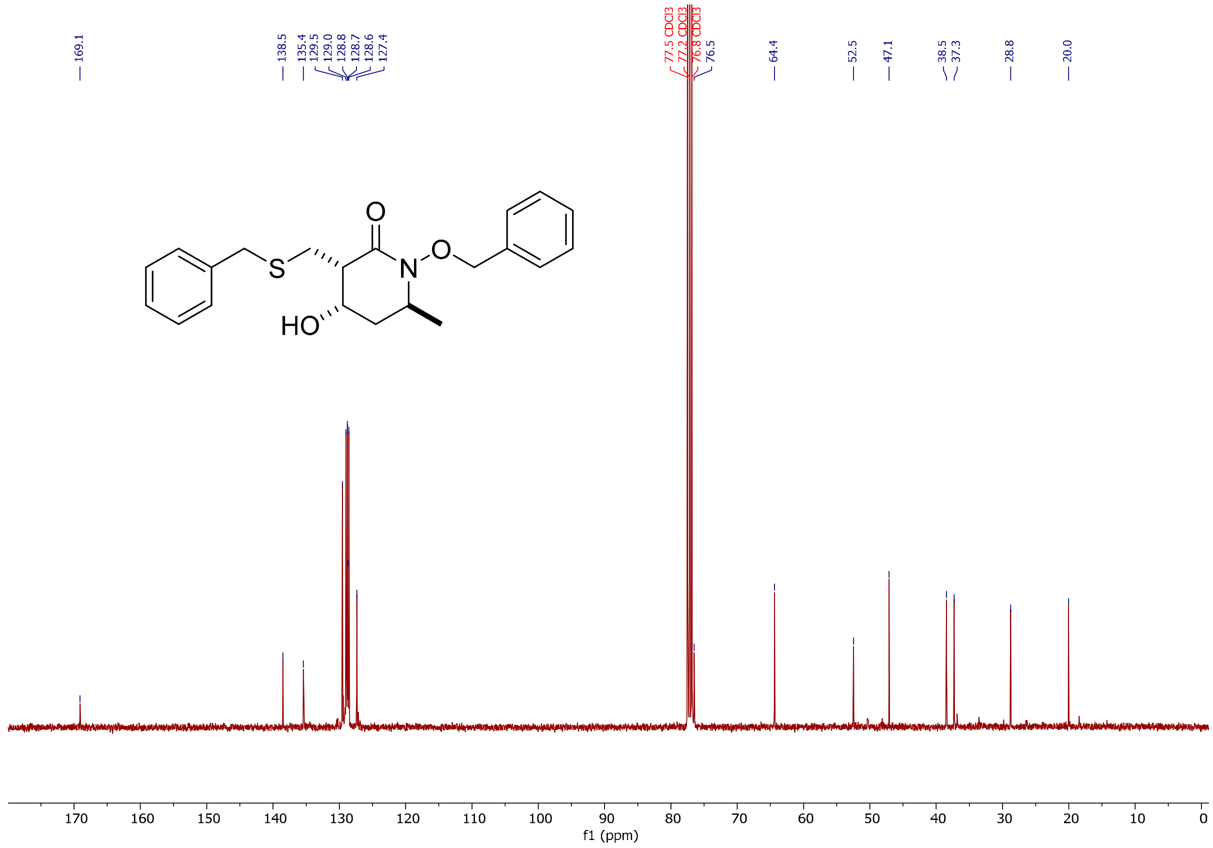

## (±)-(3*S*,4*S*,6*S*)-1-(Benzyloxy)-4-hydroxy-6-methyl-3-((propylthio)methyl)piperidin-2-one 4x

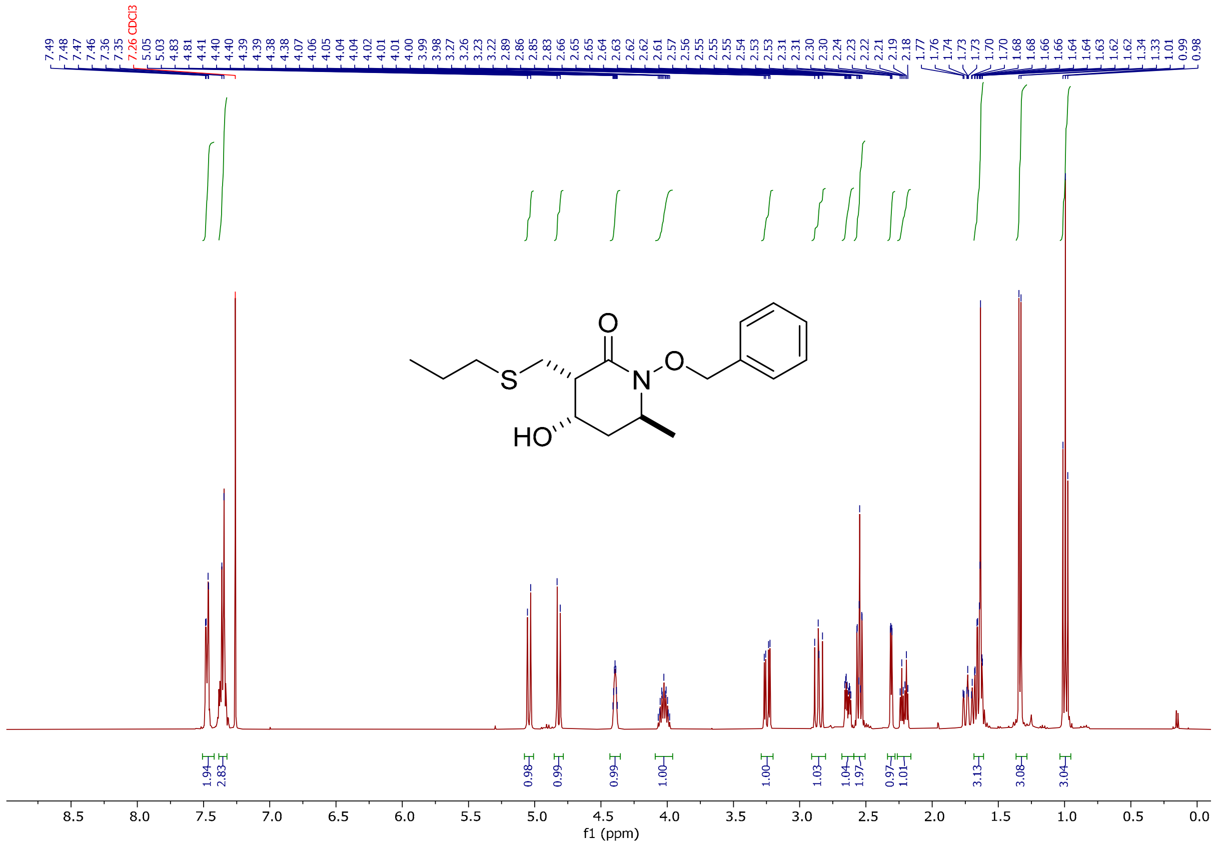

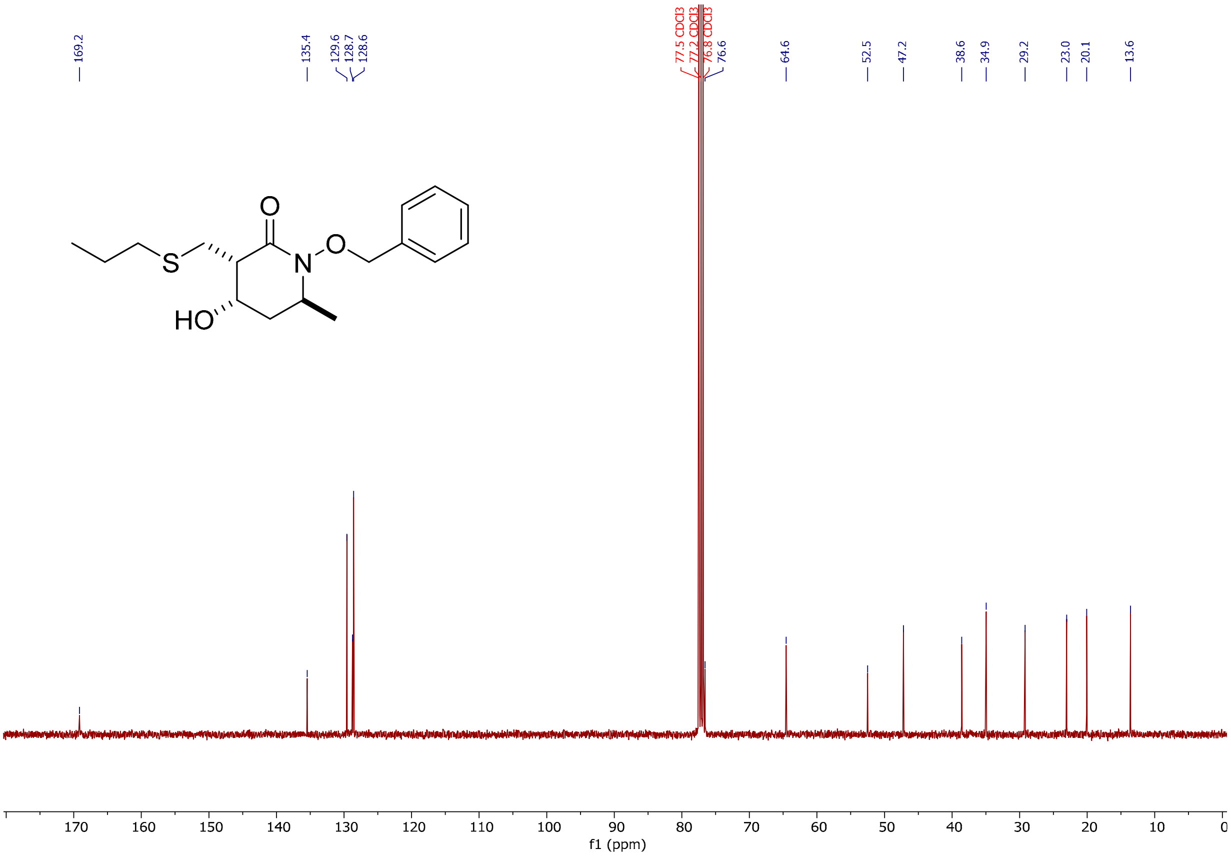
169.2
135.4
129.6
128.7
128.6
77.5 CDCl3
77.2 CDCl3
76.8 CDCl3
76.6
64.6
52.5
47.2
38.6
34.9
29.2
23.0
20.1
13.6
O
N
O
S
HO
170
160
150
140
130
120
110
100
90
80
70
60
50
40
30
20
10
0
f1 (ppm)
